# Seafood Toxicant Exposure During Pregnancy, Lactation, and Childhood and Child Outcomes: A Scoping Review

**DOI:** 10.1016/j.advnut.2024.100353

**Published:** 2024-12-10

**Authors:** Rupal Trivedi, Maureen K Spill, Sanjoy Saha, Rachel C Thoerig, Julie S Davis, Amanda J MacFarlane

**Affiliations:** 1 Division of AgriLife Research, Texas A&M Agriculture, Food and Nutrition Evidence Center, AgriLife Research, Fort Worth, TX, United States; 2 Department of Nutrition, Texas A&M University, College Station, Texas, United States

**Keywords:** seafood, toxicants, pregnancy, lactation, childhood, child outcomes, scoping review

## Abstract

Determining dietary recommendations for seafood consumed during pregnancy, lactation, and childhood requires consideration of the known nutritional benefits and potential harm due to toxicant exposure as they relate to child outcomes. This study aimed to describe the scope of the evidence associated with seafood-related toxicant exposure and child outcomes and to identify toxicant–outcome pairs that may have sufficient evidence to conduct a systematic review. We included studies examining seafood toxicant exposure during pregnancy, lactation, and childhood, and child outcomes. In total, 81 studies were included: 69 studies on exposure during pregnancy and lactation and 14 on exposure during childhood. The number of studies varied by toxicant and exposure population (maternal; child): mercury (*n* = 49; 7), methylmercury (*n* = 13; 3), polychlorinated biphenyls (PCBs; *n* = 11; 1), selenium (*n* = 11; 1), lead (*n* = 9; 3), perfluoroalkyl and polyfluoroalkyl substances (*n* = 8; 2), dichlorodiphenyltrichloroethane (*n* = 5; 1), arsenic (*n* = 4; 4), cadmium (*n* = 4; 4), zinc (*n* = 3; 2), polybrominated diphenyl ethers (*n* = 3; 1), dioxin-like compounds (*n* = 3; 0), iron (*n* = 2; 1), and magnesium (*n* = 1; 1). No studies examined polybrominated biphenyls, polycyclic aromatic hydrocarbons, iodine, aldrin, dieldrin, chlordane, chlorpyrifos, or microplastic exposures. Outcomes also varied by exposure population (maternal;child): neurodevelopment (*n* = 35; 9), child exposure biomarkers (*n* = 22; 4), growth (*n* = 17; 1), other adverse events (*n* = 4; 0), cardiometabolic (*n* = 3; 2), chronic disease indicators (*n* = 2; 0), and immune-related (*n* = 1; 2). Twelve maternal toxicant–outcome pairs had ≥3 studies, including mercury, methylmercury, lead, PCBs, perfluoroalkyl and polyfluoroalkyl substances, and arsenic as exposures and neurodevelopment, child exposure biomarkers, growth, and cardiometabolic as outcomes. For child exposure, only mercury and neurodevelopment had ≥3 studies. In conclusion, this scoping review shows relevant evidence for 14 of the 22 toxicants. Only 12 maternal and 1 child toxicant–outcome pairs, the majority of which examined maternal (methyl)mercury exposure, had ≥3 studies, our cutoff for consideration for systematic review. This scoping review indicates a paucity of research examining seafood toxicants beyond mercury and exposure during childhood. Systematic reviews are required to evaluate the associations for each toxicant–outcome pairs.

The protocol was registered at Open Science Framework (https://doi.org/10.17605/OSF.IO/FQZTA).

**Statement of Significance**

For exposure during pregnancy or lactation, 12 toxicant–outcome pairs had ≥3 studies; however, for seafood toxicant exposure during childhood, only 1 toxicant–outcome pair had ≥3 studies. A limited number of toxicant–outcome pairs had sufficient evidence to warrant conducting a systematic review.

## Introduction

Dietary guidelines provide recommendations for consuming seafood across the lifecycle, including during pregnancy, lactation, and childhood [1]. Seafood, defined as fish and shellfish, is an important lean and complete protein source [2] and an important source of ω-3 polyunsaturated fatty acids, vitamin D, iodine, selenium, zinc, magnesium, and calcium [[2], [3], [4], [5]]. Studies have demonstrated beneficial associations between seafood consumption and a number of health outcomes including allergic diseases, attention deficit and hyperactivity disorder, depressive symptoms, inflammatory bowel disease, neurodevelopment (i.e. cognition, intelligent quotient, memory, and processing speed), and cardiovascular health (i.e. hypertension and hyperlipidemia) [[6], [7], [8]].

Nonetheless, seafood is also a potential source of toxicants that may impact child outcomes. Bioaccumulation of toxicants occurs when larger fish consume contaminated plankton or smaller fish. In particular, bottom-dwelling fish may consume a high concentration of toxicants settled to the bottom floors of bodies of water [9]. Common toxicants found in seafood include heavy metals [10], persistent organic pollutants [11], pesticides [12], and microplastics [13]. Dietary guidance on seafood consumption during pregnancy, particularly, has cautioned against consuming seafood high in mercury [14]. However, mercury is only 1 of the several potential toxicants from seafood. Updated dietary guidance balances consideration for the nutritional benefits of seafood along with the risks associated with other potential toxicants in seafood.

The National Academies of Sciences, Engineering, and Medicine (NASEM) formed an expert Committee in 2022 to review the latest evidence and make recommendations for dietary guidance pertaining to seafood consumption and child development. The Committee identified 22 toxicants of concern related to seafood consumption and child outcomes [15]. Although systematic reviews are the gold standard approach to inform evidence-based decisions, they are time and resource intensive. Scoping reviews (ScRs) can provide information related to the amount of available literature on a topic to determine whether sufficient, relevant evidence exists to warrant a de novo systematic review, which in turn would be required to evaluate whether an association exists. Therefore, the objectives of this ScR were to *1*) estimate the amount of peer-reviewed evidence available on specific toxicants from seafood consumption and child outcomes, *2*) inform decisions on which toxicant exposure and outcome pairs have sufficient evidence for consideration for systematic review, and *3*) determine gaps in the existing literature.

## Methods

### Protocol development

The NASEM formed an expert committee in 2022 with the goal of assessing relationships between seafood toxicants consumed during pregnancy, lactation, and childhood, and child outcomes. The committee prioritized 22 seafood toxicants for review (Table 1) [15]. The prioritized toxicants were assessed in this ScR to determine which toxicants and outcomes had sufficient evidence to warrant further assessment. Although there is no minimum number of studies required to conduct a systematic review, for the purposes of this review, and in agreement with the NASEM committee, we defined sufficient evidence to be 3 or more studies on a seafood toxicant–outcome pair. The protocol was registered in Open Science Framework (https://doi.org/10.17605/OSF.IO/FQZTA) [16].

**TABLE 1:** Inclusion and exclusion criteria for the scoping review.

Category	Inclusion criteria	Exclusion criteria
Population	Human individuals living in countries ranked as high or very high on the human development index^1^ during the study. Exposed population: Individuals in the general population who are pregnant or lactating, infants, children, or adolescents aged 18 y or younger. Outcome population: Children and adolescents (aged 18 y or younger).	Studies exclusively of participants with a chronic condition, hospitalized with an illness or injury. Examples include the following: • Diabetes (not including gestational diabetes) • Cancer • Cardiometabolic disorders • Chronic kidney disease • Malabsorption (any disorder that causes malabsorption from the gastrointestinal tract) • Asthma Studies involving nonhuman primates
Exposure	Studies must contain Exposure 1 AND Exposure 2 Exposure 1: Toxins or toxicants • Persistent organic pollutants: polychlorinated biphenyls (PCBs), dioxins or dioxin-like compounds, polybrominated biphenyls, polybrominated diphenyl ethers, polycyclic aromatic hydrocarbons (PAHs), perfluoroalkyl and polyfluoroalkyl substances • Metals: methylmercury, mercury, arsenic, cadmium, and lead • Essential trace elements: selenium, iron, magnesium, iodine, and zinc • Pesticides: dichlorodiphenyltrichloroethane, aldrin, dieldrin, chlordane, and chlorpyrifos • Microplastics Exposure 2: Seafood consumption • Types (e.g. salmon, tuna, bass) • Sources (e.g. sea, fresh water, farmed, canned, wild) • Amount (e.g. ounces per day, grams per meal) • Frequency (e.g. daily, twice a week) • Duration (e.g. length of time consuming seafood) • Preparation (e.g. fried, baked) • Timing (e.g. by trimester, age) Studies must show statistical relationships between seafood consumption and child outcomes or between toxicants and child outcomes.	Studies that do not report on toxicant exposure in fish AND seafood consumption Studies that report on supplements or infant formula Studies that report on toxins from algal blooms (cyanobacteria, ciguatera, scombroid, or domoic acid [red algae]) Studies that report on microorganisms (hepatitis, salmonella, *Escherichia coli*) Studies that provide descriptive data only for seafood, toxicants, or child outcomes
Comparator	Toxins or toxicants • Studies that compared exposures at different levels of the toxins or toxicants of interest. • Studies that compared an exposure with no exposure of interest. Seafood consumption • Studies that compared different types, sources, amounts, frequencies, durations, preparations, or timings of seafood consumption. • Studies that compared seafood consumption to no seafood consumption.	Studies that do not make any comparisons
Outcome	Child exposure biomarkers: • Metals (As, Cd, Cr, Cu, Hg, Ni, Pb, Zn), polycyclic hydrocarbons (PAHs, PCBs), immunologic parameters (alanine aminotransferase, aspartate aminotransferase), neurotoxic parameters (choline esterase), others (benzo(a)pyrene, phenanthrene, and domoic acid). Neurodevelopment and neurodevelopmental disorders: • Developmental domains (cognition, language or communication, movement or physical, social-emotional), social or emotional outcomes, academic performance, autism spectrum disorders, anxiety, depression, attention deficit hyperactivity disorder Growth: • Measure of growth and body composition, failure to thrive (malnutrition, protein deficiency) Cardiometabolic related: • Blood pressure, dyslipidemia Immune-related: • Allergy and immune response, asthma, autoimmune diseases Chronic disease indicators: • Cancer, other Other adverse effects: • Captured based on information provided in the included studies	Studies that do not involve eligible outcomes
Study design	Randomized controlled trials, controlled (nonrandomized) trials, prospective or retrospective cohort studies, case–cohort studies, case–control studies, before–after studies	Case reports, studies reported in theses or conferences abstracts only, studies without primary data (i.e. systematic and narrative reviews, editorials, and commentaries), cross-sectional studies
Language	Studies reported in the English language	Studies not reported in English

### Study selection criteria

**FIGURE 1:**
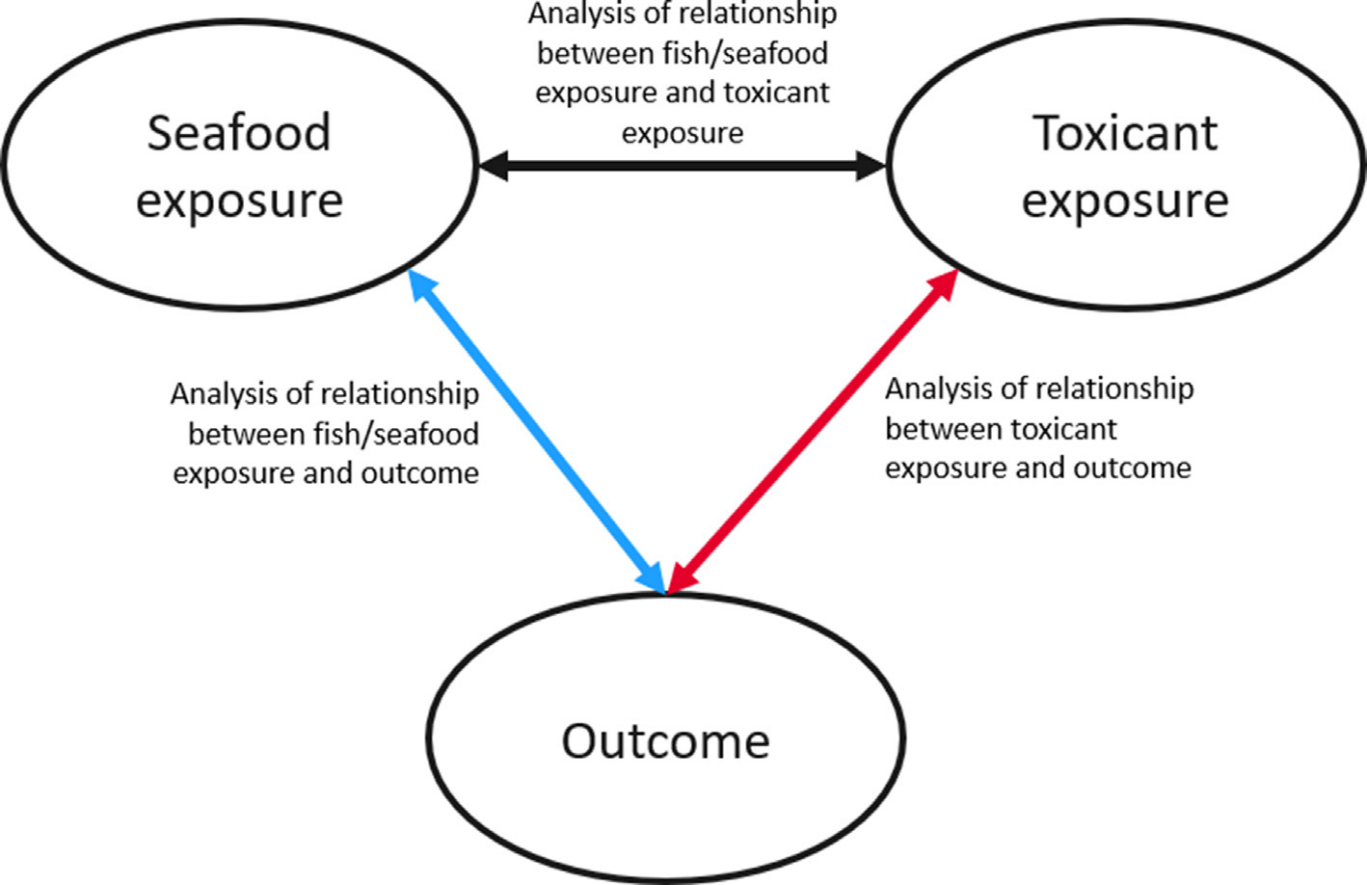
Inclusion criteria regarding analyses between exposures and outcomes: include an article if it reports analyses on ≥2 relationships among the 3 possible between seafood exposure, toxicant exposure, and the outcome.

Studies were selected into this ScR based on a predetermined Population, Exposure, Comparator, Outcome, and study Design framework. To be included, studies had to measure seafood consumption and concentrations of a seafood-related toxicant during pregnancy, lactation, or childhood in countries rated high or very high on the Human Development Index [17]. A full description of inclusion and exclusion criteria, including the eligible toxicants and outcomes, is available in Table 1. Studies could compare different types, sources, amounts, frequencies, durations, preparations, or timings of seafood consumption, or different concentrations of toxicant exposures. Toxicants were organized into 5 categories: persistent organic pollutants, metals, pesticides, microplastics, and essential trace elements. Trace elements were included because although their intake is required to achieve adequacy, intakes above the tolerable upper intake level can lead to toxicity, and higher exposures have been negatively associated with child outcomes [15,[18], [19], [20]]. Outcome categories included the following: *1*) child exposure biomarkers, *2*) neurodevelopment and neurodevelopmental disorders, *3*) growth outcomes, *4*) cardiometabolic-related outcomes, *5*) immune-related outcomes, *6*) chronic disease indicators, and *7*) other child outcomes. To be included, studies had to measure seafood and toxicant exposure, analyze the relationship with each other and/or with the child outcome (Figure 1). Randomized controlled trials, controlled nonrandomized trials, prospective or retrospective cohort studies, case–cohort studies, case–control studies, or before and after studies were eligible study designs if they were published in the English language. Studies that only provided descriptive data, cross-sectional studies, and nonhuman studies were excluded.

### Search and screening strategy

An experienced librarian developed and performed the search strategy for this ScR (Supplemental Table 1). Medical Subject Heading terms for seafood, various toxicants, and child outcomes were searched in the EMBASE, PubMed, and Cochrane Central Register of Controlled Trials (CENTRAL) databases. The search date range was January 2000 to July 2023.

Two reviewers used DistillerSR to independently screen titles, abstracts, and full text of each record to determine its eligibility based on predetermined inclusion and exclusion criteria (Table 1). The reviewers reconciled any disagreements during screening through a discussion or through the input of another reviewer. Supplemental Table 2 shows reasons for excluding full-text articles.

### Data extraction

Reviewers extracted study characteristics including study design, cohort name (if applicable), country, and sample size. Participant characteristics were extracted including toxicant exposure(s) measured and analyzed, child outcome(s) assessed, the exposure population, race/ethnicity, socioeconomic status, infant feeding practices, maternal age and anthropometrics, and child age, percent female, and anthropometrics. For the purpose of this study, child exposure biomarkers assessed through cord blood samples were considered to be maternal markers. Through a dichotomous indication (yes or no), reviewers determined whether the included studies reported the following toxicant exposures: persistent organic pollutants, heavy metals, essential trace elements, pesticides, and microplastics. Similarly, the reviewers indicated dichotomously (yes or no) if the following study outcomes were assessed: child exposure biomarkers, neurodevelopmental outcomes, growth outcomes, immune-related outcomes, chronic disease indicators, and other outcomes. Finally, to inform future reviews, the reviewers extracted information on confounders or covariates directly from each study as they were provided by the authors.

A key objective of this review was to identify toxicant exposure and outcome pairs that had sufficient evidence to warrant conducting a de novo systematic review. For this purpose, we defined sufficient evidence to be 3 or more studies on any toxicant–outcome pair that reported an analysis including seafood intake, toxicant exposure, and child outcomes within a specific population (maternal or child).

## Results

### Literature search results

**FIGURE 2:**
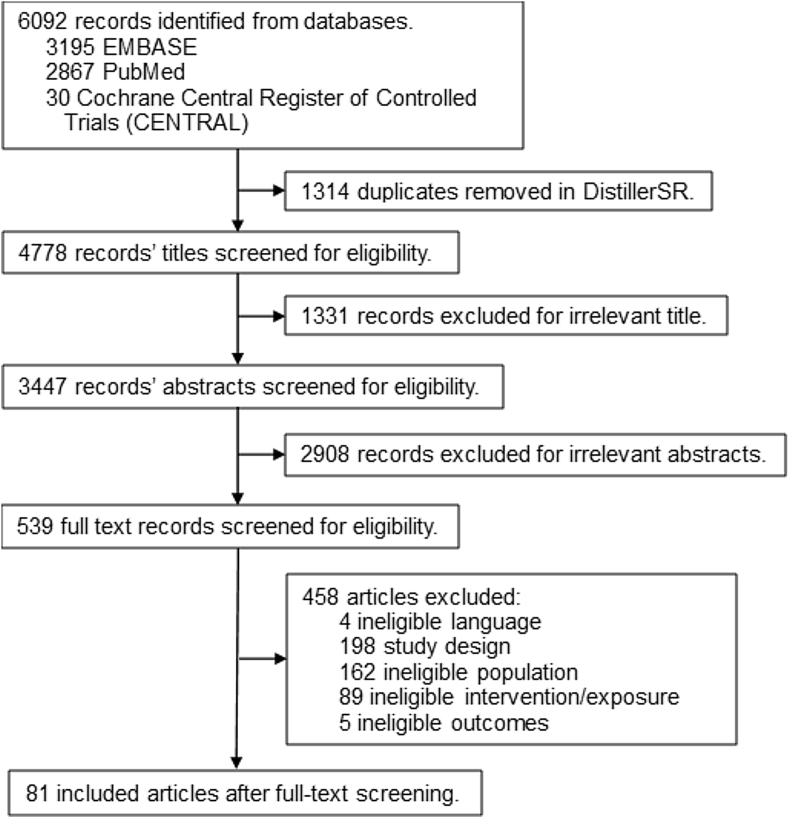
Screening flow diagram.

The search identified 4778 unique records, with 81 included after screening (Figure 2) [[21], [22], [23], [24], [25], [26], [27], [28], [29], [30], [31], [32], [33], [34], [35], [36], [37], [38], [39], [40], [41], [42], [53], [43], [44], [45], [46], [47], [48], [49], [50], [51], [52], [54], [55], [56], [57], [58], [59], [60], [61], [62], [63], [64], [65], [66], [67], [68], [69], [70], [71], [72], [73], [74], [75], [76], [77], [78], [79], [80], [81], [82], [83], [84], [85], [86], [87], [88], [89], [90], [91], [92], [93], [94], [95], [96], [97], [98], [99], [100], [101]]. We documented reasons for exclusion at the full-text level (Supplemental Table 2).

### Study and participant characteristics

The included articles were from prospective cohort studies (*n* = 73), case–control studies (*n* = 7), and randomized controlled trials (*n* = 1). The studies took place in Europe (*n* = 47), Asia (*n* = 16), North America (*n* = 11), Latin America (*n* = 4), South America (*n* = 3), and Africa (*n* = 2). Sample sizes ranged from 17 to 62,941 participants. Sex of the children assessed varied across studies, from 20% to 86% female, and age range varied from birth to 17 y. Participant characteristics are provided in Table 2.

**TABLE 2:** Study and participant characteristics in included studies.

Author [reference]	Study characteristics	Participant characteristics						
	Study design, study name, country, sample size, exposure timing	Maternal age (y)	Maternal anthropometrics	Child age; Child sex (% female)	Child anthropometrics	Race	Socioeconomic status (e.g. income, education)	Infant feeding practices
Ballester et al., 2018 [21]	PCS, INMA, Spain, *N* = 1867, Pregnancy	Mean (range): 31.0 (16.0–43.0)	Mean (SD): Prepregnancy BMI (kg/m^2^): 23.5 (4.2)	Newborn; 47.6%	NR	Spain: 92.2%; Other: 7.8%	Maternal education: Up to primary: 24.1% Secondary: 40.7% University: 35.2% Social class: I, II (higher): 31.9% III: 25.2% IV, V (lower): 42.9%	NR
Barbone et al., 2020 [22]	PCS, NR, Italy, *N* = 53, Pregnancy	NR	NR	Mean (SD): 25.1 (3.7) mo; 47.2%	Weight (g): Fine motor-adaptive area > age: 3444.1 Fine motor-adaptive area ≤ age: 3354.6	NR	NR	Mean (SD): Breastfeeding: 7.9 (6.4) mo
Beck et al., 2023 [23]	PCS, Odense Child Cohort, Denmark, *N* = 999, Pregnancy and childhood	*n* (%): <28: 251 (25) 28–34: 496 (50) >34: 252 (25)	*n* (%): BMI (kg/m^2^): <25: 634 (64) 25–30: 261 (26) >30: 104 (10)	18–36 mo; 46.0%	NR	NR	*n* (%): Education level: High school or less: 258 (26) High school +1–4 y: 509 (52) High school +>4 y: 218 (22) Missing: (14)	Median (IQR): breastfeeding: 7.4 (3.7–10.6) mo
Budtz-Jorgensen et al., 2007 [24]	PCS, NR, Faroe Islands, *N* = 1022, Pregnancy	NR	NR	NR; NR	NR	NR	NR	NR
Chan et al., 2021 [25]	PCS, Hong Kong Birth Cohort, China, *N* = 604, Childhood	NR	NR	Mean (SD): 8.1 (0.9) y; 46.0%	Mean (SD): BMI (kg/m^2^): 16.7 (2.7)	NR	%: Monthly family income (Hong Kong dollar): <10,000: 24.2 10,000–20,000: 42.4 20,000–30,000: 19.4 30,000–40,000: 6.8 >40,000: 7.3	NR
Cunha et al., 2018 [26]	PCS, NR, Brazil, *N* = 1373, Pregnancy and lactation	Mean (SD): Urban: 23.1 (6.1) Nonurban: 22.6 (5.9)	NR	0–59 mo; 50.3%	Mean (SD): Urban weight at birth (g): 3281 (498.0) Urban length at birth (cm): 51.0 (2.66) Nonurban weight at birth (g): 3150 (422.1) Nonurban length at birth (cm): 50.5 (2.57)	NR	NR	Mean (SD): Breastfeeding: Urban: 5.6 (5.6) mo Nonurban: 7.4 (5.8) mo
Da Cunha et al., 2013 [27]	PCS, NR, Brazil, *N* = 18, Lactation	Mean (SD): 27.3 (5.0)	Mean (SD): Weight, postgestation (kg): 80.4 (8.8) Weight gain during gestation (kg): 15.3 (4.8)	0–90 d; 50.0%	Mean (SD): Weight at birth: (kg): 3.3 (0.4) Weight gain (0–90 d) (kg): 2.5 (0.7)	NR	NR	Mean (SD): Breastfeed volume (mL): 30 d (*n* = 17): 935 (203) 60 d (*n* = 15): 1103 (171) 90 d (*n* = 13): 1261 (73)
Davidson et al., 2008 [28]	PCS, SCDS, Republic of Seychelles, *N* = 229, Pregnancy	Range: enrollment age: 16–43	NR	0–30 mo; 50.7	NR	NR	NR	NR
Deroma et al., 2013 [29]	PCS, Friuli Venezia Giulia Cohort, Italy, *N* = 154, Pregnancy	Mean (SD): Age at follow-up: 39.4 (5.1)	NR	Mean (SD): Age at follow-up: 7.7 y (0.7); 50.0%	*n* (%): Low birth weight (<2500 g): 12 (7.8)	NR	*n* (%): Maternal education at follow-up: Elementary school: 2 (1.3) Middle school: 47 (30.9) High school: 79 (52.0) University: 24 (15.8) Maternal occupation at follow-up: Employed: 108 (71.1)	Breastfeeding: 89%
Drouillet-Pinard et al., 2010 [30]	PCS, EDEN Mother–Child Cohort, France, *N* = 645, Pregnancy	28.7	Mean: Prepregnancy BMI (kg/m^2^): 23	NR; 45.4%	Mean Birth weight (g): 3280	NR	NR	NR
Emeny et al., 2019 [31]	PCS, New Hampshire Birth Cohort Study, United States, *N* = 1321, Pregnancy	Mean (range): At enrollment: 31.4 (18.4–46.2)	Mean (range): Prepregnancy BMI (kg/m^2^): 25.9 (16.6–51.5)	0–12 mo; 49.0%	Median (range): Birth weight (g): 3453 (1080–5400)	*n* (%): White: 1318 (100) Other: 3 (0)	*n* (%): Education: Less than 11th grade: 14 (1) High school graduate, GED: 137 (10) Junior college, some college, technical school: 251 (19) College graduate: 514 (39) Postgraduate school: 396 (30) Relationship status: Single: 137 (10) Married: 1139 (86) Separated, divorced: 36 (3)	*n* (%): Breast milk, formula, both: 4 mo: 511 (39), 43 (3), 453 (34) 8 mo: 371 (28), 43 (3), 633 (48) 12 mo: 255 (17), 53 (4), 769 (58)
Fabelova et al., 2023 [32]	PCS, European Human Biomonitoring Initiative, Group 1: France, Spain, Norway; Group 2: Belgium, Slovakia *N* = 6837, Pregnancy	Mean (SD): Newborns= study population, (*n* = 940) At birth: 30.0 (4.3) Pregnant women study population (*n* = 5897) At delivery: 30.2 (4.5) At sampling: 30.1 (4.5)	Mean: Prepregnancy BMI (>25 kg/m^2^) Newborns study population: 26.5 Pregnant women study population: 33.6	Newborns; 47.9%	NR	NR	%: Newborns study population: Maternal education: Low (ISCED 0–2): 12.0 Medium (ISCED 3–4): 36.6 High (ISCED ≥5): 51.4 Paternal education: Low (ISCED 0–2): 20.3 Medium (ISCED 3–4): 41.9 High (ISCED ≥5): 37.8 Pregnant women study population: Maternal education: Low (ISCED 0–2): 10.9 Medium (ISCED 3–4): 30.8 High (ISCED ≥5): 58.3	NR
Fruh et al., 2021 [33]	PCS, Project Viva, United States, *N* = 1009, Pregnancy	Mean (SD): 32.4 (5.0)	NR	Mean (SD): During assessment: 7.8 (0.8); 49.3%	NR	%: Asian: 3.2 Black: 13.5 Hispanic: 3.3 White: 69.4 Other: 10.7	%: Maternal college graduate: 72.0 Paternal college graduate: 66.6 Household annual income (United States dollar): ≤40,000: 14.8 40–70,000: 21.9 >70,000: 63.3	NR
Garcia-Esquinas et al., 2013 [34]	PCS, BioMadrid Project, Spain, *N* = 112, Pregnancy	N: ≤30 Lead: 54 Mercury: 50 Cadmium: 54 >30 Lead: 58 Mercury: 56 Cadmium: 58	NR	Newborns; NR	Mean: Weight at birth: 3282 (g) Birth length (cm): 49.8	N: Lead: Caucasian: 85 Other: 27 Mercury: Caucasian: 78 Other: 28 Cadmium: Caucasian: 85 Other: 27	N: < High school: Lead: 32 Mercury: 43 Cadmium: 37 High School: Lead: 31 Mercury: 39 Cadmium: 36 >High school: Lead: 32 Mercury: 43 Cadmium: 37	NR
Geer et al., 2012 [35]	PCS, University Hospital of Brooklyn’s Prenatal Clinic, United States, Caribbean/West Indies, the African continent, Latin America, Europe, and Canada, *N* = 190, Pregnancy	*n* (%): ≤24: 69 (36.3) 25–29: 50 (26.3) 30–34: 44 (23.2) ≥35: 27 (14.2)	NR	Newborns; NR	NR	*n* (%): African American: 81 (42.6) Caribbean or West Indian: 78 (41.1) African, from the Africa continent: 12 (6.3) Latino/Hispanic: 15 (7.9) Other: 4 (2.1)	*n* (%): Years of education: Some high school or less: 48 (25.3) High school degree: 59 (31.0) Technical school, some college or more: 83 (43.7)	NR
Gennings et al., 2020 [36]	PCS, SELMA, Sweden, *N* = 1312, Pregnancy	NR	Mean (SD): Weight (kg): At birth: 69.0 (13) 7 y: 69.0 (13)	0–7 y; At birth: 48.0: 7 y: 50.0%	Mean (SD): Birth weight (g): 3630.6 (547)	NR	%: Maternal higher education: At birth: 63 7 y: 69	NR
Golding et al., 2016 [37]	PCS, ALSPAC, United Kingdom, *N* = 2875–3264, Pregnancy	N: <20: 239 20–24: 813 25–29: 1531 30–34: 1019 35+: 311	NR	6–42 mo; NR	NR	NR	N: Maternal education: A (lowest): 673 B: 335 C: 1155 D: 802 E (highest): 547	NR
Golding et al., 2017 [38]	PCS, ALSPAC, United Kingdom, *N* = 4285, Pregnancy	*n* (%): <20: 240 (6.1) ≥20–24: 719 (18.2) ≥25–29: 1537 (38.9) ≥30–34: 1105 (28.0) ≥35: 346 (8.8)	NR	8 y; NR	NR	*n* (%): White: 3585 (97.6) Black (African, Caribbean, other): 42 (1.1) Indian, Pakistani, Bangladeshi: 23 (0.6) Other: 22 (0.6)	*n* (%): Maternal education: None/CSE: 709 (19.2) Vocational: 345 (9.4) O level: 1226 (33.3) A level: 841 (22.8) Degree: 566 (15.4) Maternal social class: I: 200 (6.6) II: 960 (31.7) III (non-manual): 1276 (42.2) III (manual): 228 (7.5) IV: 360 (9.7) V: 67 (2.2)	—
Golding et al., 2018 [39]	PCS, ALSPAC, United Kingdom, *N* = 3840, Pregnancy	NR	NR	6 mo–11 y; NR	NR	NR	NR	NR
Goudarzi et al., 2017 [40]	PCS, Hokkaido Study on the Environment and Children’s Health, Japan, *N* = 185, Pregnancy	Mean (SD): 29.7 (4.7)	Mean (SD): Prepregnancy BMI (kg/m^2^): 21.0 (2.9)	NR; 56.2%	Mean (SD): Birth weight (g): 3130.4 (331.6)	Participants from Japan	*n* (%): Maternal education: ≤12 y: 86 (46.5) ≥13 y: 99 (53.5) Annual household income (million yen): <5: 129 (70.5) ≥5: 54 (29.5)	NR
Grandjean et al., 2001 [41]	PCS, Faroe Islands Birth Cohort Study, Denmark, *N* = 182 mother–child pairs, Pregnancy	Mean (SD): 28.0 (5.8)	Mean (SD): Prepregnancy weight (kg): 62.0 (10.5)	Newborns; 48.9%	Mean (SD): Birth weight (g): Boys: 3801 (469) Girls: 3537 (463)	NR	NR	NR
Gregory et al., 2016 [42]	PCS, ALSPAC, United Kingdom, *N* = 2207–2209, Pregnancy	NR	NR	During follow ups: 7–17 y; NR	NR	NR	NR	NR
Halldorsson et al., 2008 [43]	PCS, Danish National Birth Cohort, Denmark, *N* = 100, Pregnancy	Median (IQR): At recruitment: 29 (25–35)	Median (IQR): Prepregnancy BMI (kg/m^2^): 21.3 (18.5–25.0)	NR; 47.0%	Mean (SD): Birth weight (g): 3580 (435)	NR	%: Socioeconomic status: High: 45 Intermediate: 19 Worker: 12 Not working: 24	NR
Halldorsson et al., 2009 [44]	PCS, Danish National Birth Cohort, Denmark, *N* = 100, Pregnancy	Median (range): At recruitment: 28 (25–35)	Median (range): Prepregnancy BMI (kg/m^2^): 21.8 (18.5–24.9)	NR; NR	Mean: Birth weight (g): 3560	Participants from Denmark	%: Socioeconomic status: High: 47 Intermediate: 17 Worker: 15 Students: (21)	Mean: Breastfeeding: 7.6 mo
Han et al., 2018 [45]	PCS, Laizhou Wan Birth Cohort, China, *N* = 369 families, Pregnancy	Mean (SD): 28.35 (4.06)	Mean (SD): Prepregnancy BMI (kg/m^2^): 21.99 (3.45)	Newborns; 48.8%	NR	NR	*n* (%): Maternal education level: <High school degree: 158 (42.8) High school degree: 111 (30.1) >High school degree: 100 (27.1) Paternal education level: <High school degree: 108 (29.3) High school degree: 136 (36.8) >High school degree: 125 (33.9) Household monthly salary (Chinese yuan): <3000 ($483.6): 198 (53.6) 3000–5000 ($483.6–$806.0): 125 (33.9) >5000 ($806.0): 46 (12.5)	NR
Hertz-Picciotto et al., 2010 [46]	CC, CHARGE study, United States, *N* = 452, Childhood	%: ≥35 at delivery: 24.0	NR	%: 2 y: 28 3 y: 39 4 y: 33; 20.0%	NR	%: Maternal birthplace: United States: 74 Mexico: 13 Neither: 13	%: Maternal education at delivery: <12 y: 10 ≥16 y: 40	NR
Hibbeln et al., 2018 [47]	PCS, Avon Longitudinal Study of Parents and Children, United Kingdom, *N* = 2224, Pregnancy	N: <20: 239 20–24: 813 25–29: 1531 30–34: 1019 35+: 311	NR	During follow ups: 7–13 y; NR	NR	Participants from Avon area, United Kingdom	N: Maternal education: A (lowest): 673 B: 335 C: 1155 D: 802 E: (highest) 547 Housing tenure: Owned/mortgaged: 2695 Council rented (public housing): 570 Other: 444	NR
Hu et al., 2016 [48]	PCS, Laizhou Wan Birth Cohort, China, *N* = 410, Pregnancy	*n* (%): ≤25: 130 (31.7) 25–30: 160 (39.0) >30: 120 (29.3)	*n* (%): Prepregnancy BMI (kg/m^2^): ≤18.5: 40 (9.8) 18.5–23: 254 (62.0) >23: 116 (28.3) Pregnancy weight gain (kg): ≤10: 83 (20.2) 10–20: 219 (53.4) >20: 108 (26.3)	1 y; 47.8%	Mean (SD): Birth weight (g): 3422.40 (89.98) Body length (cm): 50.82 (3.33) Head circumference (cm): 33.34 (1.93)	NR	*n* (%): Education: ≤High school: 185 (45.1) >High school: 225 (54.9) Household monthly income (Chinese yuan): ≤3000: 260 (63.4) 3000–5000: 118 (28.8) >5000: 32 (7.8)	NR
Jedrychowski et al., 2007 [49]	PCS, Krakow Epidemiology Study, Poland, *N* = 374, Pregnancy	Mean (SD): 27.68 (3.39)	Mean (SD): Before pregnancy: Height (cm): 164.97 (5.274) Weight (kg): mean: 58.23 (8.512)	12–36 mo; 48.9%	Mean (SD): Birth weight (g): 3440.2 (452.8) Birth length (cm): 54.79 (2.64) Head circumference (cm): 33.89 (1.44)	Newborns from Krakow, Poland	*n* (%): Education: Primary school only: 31 (8.3) Medium or high school: 88 (23.5) University degree or uncompleted university: 255 (68.2)	NR
Jeong et al., 2017 [50]	PCS, Mothers and Children’s Environmental Health, Korea, *N* = 553, Pregnancy	*n*: <30: 243 ≥30: 305	NR	60 mo; 46.8%	*n*: Birth weight (g): First tertile: 184 Second tertile: 184 Third tertile: 185	NR	*n*: Maternal education level: ≤12 y: 132 >12 y: 404 Paternal education level: ≤12 y: 128 >12 y: 393 Household income, United States Dollars/mo: <2000: 139 ≥2000: 395	NR
Julvez et al., 2016 [51]	PCS, INMA, Spain, *N* = 1499, Pregnancy	*n*: <31: 946 ≥31: 1049	NR	14 mo–5 y; 49.0%	NR	*n*: Born in: Spain: 1858 Latin America: 88 Other places: 46	*n*: Education: ≤Primary: 424 Secondary: 829 University: 738 Social class: High Skilled: 815 Non-manual: 730 Manual: 423	*n*: Breastfeeding: ≤24 wk: 1078 >24 wk: 872
Kim et al., 2016 [52]	PCS, CHECK, Korea, *N* = 302, Pregnancy	Mean (SD): 33.3 (3.9)	Mean (SD): BMI (kg/m^2^): 21.5 (3.3)	Newborns; NR	NR	NR	*n* (%): Income (≥$3000/mo): 184 (70.8)	NR
Kim et al., 2018 [53]	PCS, Mothers and Children’s Environmental Health Study, Republic of Korea, *N* = 1751, Pregnancy	*n* (%):6 mo of child: ≤35: 1005 (91.5) >35: 93 (8.5)	NR	6–36 mo; 48.0%	*n* (%):Birth weight group (kg): <32: 446 (40.6)≥32: 652 (59.4)	NR	*n* (%):Maternal education: College: 430 (39.2) >University: 551 (50.2) Income: <$2000: 276 (25.1) $2000–$3000: 374 (34.1) >$3000: 390 (35.5)	*n* (%): Breastfeeding: Never: 43 (3.9) <1 mo: 494 (45.0) 1–12 mo: 296 (27.0) >12 mo: 265 (24.1)
Kindgren et al., 2019 [54]	CC, All Babies in Southeast Sweden Project, Sweden, *N* = 42 patients; *N* = 40 age-matched and sex-matched controls, Childhood	NR	NR	NR; NR	NR	NR	NR	NR
Kvestad et al., 2018 [55]	RCT, FINS-KIDS, Norway, *N* = 232, Childhood	NR	NR	0–16 y; NR	NR	NR	NR	NR
Llop et al., 2012 [56]	PCS, INMA, Spain, *N* = 1683, Pregnancy	%: At birth: 30–34 y: 44.3	*n* (%): Prepregnancy BMI (kg/m^2^): Healthy (18.5–<25): 1180 (70.1) Underweight (<18.5): 67 (4.0) Overweight (25–<30): 320 (19.0) Obese (≥30): 116 (6.9)	2 y; 47.6%	*n* (%): Low birth weight: No: 1619 (96.4) Yes: 61 (3.6) Small-for-gestational-age length: No: 1492 (90.6) Yes: 154 (9.4)	*n* (%): Country of birth Spain: 1545 (92.2) Other: 131 (7.8)	*n* (%): Up to primary: 372 (22.2) Secondary: 690 (41.1) University: 617 (36.7)	*n* (%): Breastfeeding: 0 wk: 241 (14.7) >0–16 wk: 392 (23.8) >16–24 wk: 254 (15.5) >24: 757 (46.0)
Llop et al., 2017 [57]	PCS, INMA, Spain, *N* = 1362, Pregnancy	*n* (%): <25: 61 (4.5) 25–29: 416 (30.5) 30–34: 606 (44.5) 35: 278 (20.4)	*n* (%): BMI before pregnancy (kg/m^2^): Low weight: 54 (4.0) Healthy weight: 950 (69.8) Overweight: 259 (19.0) Obesity: 99 (7.3)	4–5 y; 52.3%	NR	Participants from Spain	*n* (%): Maternal education level: Up to primary: 281 (20.7) Secondary: 564 (41.5) University: 514 (37.8) Paternal education level: Up to primary: 452 (33.4) Secondary: 603 (44.6) University: 298 (22.0) Maternal working status: Nonworker: 360 (27.2) Worker: 964 (72.8) Paternal working status: Nonworker: 121 (9.2) Worker: 1191 (90.8) Social class: SC I+II (high): 475 (34.9) SC III: 350 (25.7) SC IV+V (low): 536 (39.4)	*n* (%): Breastfeeding 0 wk: 185 (13.8) >0–16 wk: 309 (23.1) >16–24 wk: 215 (16.1) >24 wk: 627 (46.9)
Llop et al., 2020 [58]	PCS, INMA, Spain, *N* = 1252, Childhood	*n* (%): <25: 70 (6) 25–29: 411 (33) 30–34: 544 (43) ≥35: 226 (18)	*n* (%): Prepregnancy BMI (kg/m^2^): <18.5: 55 (4) 18.5–25: 884 (71) >25–30: 221 (18) >30: 92 (7)	Range: 4.1–6.4 y; 53.0%	NR	*n* (%): Maternal country of birth: Spain: 1163 (93) Other: 86 (7)	*n* (%): Maternal education level: Up to primary: 281 (22) Secondary: 519 (42) University: 449 (36) Paternal education level: Up to primary: 436 (35) Secondary: 534 (43) University: 273 (22) Social class: I +II: 441 (35) III: 334 (27) IV +V: 477 (38)	NR
Lozano et al., 2021 [59]	PCS, INMA, Spain, *N* = 472, Childhood	Mean (SD): 30.5 (4.1)	Mean (SD): Prepregnancy BMI (kg/m^2^): 23.5 (4.3)	9 y; 50.6%	NR	*n* (%): Country of birth: Spain: 381 (94.5) Other: 22 (5.5)	*n* (%): Parental social class: I + II (higher): 114 (28.3) III: 108 (126.8) IV +V (lower): 181 (44.9) Maternal educational level: Up to primary: 112 (27.8) Secondary: 172 (42.7) University: 119 (29.5) Parental educational level: Up to primary: 173 (43.1) Secondary: 156 (38.9) University: 72 (18.0)	*n* (%) Breastfeeding, 0 wk: 63 (15.6) >0–16 wk: 90 (22.3) >16–24 wk: 65 (16.1) >24 wk: 185 (45.9)
Marques et al., 2016 [60]	PCS, Western Amazon, Brazil, group 1: *n* = 258; group 2: *n* = 288; group 3: *n* = 144, Lactation	Mean (SD): Group 1: 22.8 (6.3) Group 2: 23.4 (6.2) Group 3: 23.3 (6.1)	NR	0–24 mo; NR	Mean (SD): Birth weight (kg): Group 1: 3.25 (0.50) Group 2: 3.26 (0.42) Group 3: 3.25 (0.41)	NR	Mean (SD): Education (y): Group 1: 5.5 (3.1) Group 2: 6.2 (3.1) Group 3: 6.2 (3.1)	Mean (SD): Breastfeeding: Group 1: 6 (0) mo Group 2: 9.8 (1.7) mo Group 3: 19.4 (3.7) mo
Mendez et al., 2010 [61]	PCS, INMA, Spain, *N* = 592, Pregnancy	Mean (SD): 31.5 (4.4)	Mean (SD): BMI (kg/m^2^): 23.7 (4.5)	NR; NR	Mean (SD): Birth weight (g): 3269 (400)	NR	%: Secondary education: 28.3 Manual worker: Mother: 21.2 Father: 55.6	NA
Miklavcic et al., 2013 [62]	PCS, NR, Italy, Slovenia, Croatia, Greece, *N* = 2202, Lactation	*n*: Italian women: 18–20: 2 20–30: 118 30–40: 558 >40: 72 Slovenian women: 20–30: 147 30–40: 209 >40: 12 Croatian women: 20–30: 81 30–40: 104 >40: 3 Greek women: 20–30: 157 30–40: 173 >40: 11	NR	Newborns; NR	NR	*n*: Italian: 900 Slovenian: 584 Croatian: 234 Greek:484	NR	NR
Miyashita et al., 2015 [63]	PCS, Hokkaido Study on Environment and Children’s Health, Japan, *N* = 367, Pregnancy	Mean (SD): At birth: 30.8 (4.8)	Mean (SD): Prepregnancy weight (kg): 52.5 (8.0)	NR; 52.9%	Mean (SD): Birth weight (g): 3073 (37) Birth length (cm): 48.1 (1.9) Chest circumference (cm): 31.5 (1.6) Head circumference (cm): 33.3 (1.3)	NR	*n* (%): Annual household income (million yen): <3: 61 (16.6) 3 to <5: 183 (49.9) 5 to <7: 78 (21.3) ≥7: 45 (12.3) Education level: ≤9 y: 7 (1.9) 10–12 y: 147 (40.1) 13–16 y: 208 (56.7) ≥17 y: 5 (1.4)	NR
Morrissette et al., 2004 [64]	PCS, NR, Canada, *N* = 159, Pregnancy	Mean (SD): 26.7 (5)	Mean (SD): Weight (kg): 66.4 (16.0)	NR; 48.0%	Mean (SD): Birth weight (kg): 3.3 (0.5) Birth length (cm): 50.6 (2.9) Head circumference (cm): 34.4 (1.5)	NR	Mean (SD): Education: 12.3 (2.6) y %: Income (<25,000 United States dollars): 29.7	NR
Muniroh et al., 2022 [65]	PCS, NR, Indonesia, *N* = 118, Pregnancy	Median (range): 29.5 (19–39)	Median (range): Prepregnancy BMI (kg/m^2^): 22.8 (14.4–41.1)	Newborns; 44.9%	Medians (range): Birth weight (g): 3100 (2260–4200) Birth length (cm): 49 (44–59)	Participants from Indonesia	*n* (%): Education: Elementary-junior: 38 (32.2) High school: 70 (59.3) Universities: 10 (8.5) Occupation: Laborer: 5 (4.2) Employees: 28 (23.7) Entrepreneur: 8 (6.8) Housewife: 77 (65.3)	NR
Nakamura et al., 2008 [66]	PCS, Tohoku Study of Child Development, Japan, *N* = 49, Pregnancy and Lactation	Mean (SD): At delivery: 32.4 (4.7)	Mean (SD): Prepregnancy BMI (kg/m^2^): 21.4 (2.5) Predelivery BMI (kg/m^2^): 25.3 (2.7)	Newborns; 61.0%	Mean (SD): Weight (g): 3143 (359)	Participants from Japan	*n* (%): Maternal education: 12 y or less: 6 (12) More than 13 y: 43 (88) Paternal education: 12 y or less: 19 (39) More than 13 y: 30 (61)	NR
Nisevic et al., 2019 [67]	PCS, NR, Croatia, Italy, *N* = 257 mother–infant pairs, Pregnancy	Median (range): 33 (24–40)	NR	18 mo; 48.2%	NR	Participants from Croatia and Italy	NR	NR
Oken et al., 2005 [68]	PCS, Project Viva, United States, *N* = 135 mother–infant pairs, Pregnancy and lactation	%: <30: 16 30–34: 53 ≥35: 31	NR	%: During testing (mo): <7: 80 ≥7: 20; 51.0%	%: Birth weight for gestational age: Small (<10th percentile): 2 Appropriate: 85 Large (>90th percentile): 13	(%): White: 82 Non-White: 18	%: Education level: College or graduate degree: 80 <College graduate: 20 Marital status: Married or cohabitating: 92 Divorced or single: 8	%: Breastfeeding: <2 mo: 19 2–4 mo: 23 ≥5 mo: 58
Oken et al., 2008 [69]	PCS, Project Viva, United States, *N* = 341, Pregnancy and lactation	Mean (SD): 32.6 (4.7)	*n* (%): Prepregnancy BMI (kg/m^2^): <25: 70 25 to <30: 20 ≥30: 10	Mean (SD): During testing (mo): 38.4 (2.2); 51.0%	NR	%: African American: 6 Hispanic: 2 Other: 9 Caucasians: 82	%: Maternal education: High school: 6 Some college: 14 College graduate: 40 Graduate degree: 41 Paternal education: High school: 23 College graduate: 41 Graduate degree: 36	Mean (SD): Breastfeeding: 7.0 (4.5) mo
Oken et al., 2016 [70]	PCS, Project Viva, United States, Participants with data on mid-pregnancy diet: *n* = 1068; Participants with data on mid-pregnancy blood: *n* = 872, Pregnancy	Mean (SD, range): At enrollment: Participants with data on mid-pregnancy diet: 32.2 (5.3, 15.3–44.9) Participants with data on mid-pregnancy blood: 32.4 (5.1, 15.3–44.9)	Mean (SD, range): Prepregnancy BMI (kg/m^2^): Participants with data on mid-pregnancy diet: 24.6 (5.1, 16.2–48.5) Participants with data on mid-pregnancy blood: 24.5 (5.0, 16.2–48.5)	Mean (SD, range): Participants with data on mid-pregnancy diet: 7.9 (0.8, 6.6–10.9) Participants with data on mid-pregnancy blood: 7.9 (0.8, 6.6–10.7); Participants with data on mid-pregnancy diet: 50.0%, Participants with data on mid-pregnancy blood: 49.0%	NR	%: Maternal: Participants with data on mid-pregnancy diet: Asian: 5 Black: 16 Hispanic: 6 White: 69 Other: 4 Participants with data on mid-pregnancy blood: Asian: 5 Black: 14 Hispanic: 5 White: 72 Other: 4	%: College graduate: Participants with data on mid-pregnancy diet: No: 31 Yes: 69 Participants with data on mid-pregnancy blood: No: 29 Yes: 71 Annual household income (>70,000 United Stated dollar): Participants with data on mid-pregnancy diet: No: 38 Yes: 62 Participants with data on mid-pregnancy blood: No: 36 Yes: 64	Mean (SD, range): Breastfeeding: Participants with data on mid-pregnancy diet: 6.4 (4.6, 0–12.0) mo Participants with data on mid-pregnancy blood: 6.5 (4.6, 0–12.0) mo
Papadopoulou et al., 2019 [71]	PCS, HELIX, United Kingdom, France, Spain, Lithuania, Norway, Greece, *n* = 818 mothers; *n* = 1288 children, Pregnancy	NR	NR	5–12 y; NR	NR	NR	NR	NR
Papadopoulou et al., 2021 [72]	PCS, Norwegian Mother, Father and Child Cohort, Norway, *N* = 51,952 mother–child pairs, Pregnancy	Mean (SD): 30.3 (4.4)	Mean (SD): Prepregnancy BMI (kg/m^2^): 24.0 (4.2)	1 mo–8 y; NR	*n* (%): Birth weight (kg): ≤2.5: 1372 (3) 2.6–3.5: 23,295 (45) 3.6–4.4: 24,994 (48) ≥4.5: 2291 (4)	NR	*n* (%): Maternal education: Low (12 y): 14,405 (28) Average (13–16 y): 22,993 (44) High (17 y): 14,554 (28)	NR
Qin et al., 2018 [73]	CC, NR, China, *N* = 72, Childhood	NR	NR	Mean (SD): Children with ASD: Boys: 4.10 (0.81) Girls: 4.28 (1.06) Unaffected children: Boys: 4.29 (1.73) Girls: 4.35 (1.99); 43.0%	Mean: Height (cm): 95 Weight (kg): 14.4	NR	NR	%: Breastfed: 100
Rahbar et al., 2012 [74]	CC, NR, Jamaica, *N* = 130, Childhood	*n* (%): At child’s birth: >35: Case: 17 (26.2) Control: 7 (11.3)	NR	*n* (%): Case (mo): age<48: 10 (15.4) 48≤age<72: 34 (52.3) Age≥72: 21 (32.3) Control (mo): age<48: 9 (13.8) 48≤age<72: 34 (52.3) Age≥72: 22 (33.9); NR	NR	%: Afro-Caribbean: Case: 96.9 Control: 98.5	*n* (%): Maternal education: Case: Up to high school: 34 (52.3) Beyond high school: 31 (47.7) Control: Up to high school: 51 (81.0) Beyond high school: 12 (19.0) Paternal education: Case: Up to high school: 32 (50.8) Beyond high school: 31 (49.2) Control: Up to high school: 56 (91.8) Beyond high school: 5 (8.2) Own a vehicle: Case: 45 (69.2) Control: 22 (33.8)	NR
Rahbar et al., 2013 [75]	CC, Jamaican Autism Study, Jamaica, *n* = 65 age-matched pairs *N* = 130 total, Childhood	*n* (%): At child’s birth: >35: ASD cases: 17 (26.2) Control: 7 (11.3)	NR	*n* (%): ASD cases (mo): <48: 10 (15.4) 48–<72: 34 (52.3) ≥72: 21 (32.3) Control cases (mo): <48: 9 (13.8) 48–<72:34 (52.3) ≥72: 22 (33.9); NR	NR	Participants from Jamaica	*n* (%): Parental education at delivery: At least one parent had education beyond high school: ASD cases: 42 (66.7) Control: 14 (23.7)	NR
Rahbar et al., 2014 [76]	CC, Jamaican Autism Study, Jamaica, *N* = 220, Childhood	*n* (%): At child’s birth: >35: ASD cases: 28 (25.5) Control: 11 (10.5)	NR	*n* (%): ASD cases (mo): <48: 21 (19.1) 48–<72: 49 (44.5) ≥72: 40 (36.4) control cases (mo): <48: 18 (16.4) 48–<72: 50 (45.5) ≥72: 42 (38.2); NR	NR	Participants from Jamaica	*n* (%): Maternal education at delivery: Up to high school: ASD cases: 57 (51.8) Control: 82 (76.6) Beyond high school: ASD cases: 53 (48.2) Control: 25 (23.4) Paternal education at delivery: Up to high school: ASD cases: 59 (55.1) Control: 93 (87.7) Beyond high school: ASD cases: 48 (44.9) Control: 13 (12.3)	NR
Ramon et al., 2008 [77]	PCS, INMA, Spain, *N* = 253, Pregnancy	*n* (%): <25: 29 (11.5) 25–29: 86 (34.0) 30–34: 107 (42.3) ≥35: 31 (12.3)	*n* (%): Prepregnancy BMI (kg/m^2^): Underweight (<18.5): 16 (6.4) Healthy weight (18.5≤BMI<25): 164 (65.3) Overweight (25≤BMI<30): 48 (19.1) Obese (BMI≥30): 23 (9.2)	Newborns; 46.6%	NR	*n* (%): Country of origin: Spain: 224 (88.5) Latin America: 22 (8.7) Rest of Europe: 7 (2.8)	*n* (%): Educational level: Up to primary school: 91 (36.0) Secondary school: 104 (41.1) University degree: 58 (22.9)	NR
Ramon et al., 2009 [78]	PCS, INMA, Spain, *N* = 550, Pregnancy	Mean (SD): 30.1 (4.6)	Mean (SD): Prepregnancy BMI (kg/m^2^): 23.9 (4.6)	Newborns; NR%	Mean (SD): Birth weight (g): 3273 (487) Birth length (cm): 50.3 (2.2)	%: Spanish origin: 88.7	%: High socio-occupational status: 15.6 Secondary school: 41.1 Employed at first trimester: 62.0	NR
Rothenberg et al., 2016 [79]	PCS, NR, China, *N* = 270 mother-child pairs, Pregnancy and Lactation	*n* (%): <20: 23 (8.5) 20–29: 156 (58) 30–44: 91 (34)	*n* (%): Prepregnancy BMI (kg/m^2^): Underweight: 72 (27) Normal weight: 151 (56) Overweight: 38 (14) Obese: 9 (3.3)	Newborns 53.0 0–36 mo; 49.0%	*n* (%): Birth weight for gestational age (centile) Centile<10th: 33 (12) 10th ≤Centile < 90th: 223 (83) Centile ≥ 90th: 14 (5.2)	*n* (%): Maternal: Zhuang: 235 (87) Han: 29 (11) Other: 6 (2.2)	*n* (%): Maternal education Completed: <High School: 215 (80) High School: 40 (15) University: 15 (5.6) Paternal education Completed: <High School: 209 (77) High School: 45 (17) University: 16 (5.9) Household Income (Chinese yuan): Income <2000: 180 (67) 2000 ≤ Income <5000: 77 (29) Income ≥5000: 13 (4.8)	*n* (%): Breastfeeding: <3 mo: 10 (3.7) 3–<6 mo:22 (8.2) 6–<9 mo: 101 (37) >9 mo:137 (51)
Rothenberg et al., 2021 [80]	PCS, NR, China, *N* = 391, Pregnancy and Lactation	*n* (%): At enrollment: <20: 28 (7) 20 ≤ Age < 30: 223 (57) 30 ≤ Age < 45: 140 (36)	*n* (%): Prepregnancy BMI (kg/m^2^): Underweight: 90 (23) Normal Weight: 231 (59) Overweight: 60 (15) Obese: 9 (2.3) Missing: 1 (< 1)		NR	*n* (%): Maternal: Zhuang: 333 (85) Han: 50 (13) Other: 8 (2)	*n* (%): Household Monthly Income (Chinese yuan): Income < 2000: 230 (59) 2000 ≤ Income < 5000: 107 (27) Income ≥5000: 19 (4.9) Missing: 35 (9.0) Maternal education Completed: <High School: 316 (81) High School: 47 (12) Some University: 18 (4.6) Missing: 10 (2.6) Paternal education Completed: <High School: 304 (78) High School: 57 (15) Some University: 20 (5.1) Missing: 10 (2.6) Maternal occupation: Farmer: 298 (76) Worker: 31 (7.9) Unemployed: 42 (11) Other: 13 (3.3) Missing: 7 (1.8) Paternal occupation: Farmer: 289 (74) Worker: 53 (14) Unemployed: 23 (5.9) Other: 17 (4.3) Missing: 9 (2.3)	*n* (%): Breastfeeding for more than median 8.5 mo: Follow-up at 12 mo: Yes: 127 (48) No: 136 (52) Missing: 1 (<1) Follow-up at 36 mo: Yes: 89 (47) No: 100 (53) Missing: 1 (<1)
Sagiv et al., 2012 [81]	PCS, New Bedford Cohort, United States, *N* = 607, Pregnancy	*n* (%): At child’s birth: Children with 8-y data: <20: 79 (13.1) 20–29: 317 (52.5) 30–34: 131 (21.7) ≥ 35: 77 (12.7) Children with Maternal Hair Mercury Data: <20: 56 (13.3) 20–29: 209 (49.6) 30–34: 100 (23.8) ≥ 35: 56 (13.3)	NR	8 y; 49.0%	NR	*n* (%): Child: White: 411 (69.3) Non-White: 182 (30.7) Missing: 11	*n* (%): Annual household income at child’s 8-y examination (United States dollar): <20,000: 74 (17.8) 20,000–39,999: 112 (27.0) ≥40,000: 229 (55.2) Missing: 6 Maternal educational level at child’s 8-y examination: <12th grade: 34 (8.2) High school graduate: 127 (30.5) Some college: 255 (61.3) Missing: 5 Paternal educational level at child’s 8-y examination: <12th grade: 95 (23.5) High school graduate: 176 (43.5) Some college: 134 (33.1) Missing: 16	NR
Signes-Pastor et al., 2022 [82]	PCS, INMA, Spain, *N* = 339, Childhood	Median (range): At enrollment: 39 (21–43)	NR	During follow ups: 4 and 7 y; 42.5%	NR	NR	*n* (%): Maternal education: Primary: 65 (16.3) Secondary: 141 (35.3) University: 133 (33.3)	NR
Stepanova et al., 2018 [83]	PCS, KFU, Russia, *N* = 180, Childhood	NR	NR	3–7 y; NR	NR	NR	NR	NR
Stratakis et al., 2020 [84]	PCS, HELIX, France, Greece, Norway, Spain, United Kingdom, *N* = 805, Pregnancy	Mean (SD): 31.3 (4.6)	Mean (SD): Prepregnancy BMI (kg/m^2^): 24.0 (4.4)	Mean (SD): Age at assessment: 8.4 (1.5) y; 43.7%	Mean (SD): Birth weight (g): 3347 (488)	%: White: 91.2% Asian: 6.8% Other: 2.0%	%: Maternal education level: Low: 13.2 Medium: 35.2 High: 49.7 Missing: 2.0	NR
Tatsuta et al., 2017 [85]	PCS, Tohoku Study of Child Development Cohort, Japan, *N* = 566, Pregnancy and Lactation	NR	NR	Range: 17–20 mo; 49.6%	Mean (SD): Birth weight (g): Boys: 3180 (376) Girls: 3108 (333)	NR	*n* (%): Family income: Less than 3,000,000 Japanese Yen/y: Boys: 110 (38.6) Girls: 108 (38.4) Less than 6,000,000 Japanese Yen/y: Boys: 112 (39.3) Girls: 112 (39.9) 6,000,000 Japanese Yen/y and over: Boys: 63 (22.1) Girls: 61 (21.7)	NR
Taylor et al., 2016 [86]	PCS, ALSPAC, United Kingdom, *N* = 4044, Pregnancy	Mean (SD): 28 (5)	Mean (SD): BMI (kg/m^2^): 23 (4)	Newborns; NR	NR	NR	NR	NR
Trdin et al., 2020 [87]	PCS, PHIME Cohort, Croatia, *n* = 223 mothers *n* = 213 newborns, Pregnancy and Lactation	Mean (SD, range): 30.1 (4.8, 19–44)	Mean (SD): BMI (kg/m^2^): 23.0 (4.2)	0–1 mo; 50.2%	Mean (SD): Birth weight (kg): 3.52 (0.5)	NR	*n* (%): Education: Primary: 4 (2.1) Secondary or more: 191 (97.9) Employment: Yes: 174 (91.6) No: 18 (9.4)	NR
Vahter et al., 2000 [88]	PCS, NR, Sweden, *N* = 237, Pregnancy and Lactation	Median (range): 31 (20–45)	NR	Newborns; NR	NR	NR	NR	Median (range): Breastfeeding: 6.4 mo (0.5–12)
Valent et al., 2013 [89]	PCS, Mediterranean coastal area cohort, Italy, *N* = 606, Pregnancy and lactation	Mean (SD): At birth: 33.3 (4.3)	Mean (SD): Prepregnancy BMI (kg/m^2^): 23.3 (14.5)	18 mo; 49.3%	Mean (SD): Birth weight (g): 3419 (453)	N: Italian: 563 Other: 43	*n* (%): Maternal education: Elementary school: 5 (0.8) Middle school: 95 (15.7) High school: 293 (48.3) University degree: 211 (34.8) Not reported: 2 (0.3) Maternal occupation status: Employed: 508 (83.8) Housewife: 48 (7.9) Seeking work: 27 (4.4) Student: 6 (1.0) Stopped working: 8 (1.3) Other/missing: 9 (1.5)	NR
Varsi et al., 2022 [90]	PCS, NR, Norway, *N* = 114, Pregnancy and Lactation	Mean (SD): 31.5 (4.3)	Mean (SD): Prepregnancy BMI (kg/m^2^): 22.8 (3.1)	6 mo; 47.0%	Mean (SD): Birth weight (g): 3573 (418) Weight at 6 mo (g): 7969 (987)	Participants from Norway	*n* (%): Education: ≥12 y: 67 (59)	Mean (SD): Breastfeeding: 3.8 (1.5) mo *n* (%): Breastfed at 6 mo: 98 (86%)
Vejrup et al., 2014 [91]	PCS, Norwegian Mother and Child Cohort Study, Norway, *N* = 62,941, Pregnancy	*n* (%): At birth: <25: 7206 (11.4) 25–29: 21,368 (33.9) 30–34: 26,855 (42.7) ≥35: 7512 (11.9)	*n* (%): Prepregnancy BMI (kg/m^2^): <18.5: 1838 (2.9) 18.5–24.9: 41,333 (65.7) 25.0–29.9: 13,624 (21.6) 30.0–34.9: 4277 (6.8) ≥35.0: 1605 (2.6) Missing: 264 (0.4)	NR; NR	NR	NR	*n* (%): Maternal education: ≤12 y: 19,661 (31.2) 13–16 y: 26,333 (41.8) ≥17 y: 15,612 (24.8)	NR
Vejrup et al., 2016 [92]	PCS, Norwegian Mother and Child Cohort Study, Norway, *N* = 46,750, Pregnancy	*n* (%): <25: 4067 (8.7) 25–29: 15,583 (33.3) 30–34: 21,237 (45.4) ≥35: 5863 (12.5)	*n* (%): Prepregnancy BMI (kg/m^2^): 18.5–24.9: 31,323 (67.0) <18.5: 1284 (2.7) 25–29.9: 10,058 (21.5) 30+: 4085 (8.7)	36 mo; NR	NR	Participants from Norway	*n* (%): Maternal education (y): <12: 12,055 (25.8) 13–16: 20,661 (44.2) ≥17 13,147 (28.1) Missing: 887 (1.9) Paternal education (y): <12: 18,820 (40.3) 13–16: 13,403 (28.7) ≥17: 11,933 (25.5) Missing: 2594 (5.5)	*n* (%): Breastfeeding at 6 mo: No: 7873 (16.8) Yes: 37,596 (80.4) Missing: 1281 (2.7)
Vejrup et al., 2018 [93]	PCS, Norwegian Mother and Child Cohort Study, Norway, *N* = 38,581, Pregnancy	Mean (SD): At birth: Total population: 30.7 (4.4) Subsample: 30.5 (4.2)	Mean (SD): Prepregnant BMI (kg/m^2^): Total population: 23.9 (4.1) Subsample: 23.9 (3.9)	5 y; Total population: 49.0%, Subsample: 47.0%	NR	NR	%: Maternal education: Total population: High school or less: 23.0 College 1–3 y: 44.0 Master’s degree or higher: 31.2 Other/missing: 1.8 Subsample: High school or less: 23.0 College 1–3 y: 48.0 Master’s degree or higher: 26.8 Other/missing: 2.1 Household income >600,000 (Norwegian Krone) Total population: Yes: 35.9 No: 60.6 Missing: 0.6 Subsample: Yes: 30.8 No: 66.1 Missing: 0.5	Mean (SD): Breastfeeding: Total population: 10.2 (4.4) mo Subsample: 10.2 (4.2) mo N: Participants with data on breastfeeding: 32,441.
Vejrup et al., 2022 [94]	PCS, Norwegian Mother, Father and Child Cohort, Norway, *N* = 51,238, Pregnancy	Mean: At birth: 30	Mean: Prepregnancy BMI (kg/m^2^): 23.8	3 and 5 y; NR	NR	NR	%: At least one or more y of college/university: 72.0	NR
Vizcaino et al., 2014 [95]	PCS, INMA, Spain, *N* = 325, Pregnancy	*n* (%): <30: 105 (32.2) 30–34: 136 (41.7) ≥35: 85 (26.1)	*n* (%): Prepregnancy BMI (kg/m^2^): Underweight (< 18.5): 11 (3.4) Normal weight (18.5–25): 222, (68.3) Overweight (25–30): 71 (21.9) Obese (> 30): 21 (6.5) Gestational weight gain (kg): Inadequate: 81 (25) Recommended: 108 (33.3) Excessive: 135 (41.7)	Newborns; NR	NR	NR	*n* (%): Education: Up to primary: 54 (16.6) Secondary: 141 (43.3) University: 131 (40.2) Socioeconomic status: I + II (highest): 75 (23.2) III: 72 (22.2) IV + V (lowest): 177 (54.6)	*n* (%): Breastfeeding (previous pregnancies): Never: 231 (71.1) <16 wk: 47 (14.4) ≥16 wk: 48 (14.7)
Wang et al., 2019 [96]	PCS, NR, China, *N* = 286 mother–child pairs, Pregnancy	Mean (SD): At birth: 27.50 (3.87)	NR	18 mo; 49.3%	Mean (SD): Birth weight (g): 3392.8 (398.3)	Participants from China	*n* (%): Maternal education level: ≤9 y: 40 (14.0) ∼12 y: 52 (18.2) ∼16 y: 173 (60.5) >16 y: 21 (7.3) Monthly household income per capita (Chinese yuan): <2000: 78 (27.3) 2000–5000: 114 (39.9) >5000: 94 (32.9)	NR
Warembourg et al., 2019 [97]	PCS, HELIX, United Kingdom, France, Spain, Lithuania, Norway, Greece, *N* = 1277, Pregnancy and childhood	Mean (SD): 31.0 (4.9)	Mean (SD): Prepregnancy BMI (kg/m^2^): 24.9 (5.0)	Mean (SD): At assessment: 8.0 (1.6) y; 45.4%	Mean (SD): BMI (kg/m^2^): 16.9 (2.5)	*n* (%): United Kingdom: 202 (15.8) France: 198 (15.5) Spain: 221 (17.3) Lithuania: 203 (15.9) Norway: 254 (19.9) Greece: 199 (15.6)	*n* (%): Maternal education level: Low: 170 (13.7) Middle: 428 (34.6) High: 639 (51.7) Paternal education level: Low: 207 (17.4) Middle: 470 (39.4) High: 515 (43.2)	NR
Wohlfahrt-Veje et al., 2014 [98]	PCS, Copenhagen Mother–Child Cohort of Growth and Reproduction, Denmark, *N* = 417, Lactation	*n* (%): At birth: <25: 25 (6.0) 25–30: 164 (39.3) 30–35: 161 (38.6) >35: 67 (16.1)	*n* (%): Prepregnancy BMI (kg/m^2^): <20: 64 (16.6) 20–25: 243 (63.3) >25: 77 (20.0)	0–36 mo; 47.7%	%: Height 0–18 mo (change in SDS): <-0.67: 84 (22.3) ≥-0.67, <0.67: 181 (48.0) ≥0.67: 112 (29.7) Weight 0–18 mo (change in SDS): <-0.67: 88 (23.5) ≥-0.67, 0.67: 176 (47.1) ≥0.67: 110 (29.4)	%: Caucasian: 100	*n* (%): Social class: 1–2 (high): 267 (65.6) 3–5 (low): 140 (34.4)	*n* (%): Breastfeeding at 3 mo: No: 76 (18.3) Yes: 340 (81.7) Time of weaning: <6 mo: 80 (22.3) ≥6 mo: 279 (77.7)
Xu et al., 2016 [99]	PCS, HOME Cohort, United States, *N* = 344, Pregnancy and Lactation	Mean (SD): 30 (5.8)	NR	Mean (SD): At assessment: 34 (5) d; 53.0%	Mean (SD): Birth weight (g): 3389 (614)	*n* (%): White, non-Hispanic: 218 (63) Black, non-Hispanic: 104 (30) Other: 22 (7)	Median (IQR): Household income (United States Dollar): 55,000 (27,000–85,000) *n* (%): Education: ≤High School or GED: 74 (22) Some college or college graduate: 195 (56) Graduate or professional school: 75 (22)	*n* (%): Breastfeeding ≥1 wk: 269 (78)
Xue et al., 2007 [100]	PCS, POUCH Study, United States, *N* = 1024, Pregnancy	*n* (%): <25: 422 (41%) ≥25: 602 (59%)	NR	Newborns; NR	NR	*n* (%): White: 752 (73) African American: 183 (18) Other: 89 (9)	*n* (%): Education (y): ≤12: 430 (42) >12: 590 (58) Medicaid insured: No: 587 (57) Yes: 436 (43)	NR
Yu et al., 2022 [101]	CC, Maoming Cohort Study, China, *N* = 836, Pregnancy	Mean (SD): Preterm: 29.03 (7.04) Term: 28.14 (6.12)	Mean (SD): BMI (kg/m^2^): Term: 20.73 (3.13) Preterm: 20.96 (3.61)	Newborns Preterm: 40.3%; Term: 48.4%	NR	NR	*n* (%): Average annual family income (Chinese yuan): Preterm: <30,000: 83 (23.38) 30–100,000: 232 (65.35) ≥100,000: 40 (11.27) Term: <30,000:114 (23.70) 30–100,000: 308 (64.03) ≥100,000: 59 (12.27)	NR

Abbreviations: A level, advanced level qualification (education); ASD, Autism spectrum disorder; CC, case–control study; CSE, Certificate of Secondary Education; GED, Graduate Equivalency Degree; ISCED, International Standard Classification of Education; NR, not reported; O level, Ordinary level qualification (education); PCS, prospective cohort study.

Study cohorts—ALSPAC, Avon Longitudinal Study of Parents and Children; CHARGE, Childhood Autism Risks from Genetics and the Environment study; CHECK, Children’s Health and Environmental Chemicals in Korea; EDEN, Etude des déterminants prénatals et postnatals précoces du développement et de la santé de l’enfant (Study of early prenatal and postnatal determinants of child development and health); HELIX, Human Early Life Exposome project; HOME, Health Outcomes and Measures of the Environment; INMA, INfancia y Medio Ambiente (Environment and Childhood) Project; FINS-KIDS, Fish Intervention Studies-KIDS; KFU, Institute of Fundamental Medicine and Biology of the Kazan Federal University; POUCH, Pregnancy Outcomes and Community Health; SELMA, Swedish Environmental, Longitudinal, Mother and Child, Asthma and Allergy; SCDS, Seychelles Child Development Study

### Exposure and outcomes

Of the 81 included studies, 69 studies (85.2%) reported seafood and toxicant exposures during pregnancy or lactation, of which 52 studies (75.4%) were during pregnancy, 4 studies (5.8%) were during lactation, and 13 studies (18.8%) were during pregnancy and lactation. Of the 81 studies, 14 studies (17.2%) reported seafood and toxicant exposures during childhood.

Among the studies with exposures during pregnancy or lactation, persistent organic pollutants included the following: polychlorinated biphenyls (PCBs; *n* = 11) [21,41,43,51,61,63,66,71,95,97,98], perfluoroalkyl and polyfluoroalkyl substances (PFAS/PFCs; *n* = 8) [23,32,36,40,45,90,97,101], dioxin and dioxin-like compounds (DLCs; *n* = 3) [44,66,92], and polybrominated diphenyl ethers (PBDEs; *n* = 3) [71,97,98]. No evidence was available for polybrominated biphenyls or polycyclic aromatic hydrocarbons. Studies reporting heavy metal exposures assessed the following: mercury (Hg; *n* = 49) [22,24,26,27,30,31,34,[37], [38], [39],[41], [42], [53],[47], [48], [49], [50],^52^,^56^,^57^,^60^,[62], [63], [64], [65],[67], [68], [69], [70], [71], [72],[77], [78], [79], [80], [81],[84], [85], [86], [87], [88],[91], [92], [93], [94],^96^,^97^,^99^,^100^], methylmercury (MeHg; *n* = 13) [22,28,29,33,35,62,77,79,85,[87], [88], [89],^92^], lead (Pb; *n* = 9) [33,34,50,52,71,79,80,87,97], arsenic (As; *n* = 4) [62,71,87,97], and cadmium (Cd; *n* = 4) [34,71,87,97]. Essential trace elements included the following: selenium (Se; *n* = 11) [30,33,37,38,42,62,70,79,80,86,87], zinc (Zn; *n* = 3) [79,80,87], iron (Fe; *n* = 2) [28,87], and magnesium (Mg; *n* = 1) [87]; no study evaluated iodine. For pesticide exposures, included studies reported dichlorodiphenyltrichloroethane (DDT; *n* = 5) [43,61,71,95,97], whereas none assessed aldrin, dieldrin, chlordane, and chlorpyrifos. Finally, no studies reported microplastics.

Outcomes from studies during pregnancy or lactation varied. These included the following: neurodevelopment (*n* = 35) [[22], [23], [24],^28^,^29^,^33^,[36], [37], [38], [39],^53^,^44^,[47], [48], [49], [50], [51],^56^,^57^,^60^,[67], [68], [69], [70],[79], [80], [81],[85], [89],^90^,[92], [93], [94],^96^,^99^], child exposure biomarkers (*n* = 22) [21,22,32,34,35,41,53,45,51,52,62,64,66,71,77,85,87,88,90,95,99,101], growth (*n* = 17) [21,26,27,30,34,36,41,43,60,61,63,65,72,78,86,91,98], cardiometabolic (*n* = 3) [42,84,97], chronic disease indicators (*n* = 2) [31,84], and immune-related (*n* = 1) [31] outcomes. Additionally, other child outcomes included Apgar scores (*n* = 1) [34], alterations in inflammatory biomarkers (*n* = 1) [84], and preterm birth (*n* = 2) [100,101]. Overall, the seafood toxicant–outcome pairs with sufficient evidence were Hg and neurodevelopmental [22,24,[37], [38], [39],^53^,[47], [48], [49], [50],^56^,^57^,^60^,[67], [68], [69], [70],[79], [80], [81],^85^,[92], [93], [94],^96^,^99^], growth [21,26,27,30,34,41,60,63,65,72,78,86,91], child exposure biomarkers [34,41,53,52,62,64,71,77,85,87,88,99], and cardiometabolic [42,84,97] outcomes; MeHg and growth [21,26,27,30,34,41,60,63,65,72,78,86,91], and child exposure biomarkers [35,62,77,83,85,87,88] outcomes; Pb and child exposure biomarkers [34,52,71,87] and neurodevelopmental [33,50,79,80] outcomes; PCBs and child exposure biomarkers [41,51,66,71,95] and growth [41,43,61,63,98] outcomes; and, PFAS/PFCs [32,45,90,101] and As [62,71,87] and child exposure biomarkers. The evidence map for toxicant–outcome pairs from exposure during pregnancy and lactation is shown in Table 3.

**TABLE 3:**
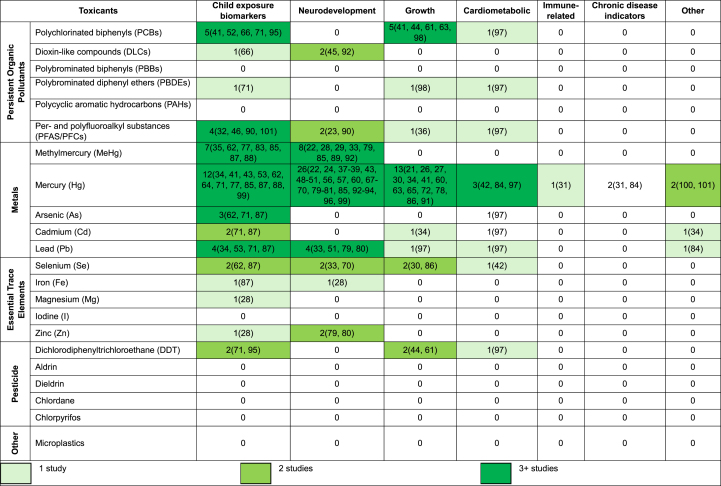
Existing evidence on seafood toxicant exposure among pregnant and lactating adults and child outcomes.

Fewer studies examined exposure in children (14 studies). The included studies reported persistent organic pollutants as follows: PFAS/PFCs (*n* = 2) [23,97], PCBs (*n* = 1) [97], and PBDEs (*n* = 1) [97]. No studies assessed DLCs, polybrominated biphenyls, and polycyclic aromatic hydrocarbons. Studies that evaluated heavy metals included the following: Hg (*n* = 7) [46,54,58,59,73,75,97], As (*n* = 4) [54,74,82,97], Cd (*n* = 4) [54,73,76,97], and Pb (*n* = 3) [54,73,97], MeHg (*n* = 2) [[25], [55]]. Studies reporting essential trace elements included Zn (*n* = 2) [54,73], Se (*n* = 1) [73], Fe (*n* = 1) [54], and Mg (*n* = 1) [54]; no studies reported iodine. DDT (*n* = 1) [97] was the only pesticide reported; no studies reported aldrin, dieldrin, chlordane, or chlorpyrifos. No studies reported microplastic exposures.

The studies in children assessed outcomes related to neurodevelopment (*n* = 9) [23,46,55,58,59,[73], [74], [75], [76]], child exposure biomarkers (*n* = 4) [54,55,74,[82], [83]], cardiometabolic (*n* = 2) [25,97], immune-related (*n* = 2) [54,82], and growth (*n* = 1) [55]. No study evaluated the relationship among seafood intake, seafood toxicants, and chronic disease indicators among children. The only seafood toxicant–outcome pair among children with sufficient evidence for consideration for systematic review was Hg and neurodevelopmental outcome [46,58,59,73,75]. The evidence map for toxicant–outcome pairs in exposed children is shown in Table 4.

**TABLE 4:**
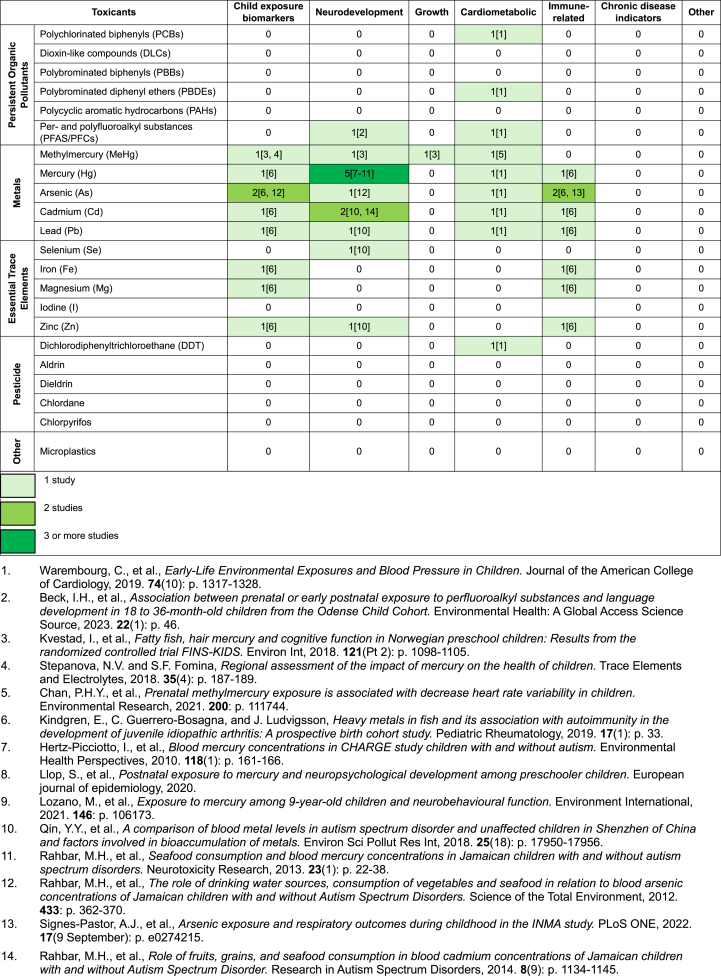
Existing evidence on seafood toxicant exposure among children and child outcomes.

## Discussion

In this review, we present the scope of the evidence involving toxicant exposures from seafood during childhood, pregnancy, and lactation, and child outcomes. Although we identified studies reporting PCBs, DLCs, PBDEs, PFAS/PFCs, Hg, MeHg, Pb, As, Cd, Se, Zn, Fe, Mg, and DDT, there was no evidence related to other toxicants prioritized by the NASEM committee [15]. Of the toxicant–outcome pairs for which studies were identified, 12 had sufficient evidence to warrant consideration for systematic review. These included studies of maternal populations that examined Hg and child exposure biomarkers, neurodevelopmental, growth, and cardiometabolic outcomes; MeHg and child exposure biomarkers and neurodevelopmental outcomes, Pb and child exposure biomarkers and neurodevelopmental outcomes; PCBs and child exposure biomarkers and growth outcomes; and As and PFAS/PFCs and child exposure biomarkers outcome. The evidence was limited for childhood exposure with sufficient evidence identified for only 1 toxicant–outcome pair, namely Hg and neurodevelopmental outcomes.

The purpose of this ScR was to identify peer-reviewed literature containing data on seafood toxicant exposure and outcome pairs, but not to examine or estimate the relationships between them. For those exposure–outcome pairs for which we identified sufficient evidence, a systematic review and (where possible) meta-analysis would be required to draw conclusions regarding the association between specific toxicant exposure during pregnancy, lactation, or childhood on child outcomes.

For this ScR, we defined sufficient evidence as 3 or more studies to warrant consideration for conducting a de novo systematic review, a pragmatic decision that balanced the amount of resources required to perform a systematic review and the likelihood of identifying an evidence base that might meaningfully inform policy. However, it should be noted that even for the evidence deemed sufficient, there may be limitations identified upon systematic review that hinder its utility or the ability to combine data. For example, there could be substantial heterogeneity limiting the ability to synthesize the evidence. We found that the countries and populations included in the evidence varied considerably in terms of typical diet and the amount of seafood consumed. Some countries, such as Iceland, typically consume high levels of seafood, whereas others, such as Mongolia, consume less [102]. These differences in baseline seafood consumption can impact nutrient and toxicant exposure, and ultimately impact their relation to child outcomes. Further, differences in the type and distribution of seafood intake within a population could also impact the ability to detect an association if one truly exists.

Another source of heterogeneity includes outcome assessment methods. A systematic review on seafood intake during pregnancy and lactation on child developmental outcomes noted several types of outcome measures and different outcome ascertainment methods within any given outcome domain [103]. For example, there were 8 specific outcomes within cognitive development; for each outcome, different assessment methods were applied, most of which had multiple subscales. Across 21 articles, 156 different results related to maternal seafood intake and child cognitive development were identified. Thus, although this ScR identified seafood toxicant exposure–outcome pairs with sufficient evidence for a systematic review, it is only the beginning of assessing the totality of the evidence related to any one of them.

Although too few studies can be problematic for conducting systematic reviews, toxicant–outcome pairs with many studies may benefit from an overview of existing systematic reviews (i.e. an umbrella review) rather than a de novo systematic review. This ScR identified 72 studies related to Hg and MeHg exposure on child outcomes. To avoid research waste (both time and resources), an option for topics with large evidence bases is to determine whether recent relevant systematic reviews exist. If many systematic reviews are identified that match a specific research question, they can be summarized in an overview of reviews.

This ScR has strengths and limitations. To identify relevant studies that assessed the relationship between toxicant exposures through seafood and child outcomes, articles had to measure both seafood intake and toxicant exposure, as well as report an assessment of a potential association between the 2 exposures and a child outcome, which may have been limiting. For example, MeHg concentrations generally reflect Hg sourced from seafood intake—there are limited other environmental sources of exposure for the general population; however, studies that reported MeHg without measuring seafood intake were excluded because they did not meet our strict inclusion criteria. As such, additional relevant evidence may be available when examining Hg from seafood. Further, because the purpose of this ScR was to support nutrition recommendations in the United States, included studies were restricted to countries ranked high or very high on the Human Development Index. Although this is important for generalizing to the United States population, this excludes data from low-income and middle-income countries, some of which may consume high amounts of seafood as a critical protein source and/or have seafood that is more heavily contaminated with toxicants, thereby making it more likely that associations for adverse outcomes may be identified. By design, an ScR does not assess risk of bias; therefore, future systematic reviews are needed to evaluate sources of bias and the certainty of evidence for specific toxicant–outcome pairs. Finally, an ScR is not designed to assess the association, or any effect size or direction of an association, but rather to identify the available evidence on a topic—this would require a systematic review and, where possible, meta-analysis.

In conclusion, several seafood toxicant exposure and child outcome pairs were identified that warrant further investigation, either as a systematic review or, where extensive evidence exists, such as with Hg or MeHg, an overview of reviews (i.e. an umbrella review). The ScR also identified substantial evidence gaps where there was no or very little evidence related to the prioritized toxicants of concern. Additionally, it would be useful to conduct a similar ScR focusing on seafood toxicant exposure and outcomes in adults to inform dietary guidelines for additional age and sex groups. The results of this ScR will guide future research on these important public health topics.

## Author contributions

The authors’ responsibilities were as follows—MKS, AJM: designed the research study; MKS, RT, SS, RCT, JSD: conducted the research; RT, MKS: assessed the data and developed the manuscript; RT, MKS, AJM: critically reviewed and edited the manuscript; and all authors: have read and approved the final manuscript.

## Funding

This work was supported by a contract with the National Academies of Sciences, Engineering, and Medicine.

## Data availability

Data described in this study are presented with this manuscript.

## Conflict of interest

National Academies of Science provided financial support to Texas A&M Agriculture, Food and Nutrition Evidence Center for this study. All authors declare that they have no known competing financial interests or personal relationships that could have appeared to influence the work reported in this paper.
