# Supplementary material for: Seafood Toxicant Exposure During Pregnancy, Lactation, and Childhood and Child Outcomes: A Scoping Review
Source: Adv Nutr. 2024 Dec 10;16(1):100353. doi: 10.1016/j.advnut.2024.100353 (PMC11784783; doi:10.1016/j.advnut.2024.100353)
Supplement: multimedia component 1 [file mmc1.docx]

**Supplementary Materials**

**Seafood toxicant exposure during pregnancy, lactation, and childhood and child outcomes: A scoping review**

Rupal Trivedi

**Supplementary Table 1.** Search strategy used to identify studies

**Supplementary Table 2**. Reasons for excluding articles during full text screening

**Supplementary Table 1: Search strategy used to identify studies**

Number of records identified from each database searched:

Embase: 3195

PubMed: 2867

Cochrane Central Register of Controlled Trials (CENTRAL): 30

**Database #1: Embase**

- Provider: Ovid
- Date(s) Searched: July 12, 2023
- Date range searched: From January 1, 2000
- Search Terms:

| Embase <1980 to 2023 Week 27> | | |
| --- | --- | --- |
| 1 | exp sea food/ or exp fish/ or exp fish consumption/ or exp fish protein/ or exp fish product/ or exp fish flour/ or exp shellfish/ or exp shellfish protein/ or exp mercurialism/ or exp methylmercury/ or exp shark/ or exp swordfish/ or exp tuna/ or exp salmonine/ or exp sardine/ or exp gadiformes/ or exp flounder/ or exp atlantic cod/ or exp cod/ or exp tilapia/ or exp shrimp/ or exp oyster/ or exp mya/ or exp bivalve/ or exp clam/ or exp scallop/ or exp brachyura/ or exp crab/ or exp perciformes/ or exp mackerel/ or exp lobster/ or exp decapodiformes/ or exp squid/ or exp halibut/ or exp crayfish/ or exp anchovy/ or exp herring/ or exp rockfish/ or exp cetacea/ or exp perch/ or exp bass/ or exp lake trout/ or exp catfish/ or exp sushi/ or exp salmo salar/ or exp salmonid/ | 321379 |
| 2 | (seafood or sea foods or sea food or sea-food or sea-foods or fish consumption or fishes or fish protein or fish proteins or fish product or fish products or fish meal or fish flour or fatty fish or shellfish or shellfish proteins or mercurialism or mercury poisoning or methylmercury or sharks or swordfish or tuna or salmonine or salmon or sardine* or sardines or gadiformes or pollock or flounder or cod or tilapia or shrimp or oyster or oysters or clams or scallops or crab or crabs or perciformes or mackerel or catfishes or trout or lobster or decapodiformes or squid or halibut or mahi mahi or crayfish or crawfish or anchovy or herring or rockfish or marine product or marine products or marlin or orange roughy or tile fish or whales or perch or walleye fish or lake trout or salmonid or catfish or sushi or cerviche or sashimi or gravlax or tuna tartare or seafood crudo or fluke crudo or fluke or carpaccio or e-la-ota or poke or hinava or gohu ikan or esqueixada or kelaguen or namero or kilawin or stroganina or yusheng or koi pla or kokoda or kuai or lakerda or larb pla or ota ika or tiradito or xato or umai or salmo salar or atlantic salmon or salmonids).ti,ab,kw. | 204809 |
| 3 | or/1-2 | 409899 |
| 4 | exp diet/ or exp dietary supplement/ or exp food/ or exp nutritional status/ or exp food analysis/ or exp nutritional value/ or exp iron/ or exp folic acid/ or exp calcium/ or exp zinc/ or exp phytate/ or exp phytic acid/ or exp polyphenol/ or exp omega 3 fatty acid/ or exp fatty acid/ or exp fish protein/ or exp mediterranean diet/ or exp fish consumption/ or exp eicosapentaenoic acid/ or exp docosapentaenoic acid/ or exp food intake/ or exp docosahexaenoic acid/ or exp meal/ or exp dietary intake/ or exp protein/ or exp vitamin D/ or exp choline/ or exp taurine/ or exp Iodine/ or exp selenium/ or exp drinking/ or exp eating/ or exp infant feeding/ or exp meat consumption/ or exp amino acid intake/ or exp dietary reference intake/ or exp fat intake/ or exp mineral intake/ or exp iodine intake/ or exp nutrient intake/ or exp protein intake/ or exp tolerable daily intake/ or exp vitamin intake/ or exp magnesium/ | 3808867 |
| 5 | (diet or dietary supplement or dietary supplements or food or nutritional status or iron or folic acid or calcium or Zinc or phytate or phytic acid or polyphenol or polyphenols or fish species or fish tissue or fish intake or fish-rich diet or fatty acids or fatty acid or fish protein or mediterranean diet or omega-3 fatty acids or omega 3 fatty acids or omega-3 fatty acid or omega 3 fatty acid or fish consumption or eicosapentaenoic acid or docosahexaenoic acid or docosapentaenoic acid or meals or food intake or dietary intake or magnesium).ti,ab,kw. | 2309077 |
| 6 | or/4-5 | 4726709 |
| 7 | exp organomercury compound/ or exp dioxins/ or exp polychlorinated biphenyl/ or exp methylmercury/ or exp monomethylmercury/ or exp dioxin/ or exp mercury/ or exp inorganic mercury/ or exp campheclor/ or exp ddt/ or exp chlordane/ or exp dieldrin/ or exp aldrin/ or exp endrin/ or exp heptachlor/ or exp hexachlorobenzene/ or exp cyclohexane/ or exp brominated flame retardant/ or exp diphenyl ether/ or exp polybrominated diphenyl ether/ or exp aquatic toxicology/ or exp aquatic toxicity/ or exp arsenic/ or exp dibenzofuran/ or exp fluorocarbon/ or exp microplastic/ or exp nanofiber/ or exp nanoplastic/ or exp cadmium/ or exp lead/ or exp chlorpyrifos/ or exp algal bloom/ or exp cyanobacterium/ or exp ciguatera/ or exp scombroid poisoning/ or exp domoic acid/ or exp Hepatitis A virus/ or exp Salmonella/ or exp Escherichia coli/ | 776305 |
| 8 | (polyfluoroalkyl substance* or pfas or methylmercury compounds or methylmercury-toxicity or organomercury compound or organic mercury compound or mercury exposure* or organomercury or methylmercury-induced cytotoxicity or organomercurials or mercurial* or polychlorinated biphenyl or polychlorinated biphenyls or polychlorobiphenyl compounds or methylmercury or methyl mercury or methyl-mercury or mehg or monomethylmercury or methylquecksilber or dioxin or dioxins or inorganic mercury or pfos or pfoa or campheclor or toxaphene or dichlorodiphenyltrichlorethane or chlordane or chlordan or dieldrin or hexachlorobenzene or cyclohexane or brominated flame retardant* or diphenyl ether or diphenyl ethers or polybrominated diphenyl ether or polybrominated diphenyl ethers or aquatic toxicology or aquatic toxicity or aldrin or arsenic or dibenzofuran* or endrin or fluorocarbon* or furan* or heptachlor or microfiber* or microplastic* or nanofiber* or nanoplastic* or organic-fluorine* or polyfluoroalkyl substance* or total organic fluorine or cadmium or 208Pb or inorganic-lead or lead-208 or lead-ion or lead-ore or lead-radioisotope or lead-radioisotopes or plumbum or radioisotope-lead or chloropyrifos or chlorpyrifos-ethyl or chlorpyriphos or dursban or lorsban or o-3-5-6-trichloropyrid-2-yl-o-o-diethyl-phosphorothioate or o-o-diethyl-o-3-5-6-trichloropyrid-2-yl-phosphorothioate or o-o-diethyl-o-3-5-6-trichloro-2-pyridyl-phosphorothioate or o-o-diethyl-o-3-5-6-trichloro-2-pyridylphosphorothioate or phosphorothioic-acid-o-o-diethyl-o-3-5-6-trichloropyrid-2-yl-ester or harmful-algal-bloom or phytoplankton-bloom or blue-green-alga or blue-green-algae or blue-green-bacteria or blue-green-bacterium or blue-green-algae or Cyanobacteria or Cyanophyceae or Cyanophyta or gram-negative-oxygenic-photosynthetic-bacteria or ciguatera or histamine-fish-poisoning or histamine-food-poisoning or scombroid or scombroid-poisoning or scombroid-fish-intoxication or scombroid-fish-poisoning or scombroid-food-poisoning or scombroid-ichthyotoxicosis or scombroid-intoxication or scombroid-syndrome or scombrotoxic-fish-poisoning or scombrotoxic-poisoning or scombrotoxin-fish-poisoning or scombrotoxin-poisoning or scombrotoxism or domoic-acid or domoate or hepatitis-A or epidemic-hepatitis-virus or HAV or Hepatitis-virus-A or Hepatovirus-A or infectious-hepatitis-virus or bacillus-paratyphi-alcaligenes or bacillus-paratyphosus or alkalescens-dispar-group or Bacillus-coli or Bacillus-escherichii or Bacterium-coli or Bacterium-coli-commune or bacterium-E3 or coli-bacillus or coli-bacterium or colibacillus or colon-bacillus or E-coli or E-coli or E-coli or Enterococcus-coli or Escherichia-alkalescens-dispart or Escherichia-coli-0100 or Escherichia-coli-0124 or Escherichia-coli-8 or Escherichia-coli-strain or Escherichia-coli-suspension).ti,ab,kw. | 439955 |
| 9 | or/7-8 | 909634 |
| 10 | exp Mental Disease/ or exp Cognition/ or exp metacognition/ or exp Depression/ or exp Dementia/ or exp anxiety/ or exp Psychomotor Performance/ or exp motor performance/ or exp Executive Function/ or exp Child Behavior Disorder/ or exp developmental disorder/ or exp autism/ or exp language processing/ or exp language delay/ or exp Child Development/ or exp developmental delay/ or exp motor skill/ or exp Problem Solving/ or exp academic achievement/ or exp mental health/ or exp mental function/ or exp Congenital Disorder/ or exp congenital malformation/ or exp Mortality/ or exp Morbidity/ or exp chronic disease/ or exp environmental disease/ or exp child nutrition/ or exp low birth weight/ or exp Irritability/ or exp Shyness/ or exp Tremor/ or exp blindness/ or exp hearing impairment/ or exp Memory/ or exp Attention/ or exp verbal memory/ or exp Acrodynia/ or exp Kidney disease/ or exp Hypertension/ or exp tubular dysfunction/ or exp malignant neoplasm/ or exp gross motor/ or exp ataxia/ or exp fine motor/ or exp learning disorder/ or exp reading/ or exp peripheral neuropathy/ or exp neuropathy/ or exp weakness/ or exp areflexia/ or exp paresthesia/ or exp motor coordination/ or exp functional disability/ or exp visual impairment/ or exp behavior disorder/ or exp school readiness/ or exp sleep disorder/ or exp asperger syndrome/ or exp Cardiovascular disease/ or exp heart death/ or exp Neoplasm/ or exp Hypersensitivity/ or exp non insulin dependent diabetes mellitus/ or exp Asthma/ or exp allergy/ or exp atopy/ or exp Central nervous system/ or exp Immune function/ or exp cell proliferation/ or exp cell differentiation/ or exp apoptosis/ or exp Fracture/ or exp falling/ or exp thyroid gland/ or exp thyroxine/ or liothyronine/ or exp goiter/ or exp growth disorder/ or exp blood pressure/ or exp hypotension/ or exp muscle weakness/ or exp oxidative stress/ or exp immunity/ or exp health hazard/ or exp risk factor/ or exp risk assessment/ or exp outcome assessment/ or exp infection/ or exp sexual maturity/ or exp Sexual maturation/ or exp puberty/ or exp thyroid diseases/ or exp thyroid disease/ or exp prematurity/ or exp sensitization/ or exp Inflammation/ or exp Autoimmunity/ or exp Autoimmune Disease/ or exp Brain/ or exp attention deficit hyperactivity disorder/ or exp Alzheimer disease/ or exp behavior disorder/ or exp blindness/ or exp malignant neoplasm/ or exp heart death/ or exp congenital disorder/ or exp coronary artery disease/ or exp growth/ or exp child growth/ or exp "toxicity and intoxication"/ or exp biological marker/ or exp cholesterol/ or exp low density lipoprotein cholesterol/ or exp triacylglycerol/ or exp high density lipoprotein/ or exp failure to thrive/ or exp malnutrition/ or exp protein deficiency/ or exp dyslipidemia/ | 24296024 |
| 11 | (mental disorder or mental disorders or cognitive or neurocognitive or neurodevelop* or neurological or Alzheimer* or senility or senile or presenile or motor skills or attention-deficit-disorder or attention-deficit-disorders or ADHD or Autism-Spectrum-Disorder or Asperger or academic performance or academic failure or academic success or mental process* or congenital abnormality or congenital abnormalities or birth defects or birth defect or chronic condition* or changes-in-vision or vision problem or changes-in-hearing or deafness or gastrointestinal or respiratory or obstruction-of-visual-field or pink disease or renal disease* or tubular injury or cancer or learning disabilit* or communication* or social-interactive-skills or functional disabilities or functional disability or sensorineural defect or sensorineural defects or vision loss or hearing loss or psychological disorder* or behavioral disorder* or Asperge* or coronary disease* or cardiac death or Growth-and-development or diabetes-mellitus-Type-2 or Metabolic outcome* or accidental fall* or thyroid* or triiodothyronine or mental-growth-retardation or immune-system-phenomena or hazardous substances or hazardous substance or risk factors or risk factor or risk-benefit or thyroid dysfunction or thyroid defect or thyroid defects or pre-term or neurodevelopmental disorder* or cardiovascular disease* or toenail biomarker or neurotoxicity syndrome* or academic achievement* or acrodynia or allergy or anxiety or apoptosis or areflexia or asthma or ataxia or atopy or attention or autism or autoimmune disease* or autoimmunity or blood pressure or cell differentiation or cell proliferation or central nervous system or child behavior disorder* or child behaviour disorder* or child development or child nutritional physiological phenomena or child nutritional physiological phenomena or chronic disease* or cognition or dementia or depression or developmental delay* or developmental disorder* or environmental disease or disorders-of-environmental-origin or executive function or fine motor or bone-fracture* or fractures-bone or goiter or gross motor or health hazard or hypersensitivity or hypertension or hypotension or immune function or immune system phenomena or immunity or inflammation or irritability or kidney disease or language delay* or language processing or low birth weight or memory or mental growth retardation or mental health or metacognition or morbidity or mortality or motor coordination or muscle weakness or neoplasms or neuropathy or outcome assessment or oxidative stress or paresthesia or prematurity or premature birth or problem solving or psychomotor performance or puberty or reading or risk assessment* or school readiness or sensitization or sexual maturation or sexual maturity or shyness or sleep disorder* or social interactive skill* or tremor or tubular dysfunction or tubular injury or verbal memory or vision loss or vision problem or weakness or 3-hydroxy-5-cholestene or 3beta-hydroxy-5-cholestene or 3beta-hydroxycholest-5-ene or 5-cholesten-3beta-ol or beta-cholesterol or cholest-5-en-3beta-ol or cholest-5-ene-3-ol or cholesterin or cholesterine or cholesterol-release or dythol or nsc-8798 or cholesterol-LDL- or LDL-cholesterol or lipoproteins-LDL-cholesterol or acylglycerol-tri or fatty-acid-triglyceride or triacyl-glyceride or triglyceride or triglycerides- or tryglyceride or alpha-7-lipoprotein or alpha-lipoprotein or HDL or high-density-lipoprotein-phospholipid or lipoprotein-alpha or lipoprotein-high-density or lipoproteins-hdl or pre-alpha-lipoprotein or very-high-density-lipoprotein or failure-to-thrive or deficient-nutrition or malnourishment or severe-acute-malnutrition- or underfeeding or undernourishment or undernutrition or deficiency-protein or dietary-protein-deficiency or dyslipaemia or dyslipemia or dyslipidaemia or dyslipidaemias or dyslipidemias- or lipidaemia-dys or lipidemia-dys).ti,ab,kw. | 12879897 |
| 12 | or/10-11 | 25761733 |
| 13 | 3 and 6 and 9 and 12 | 14303 |
| 14 | 13 not ((exp animal/ or exp invertebrate/ or nonhuman/ or animal experiment/ or animal tissue/ or animal model/ or exp plant/ or exp fungus/) not (exp human/ or human tissue/)) | 6234 |
| 15 | limit 14 to (english language and yr="2000 -Current") | 5377 |
| 16 | 15 and (article/ or article in press/) | 3167 |
| 17 | 16 not (conference abstract/ or conference review/ or conference paper/ or editorial/ or erratum/ or letter/ or note/ or review/ or systematic review/ or meta analysis/) | 3145 |
| 18 | exp Macaca/ or Haplorhini/ or Catarrhini/ or exp Platyrrhini/ or exp Gorilla/ or exp Pan paniscus/ or exp Pan troglodytes/ or hominid/ or exp chimpanzee/ or exp orangutan/ or exp Cercopithecidae/ or exp Hylobatidae/ or exp tarsiiform/ or exp prosimian/ or primate/ | 131426 |
| 19 | (ape* or macaca* or macaque* or allenopithecus or allocebus or alouatta or alouattinae or angwantibo* or anthropoid or anthropoidea or anthropoids or aotes or aotidae or aotinae or aotus or ape or apes or arctocebus or ateles or atelidae or atelinae or avahi or aye-aye* or baboon or baboons or bonobo or bonobos or brachyteles or bushbabies or bushbaby or cacajao or callibella or callicebinae or callicebus or callimico or callithrichid* or callithrichinae or callithrix or callitrichid or callitrichidae or callitrichide or callitrichids or callitrichinae or capuchin or capuchins or carlito syrichta or catarhine* or catarhini or catarrhina or catarrhine* or catarrhini or cebid or cebidae or cebids or cebinae or ceboidea or cebuella or cebus or cephalopachus or cercocebus or cercopithecid* or cercopithecinae or cercopithecine* or cercopithecini or cercopithecoid or cercopithecoidea or cercopithecoids or cercopithecus or cheirogaleidae or cheirogaleus or cheracebus or chimp or chimpanzee or chimpanzees or chimps or chiromyiformes or chiropotes or chlorocebus or colobidae or colobinae or colobine* or colobini or colobus* or cynomolgus or daubentonia or daubentoniidae or douc or doucs or erythrocebus or eulemur or euoticus or euprimate* or galagid* or galago or galagoides or galagonidae or galagos or gelada or geladas or gibbon or gibbons or gorilla or gorillas or grivet or grivets or guenon* or guereza* or hapalemur or haplorhine* or haplorhini or haplorrhine* or haplorrhini or hominid* or hominin or homininae or hominine or hominines or hominini or hominins or hominoidea or hoolock or howler* or hylobates or hylobatidae or indri or indridae or indriid* or indris or kipunji* or lagothrix or langur or langurs or lemur or lemurid* or lemuriform or lemuriformes or lemuriforms or lemurinae or lemuroidea or lemurs or leontideus or leontocebus or leontopithecus or lepilemur or lepilemurid* or lesula* or lophocebus or loriform or loriformes or lorinae or loris or lorises or lorisid* or lorisiform* or lorisinae or lorisoid* or lutung or lutungs or macaca or macaque's or macaque or macaques or malbrouck* or mandrill or mandrills or mandrillus or mangabey* or marmoset or marmosets or mico argentatus or mico chrysoleucos or mico emiliae or mico humilis or mico marcai or mico melanurus or mico rondoni or microcebus or miopithecus or mirza coquereli or mirza zaza or monkey or monkeys or muriqui* or nasalis larvatus or nomascus or nycticebus or oedipomidas or orang utan* or orang-utan* or orangutan* or oreonax or otolemur or pan paniscus or pan troglodytes or panin or panina or panins or papio or papionini or paragalago or perodicticinae or perodicticus or phaner or piliocolobus or pithecia or pithecidae or pitheciid* or pitheciinae or pithecinae or platyrhine* or platyrhini or platyrrhina or platyrrhine* or platyrrhini or plecturocebus or pongid* or ponginae or pongo or potto or pottos or presbytini or presbytis or primate or primates or procolobus or prolemur or propithecus or prosimian* or prosimii or pseudopotto or pygathrix or rhinopithecus or rungwecebus or saguinus or saimiri or saimiriinae or sapajus or sciurocheirus or semnopithecus or siamang or siamangs or sifaka or sifakas or simians or simias or simiiform* or strepsir* or surili* or symphalangus or talapoin* or tamarin or tamarins or tamarinus or tarsier or tarsiers or tarsiid* or tarsiiform* or tarsius or theropithecus or trachypithecus or uacari* or uakari or uakaris or varecia or vervet*).ti,ab,kw. | 285728 |
| 20 | or/18-19 | 303922 |
| 21 | 13 and 20 | 83 |
| 22 | limit 21 to (english language and yr="2000 -Current") | 66 |
| 23 | 22 and (article/ or article in press/) | 50 |
| 24 | 23 not (conference abstract/ or conference review/ or conference paper/ or editorial/ or erratum/ or letter/ or note/ or review/ or systematic review/ or meta analysis/) | 50 |

**Database #2: PubMed**

- Provider: U.S. National Library of Medicine
- Date(s) Searched: July 12, 2023
- Date range searched: From January 1, 2000
- Search Terms:

| 1 | "Brachyura"[Mesh] OR "Perciformes"[Mesh] OR "Decapodiformes"[Mesh] OR "Flounder"[Mesh] OR "Fish Flour"[Mesh] OR "Mya"[Mesh] OR "Bass"[Mesh] OR "Gadiformes"[Mesh] OR "Fish Proteins"[Mesh] OR "Fish Products"[Mesh] OR "Shellfish Proteins"[Mesh] OR "Salmo salar"[Mesh] OR "Tilapia"[Mesh] OR "Tuna"[Mesh] OR "Shellfish"[Mesh] OR "Methylmercury Compounds"[Mesh] OR "Cetacea"[Mesh] OR "Gadus morhua"[Mesh] OR "Bivalvia"[Mesh] OR "Seafood"[Mesh] OR "Catfishes"[Mesh] OR "Pectinidae"[Mesh] OR "Sharks"[Mesh] OR "Salmonidae"[Mesh] OR "Perches"[Mesh] OR "Ostreidae"[Mesh] OR "Astacoidea"[Mesh] OR "Fishes"[Mesh] OR "Fish Proteins, Dietary"[Mesh] OR "Mercury Poisoning"[Mesh] or seafood[tiab] or sea foods[tiab] or sea food[tiab] or sea-food[tiab] or sea-foods[tiab] or fish consumption[tiab] or fishes[tiab] or fish protein[tiab] or fish proteins[tiab] or fish product[tiab] or fish products[tiab] or fish meal[tiab] or fish flour[tiab] or fatty fish[tiab] or shellfish[tiab] or shellfish proteins[tiab] or mercurialism[tiab] or mercury poisoning[tiab] or methylmercury[tiab] or sharks[tiab] or swordfish[tiab] or tuna[tiab] or salmonine[tiab] or salmon[tiab] or sardine*[tiab] or sardines[tiab] or gadiformes[tiab] or pollock[tiab] or flounder[tiab] or cod[tiab] or tilapia[tiab] or shrimp[tiab] or oyster[tiab] or oysters[tiab] or clams[tiab] or scallops[tiab] or crab[tiab] or crabs[tiab] or perciformes[tiab] or mackerel[tiab] or catfishes[tiab] or trout[tiab] or lobster[tiab] or decapodiformes[tiab] or squid[tiab] or halibut[tiab] or mahi mahi[tiab] or crayfish[tiab] or crawfish[tiab] or anchovy[tiab] or herring[tiab] or rockfish[tiab] or marine product[tiab] or marine products[tiab] or marlin[tiab] or orange roughy[tiab] or tile fish[tiab] or whales[tiab] or perch[tiab] or walleye fish[tiab] or lake trout[tiab] or salmonid[tiab] or catfish[tiab] or sushi[tiab] or cerviche[tiab] or sashimi[tiab] or gravlax[tiab] or tuna tartare[tiab] or seafood crudo[tiab] or fluke crudo[tiab] or fluke[tiab] or carpaccio[tiab] or e-la-ota[tiab] or poke[tiab] or hinava[tiab] or gohu ikan[tiab] or esqueixada[tiab] or kelaguen[tiab] or namero[tiab] or kilawin[tiab] or stroganina[tiab] or yusheng[tiab] or koi pla[tiab] or kokoda[tiab] or kuai[tiab] or lakerda[tiab] or larb pla[tiab] or ota ika[tiab] or tiradito[tiab] or xato[tiab] or umai[tiab] or salmo salar[tiab] or atlantic salmon[tiab] or salmonids[tiab] |  |
| --- | --- | --- |
| 2 | "Phytic Acid"[Mesh] OR "Food Analysis"[Mesh] OR "Diet, Mediterranean"[Mesh] OR "Taurine"[Mesh] OR "Folic Acid"[Mesh] OR "Eicosapentaenoic Acid"[Mesh] OR "Zinc"[Mesh] OR "Vitamin D"[Mesh] OR "Selenium"[Mesh] OR "Nutritional Status"[Mesh] OR "Iron"[Mesh] OR "Iodine"[Mesh] OR "Food"[Mesh] OR "Drinking"[Mesh] OR "Diet"[Mesh] OR "Choline"[Mesh] OR "Calcium"[Mesh] OR "Diet, Food, and Nutrition"[Mesh] OR "Fatty Acids, Omega-3"[Mesh] OR "Docosahexaenoic Acids"[Mesh] OR "Dietary Supplements"[Mesh] OR "Meals"[Mesh] OR "Fatty Acids"[Mesh] OR "Fish Proteins"[Mesh] OR "Proteins"[Mesh] OR "Nutritive Value"[Mesh] OR "Recommended Dietary Allowances"[Mesh] OR "Polyphenols"[Mesh] OR "Diet, Healthy"[Mesh] OR "Diet Records"[Mesh] OR "Infant Nutritional Physiological Phenomena"[Mesh] OR "Eating"[Mesh] or Magnesium[mh] or diet[tiab] or dietary supplement[tiab] or dietary supplements[tiab] or food[tiab] or nutritional status[tiab] or iron[tiab] or folic acid[tiab] or calcium[tiab] or Zinc[tiab] or phytate[tiab] or phytic acid[tiab] or polyphenol[tiab] or polyphenols[tiab] or fish species[tiab] or fish tissue[tiab] or fish intake[tiab] or fish-rich diet[tiab] or fatty acids[tiab] or fatty acid[tiab] or fish protein[tiab] or mediterranean diet[tiab] or omega-3 fatty acids[tiab] or omega 3 fatty acids[tiab] or omega-3 fatty acid[tiab] or omega 3 fatty acid[tiab] or fish consumption[tiab] or eicosapentaenoic acid[tiab] or docosahexaenoic acid[tiab] or docosapentaenoic acid[tiab] or meals[tiab] or food intake[tiab] or dietary intake[tiab] or magnesium[tiab] |  |
| 3 | "Hexachlorobenzene"[Mesh] OR "Mercury"[Mesh] OR "Heptachlor"[Mesh] OR "Endrin"[Mesh] OR "Dieldrin"[Mesh] OR "DDT"[Mesh] OR "Arsenic"[Mesh] OR "Aldrin"[Mesh] OR "Mercury Compounds"[Mesh] OR "Mercury Poisoning, Nervous System"[Mesh] OR "Cyclohexane Monoterpenes"[Mesh] OR "Dioxins and Dioxin-like Compounds"[Mesh] OR "Fluorocarbons"[Mesh] OR "Nanofibers"[Mesh] OR "Polychlorinated Biphenyls"[Mesh] OR "Chlordan"[Mesh] OR "Arsenic Poisoning"[Mesh] OR "Microplastics"[Mesh] OR "Dioxins"[Mesh] OR "Methylmercury Compounds"[Mesh] or Cadmium[mh] or lead[mh] or chlorpyrifos[mh] or Harmful Algal Bloom[mh] or Cyanobacteria[mh] or Ciguatera Poisoning[mh] or Hepatitis A virus[mh] or Salmonella[mh] or Escherichia coli[mh] or polyfluoroalkyl substance*[tiab] or pfas[tiab] or methylmercury compounds[tiab] or methylmercury-toxicity[tiab] or organomercury compound[tiab] or organic mercury compound[tiab] or mercury exposure*[tiab] or organomercury[tiab] or methylmercury-induced cytotoxicity[tiab] or organomercurials[tiab] or mercurial*[tiab] or polychlorinated biphenyl[tiab] or polychlorinated biphenyls[tiab] or polychlorobiphenyl compounds[tiab] or methylmercury[tiab] or methyl mercury[tiab] or methyl-mercury[tiab] or mehg[tiab] or monomethylmercury[tiab] or methylquecksilber[tiab] or dioxin[tiab] or dioxins[tiab] or inorganic mercury[tiab] or pfos[tiab] or pfoa[tiab] or campheclor[tiab] or toxaphene[tiab] or dichlorodiphenyltrichlorethane[tiab] or chlordane[tiab] or chlordan[tiab] or dieldrin[tiab] or hexachlorobenzene[tiab] or cyclohexane[tiab] or brominated flame retardant*[tiab] or diphenyl ether[tiab] or diphenyl ethers[tiab] or polybrominated diphenyl ether[tiab] or polybrominated diphenyl ethers[tiab] or aquatic toxicology[tiab] or aquatic toxicity[tiab] or aldrin[tiab] or arsenic[tiab] or dibenzofuran*[tiab] or endrin[tiab] or fluorocarbon*[tiab] or furan*[tiab] or heptachlor[tiab] or microfiber*[tiab] or microplastic*[tiab] or nanofiber*[tiab] or nanoplastic*[tiab] or organic-fluorine*[tiab] or polyfluoroalkyl substance*[tiab] or total organic fluorine[tiab] or cadmium[tiab] or Chlorpyrifos[tiab] or lorsban[tiab] or dursban[tiab] or Algal Bloom Harmful[tiab] or Algal Blooms Harmful[tiab] or Bloom Harmful Algal[tiab] or Blooms Harmful Algal[tiab] or Harmful Algal Blooms[tiab] or Red Tide[tiab] or Red Tides[tiab] or Tide Red[tiab] or Tides Red[tiab] or Cyanobacteria[tiab] or Bacteria Blue Green[tiab] or Bacteria Blue-Green[tiab] or Blue Green Bacteria[tiab] or Algae Blue-Green[tiab] or Algae Blue Green[tiab] or Blue Green Algae[tiab] or Blue-Green Algae[tiab] or Cyanophyceae[tiab] or Blue-Green Bacteria[tiab] or Ciguatera[tiab] or Ciguatera Poisonings[tiab] or Poisoning Ciguatera[tiab] or Poisonings Ciguatera[tiab] or Ciguatera Fish Poisoning[tiab] or Ciguatera Fish Poisonings[tiab] or Poisoning Ciguatera Fish[tiab] or Poisonings Ciguatera Fish[tiab] or Ciguater[tiab] or Scombroid[tiab] or Domoic acid[tiab] or Hepatitis A viruses[tiab] or hepatitis A virus[tiab] or Salmonella[tiab] |  |
| 4 | "Blindness"[Mesh] OR "Neoplasms"[Mesh] OR "Mental Disorders"[Mesh] OR "Thyroid Diseases"[Mesh] OR "Hypersensitivity"[Mesh] OR "Mortality"[Mesh] OR "Psychomotor Performance"[Mesh] OR "Muscle Weakness"[Mesh] OR "Congenital, Hereditary, and Neonatal Diseases and Abnormalities"[Mesh] OR "Alzheimer Disease"[Mesh] OR "Language Development Disorders"[Mesh] OR "Ataxia"[Mesh] OR "Developmental Disabilities"[Mesh] OR "Attention Deficit Disorder with Hyperactivity"[Mesh] OR "Cell Proliferation"[Mesh] OR "Metacognition"[Mesh] OR "Motor Skills"[Mesh] OR "Cognition"[Mesh] OR "Hypotension"[Mesh] OR "Hypertension"[Mesh] OR "Growth Disorders"[Mesh] OR "Executive Function"[Mesh] OR "Growth and Development"[Mesh] OR "Asperger Syndrome"[Mesh] OR "Risk Assessment"[Mesh] OR "Oxidative Stress"[Mesh] OR "Apoptosis"[Mesh] OR "Outcome Assessment, Health Care"[Mesh] OR "Autoimmunity"[Mesh] OR "Tremor"[Mesh] OR "Thyroxine"[Mesh] OR "Thyroid Gland"[Mesh] OR "Shyness"[Mesh] OR "Sexual Maturation"[Mesh] OR "Reading"[Mesh] OR "Puberty"[Mesh] OR "Problem Solving"[Mesh] OR "Paresthesia"[Mesh] OR "Morbidity"[Mesh] OR "Mental Health"[Mesh] OR "Memory"[Mesh] OR "Inflammation"[Mesh] OR "Infant, Low Birth Weight"[Mesh] OR "Immunity"[Mesh] OR "Growth"[Mesh] OR "Goiter"[Mesh] OR "Depression"[Mesh] OR "Dementia"[Mesh] OR "Coronary Artery Disease"[Mesh] OR "Chronic Disease"[Mesh] OR "Child Development"[Mesh] OR "Central Nervous System"[Mesh] OR "Cell Differentiation"[Mesh] OR "Brain"[Mesh] OR "Blood Pressure"[Mesh] OR "Attention"[Mesh] OR "Asthma"[Mesh] OR "Anxiety"[Mesh] OR "Allergy and Immunology"[Mesh] OR "Acrodynia"[Mesh] OR "Memory and Learning Tests"[Mesh] OR "Central Nervous System Sensitization"[Mesh] OR "Child Behavior Disorders"[Mesh] OR "Fractures, Bone"[Mesh] OR "Risk Factors"[Mesh] OR "Depressive Disorder"[Mesh] OR "Autistic Disorder"[Mesh] OR "Vision Disorders"[Mesh] OR "Learning Disabilities"[Mesh] OR "Diabetes Mellitus, Type 2"[Mesh] OR "Child Nutritional Physiological Phenomena"[Mesh] OR "Frailty"[Mesh] OR "Hearing Loss"[Mesh] OR "Biomarkers"[Mesh] OR "Sleep Wake Disorders"[Mesh] OR "Kidney Diseases"[Mesh] OR "Cardiovascular Diseases"[Mesh] OR "Autoimmune Diseases"[Mesh] OR "Accidental Falls"[Mesh] OR "Academic Success"[Mesh] OR "Immune System Phenomena"[Mesh] OR "Vision, Low"[Mesh] OR "Irritable Mood"[Mesh] OR "Congenital Abnormalities"[Mesh] OR "Motor Skills Disorders"[Mesh] OR "Attention Deficit and Disruptive Behavior Disorders"[Mesh] OR "Central Nervous System Infections"[Mesh] OR "Brain Diseases"[Mesh] OR "Growth Charts"[Mesh] or Failure to Thrive[mh] or malnutrition[mh] or protein deficiency[mh] or Dyslipidemias[mh] or Cholesterol[mh] or "Cholesterol, LDL"[Mesh] or Triglycerides[mh] or "Lipoproteins, HDL"[Mesh] or mental disorder[tiab] or mental disorders[tiab] or cognitive[tiab] or neurocognitive[tiab] or neurodevelop*[tiab] or neurological[tiab] or Alzheimer*[tiab] or senility[tiab] or senile[tiab] or presenile[tiab] or motor skills[tiab] or attention-deficit-disorder[tiab] or attention-deficit-disorders[tiab] or ADHD[tiab] or Autism-Spectrum-Disorder[tiab] or Asperger[tiab] or academic performance[tiab] or academic failure[tiab] or academic success[tiab] or mental process*[tiab] or congenital abnormality[tiab] or congenital abnormalities[tiab] or birth defects[tiab] or birth defect[tiab] or chronic condition*[tiab] or changes-in-vision[tiab] or vision problem[tiab] or changes-in-hearing[tiab] or deafness[tiab] or gastrointestinal[tiab] or respiratory[tiab] or obstruction-of-visual-field[tiab] or pink disease[tiab] or renal disease*[tiab] or tubular injury[tiab] or cancer[tiab] or learning disabilit*[tiab] or communication*[tiab] or social-interactive-skills[tiab] or functional disabilities[tiab] or functional disability[tiab] or sensorineural defect[tiab] or sensorineural defects[tiab] or vision loss[tiab] or hearing loss[tiab] or psychological disorder*[tiab] or behavioral disorder*[tiab] or Asperge*[tiab] or coronary disease*[tiab] or cardiac death[tiab] or Growth-and-development[tiab] or diabetes-mellitus-Type-2[tiab] or Metabolic outcome*[tiab] or accidental fall*[tiab] or thyroid*[tiab] or triiodothyronine[tiab] or mental-growth-retardation[tiab] or immune-system-phenomena[tiab] or hazardous substances[tiab] or hazardous substance[tiab] or risk factors[tiab] or risk factor[tiab] or risk-benefit[tiab] or thyroid dysfunction[tiab] or thyroid defect[tiab] or thyroid defects[tiab] or pre-term[tiab] or neurodevelopmental disorder*[tiab] or cardiovascular disease*[tiab] or toenail biomarker[tiab] or neurotoxicity syndrome*[tiab] or academic achievement*[tiab] or acrodynia[tiab] or allergy[tiab] or anxiety[tiab] or apoptosis[tiab] or areflexia[tiab] or asthma[tiab] or ataxia[tiab] or atopy[tiab] or attention[tiab] or autism[tiab] or autoimmune disease*[tiab] or autoimmunity[tiab] or blood pressure[tiab] or cell differentiation[tiab] or cell proliferation[tiab] or central nervous system[tiab] or child behavior disorder*[tiab] or child behaviour disorder*[tiab] or child development[tiab] or child nutritional physiological phenomena[tiab] or child nutritional physiological phenomena[tiab] or chronic disease*[tiab] or cognition[tiab] or dementia[tiab] or depression[tiab] or developmental delay*[tiab] or developmental disorder*[tiab] or environmental disease[tiab] or disorders-of-environmental-origin[tiab] or executive function[tiab] or fine motor[tiab] or bone-fracture*[tiab] or fractures-bone[tiab] or goiter[tiab] or gross motor[tiab] or health hazard[tiab] or hypersensitivity[tiab] or hypertension[tiab] or hypotension[tiab] or immune function[tiab] or immune system phenomena[tiab] or immunity[tiab] or inflammation[tiab] or irritability[tiab] or kidney disease[tiab] or language delay*[tiab] or language processing[tiab] or low birth weight[tiab] or memory[tiab] or mental growth retardation[tiab] or mental health[tiab] or metacognition[tiab] or morbidity[tiab] or mortality[tiab] or motor coordination[tiab] or muscle weakness[tiab] or neoplasms[tiab] or neuropathy[tiab] or outcome assessment[tiab] or oxidative stress[tiab] or paresthesia[tiab] or prematurity[tiab] or premature birth[tiab] or problem solving[tiab] or psychomotor performance[tiab] or puberty[tiab] or reading[tiab] or risk assessment*[tiab] or school readiness[tiab] or sensitization[tiab] or sexual maturation[tiab] or sexual maturity[tiab] or shyness[tiab] or sleep disorder*[tiab] or social interactive skill*[tiab] or tremor[tiab] or tubular dysfunction[tiab] or tubular injury[tiab] or verbal memory[tiab] or vision loss[tiab] or vision problem[tiab] or weakness[tiab] or Failure to thrive[tiab] or malnutrition[tiab] or protein deficiency[tiab] or Dyslipidemias[tiab] or cholesterol[tiab] or Low Density Lipoprotein Cholesterol[tiab] or beta-Lipoprotein Cholesterol[tiab] or Cholesterol beta-Lipoprotein[tiab] or beta Lipoprotein Cholesterol[tiab] or LDL Cholesterol[tiab] or Cholesteryl Linoleate LDL[tiab] or LDL Cholesteryl Linoleate[tiab] or triglycerides[tiab] or Triacylglycerols[tiab] or Triacylglycerol[tiab] or Triglyceride[tiab] or HDL Lipoproteins[tiab] or High-Density Lipoprotein[tiab] or Lipoprotein High-Density[tiab] or High-Density Lipoproteins[tiab] or High Density Lipoproteins[tiab] or Lipoproteins High-Density[tiab] or alpha-Lipoproteins[tiab] or alpha Lipoproteins[tiab] or Heavy Lipoproteins[tiab] or Lipoproteins Heavy[tiab] or High Density Lipoprotein[tiab] or Density Lipoprotein High[tiab] or Lipoprotein High Density[tiab] or alpha-Lipoprotein[tiab] or alpha Lipoprotein[tiab] or alpha-1 Lipoprotein[tiab] |  |
| 5 | (#1 AND #2 AND #3 AND #4) |  |
| 6 | #5 NOT ("Animals"[Mesh] NOT ("Animals"[Mesh] AND "Humans"[Mesh])) NOT (editorial[ptyp] OR comment[ptyp] OR news[ptyp] OR letter[ptyp] OR review[ptyp] OR systematic review[ptyp] OR systematic review[ti] OR meta-analysis[ptyp] OR meta-analysis[ti] OR meta-analyses[ti] OR retracted publication[ptyp] OR retraction of publication[ptyp] OR retraction of publication[tiab] OR retraction notice[ti])  Filters: Publication date from 2000/01/01; English | 2,859 |
| 7 | (catarrhini[mh:noexp] OR cercopithecidae[mh] OR gorilla gorilla[mh] OR haplorhini[mh:noexp] OR hominidae[mh:noexp] OR hylobatidae[mh] OR pan paniscus[mh] OR pan troglodytes[mh] OR platyrrhini[mh] OR pongo[mh] OR primates[mh:noexp] OR strepsirhini[mh] OR tarsii[mh] or Muridae[mh] or Murinae[mh] or Macaca[mh] or ape[tiab] apeas[tiab] or macaca[tiab] or macaque[tiab] or allenopithecus[tiab] OR allocebus[tiab] OR alouatta[tiab] OR alouattinae[tiab] OR angwantibo*[tiab] OR anthropoid[tiab] OR anthropoidea[tiab] OR anthropoids[tiab] OR aotes[tiab] OR aotidae[tiab] OR aotinae[tiab] OR aotus[tiab] OR ape[tiab] OR apes[tiab] OR arctocebus[tiab] OR ateles[tiab] OR atelidae[tiab] OR atelinae[tiab] OR avahi[tiab] OR aye-aye*[tiab] OR baboon[tiab] OR baboons[tiab] OR bonobo[tiab] OR bonobos[tiab] OR brachyteles[tiab] OR bushbabies[tiab] OR bushbaby[tiab] OR cacajao[tiab] OR callibella[tiab] OR callicebinae[tiab] OR callicebus[tiab] OR callimico[tiab] OR callithrichid*[tiab] OR callithrichinae[tiab] OR callithrix[tiab] OR callitrichid[tiab] OR callitrichidae[tiab] OR callitrichide[tiab] OR callitrichids[tiab] OR callitrichinae[tiab] OR capuchin[tiab] OR capuchins[tiab] OR carlito syrichta[tiab] OR catarhine*[tiab] OR catarhini[tiab] OR catarrhina[tiab] OR catarrhine*[tiab] OR catarrhini[tiab] OR cebid[tiab] OR cebidae[tiab] OR cebids[tiab] OR cebinae[tiab] OR ceboidea[tiab] OR cebuella[tiab] OR cebus[tiab] OR cephalopachus[tiab] OR cercocebus[tiab] OR cercopithecid*[tiab] OR cercopithecinae[tiab] OR cercopithecine*[tiab] OR cercopithecini[tiab] OR cercopithecoid[tiab] OR cercopithecoidea[tiab] OR cercopithecoids[tiab] OR cercopithecus[tiab] OR cheirogaleidae[tiab] OR cheirogaleus[tiab] OR cheracebus[tiab] OR chimp[tiab] OR chimpanzee[tiab] OR chimpanzees[tiab] OR chimps[tiab] OR chiromyiformes[tiab] OR chiropotes[tiab] OR chlorocebus[tiab] OR colobidae[tiab] OR colobinae[tiab] OR colobine*[tiab] OR colobini[tiab] OR colobus*[tiab] OR cynomolgus[tiab] OR daubentonia[tiab] OR daubentoniidae[tiab] OR douc[tiab] OR doucs[tiab] OR erythrocebus[tiab] OR eulemur[tiab] OR euoticus[tiab] OR euprimate*[tiab] OR galagid*[tiab] OR galago[tiab] OR galagoides[tiab] OR galagonidae[tiab] OR galagos[tiab] OR gelada[tiab] OR geladas[tiab] OR gibbon[tiab] OR gibbons[tiab] OR gorilla[tiab] OR gorillas[tiab] OR grivet[tiab] OR grivets[tiab] OR guenon*[tiab] OR guereza*[tiab] OR hapalemur[tiab] OR haplorhine*[tiab] OR haplorhini[tiab] OR haplorrhine*[tiab] OR haplorrhini[tiab] OR hominid*[tiab] OR hominin[tiab] OR homininae[tiab] OR hominine[tiab] OR hominines[tiab] OR hominini[tiab] OR hominins[tiab] OR hominoidea[tiab] OR hoolock[tiab] OR howler*[tiab] OR hylobates[tiab] OR hylobatidae[tiab] OR indri[tiab] OR indridae[tiab] OR indriid*[tiab] OR indris[tiab] OR kipunji*[tiab] OR lagothrix[tiab] OR langur[tiab] OR langurs[tiab] OR lemur[tiab] OR lemurid*[tiab] OR lemuriform[tiab] OR lemuriformes[tiab] OR lemuriforms[tiab] OR lemurinae[tiab] OR lemuroidea[tiab] OR lemurs[tiab] OR leontideus[tiab] OR leontocebus[tiab] OR leontopithecus[tiab] OR lepilemur[tiab] OR lepilemurid*[tiab] OR lesula*[tiab] OR lophocebus[tiab] OR loriform[tiab] OR loriformes[tiab] OR lorinae[tiab] OR loris[tiab] OR lorises[tiab] OR lorisid*[tiab] OR lorisiform*[tiab] OR lorisinae[tiab] OR lorisoid*[tiab] OR lutung[tiab] OR lutungs[tiab] OR macaca[tiab] OR macaque's[tiab] OR macaque[tiab] OR macaques[tiab] OR malbrouck*[tiab] OR mandrill[tiab] OR mandrills[tiab] OR mandrillus[tiab] OR mangabey*[tiab] OR marmoset[tiab] OR marmosets[tiab] OR mico argentatus[tiab] OR mico chrysoleucos[tiab] OR mico emiliae[tiab] OR mico humilis[tiab] OR mico marcai[tiab] OR mico melanurus[tiab] OR mico rondoni[tiab] OR microcebus[tiab] OR miopithecus[tiab] OR mirza coquereli[tiab] OR mirza zaza[tiab] OR monkey[tiab] OR monkeys[tiab] OR muriqui*[tiab] OR nasalis larvatus[tiab] OR nomascus[tiab] OR nycticebus[tiab] OR oedipomidas[tiab] OR orang utan*[tiab] OR orang-utan*[tiab] OR orangutan*[tiab] OR oreonax[tiab] OR otolemur[tiab] OR pan paniscus[tiab] OR pan troglodytes[tiab] OR panin[tiab] OR panina[tiab] OR panins[tiab] OR papio[tiab] OR papionini[tiab] OR paragalago[tiab] OR perodicticinae[tiab] OR perodicticus[tiab] OR phaner[tiab] OR piliocolobus[tiab] OR pithecia[tiab] OR pithecidae[tiab] OR pitheciid*[tiab] OR pitheciinae[tiab] OR pithecinae[tiab] OR platyrhine*[tiab] OR platyrhini[tiab] OR platyrrhina[tiab] OR platyrrhine*[tiab] OR platyrrhini[tiab] OR plecturocebus[tiab] OR pongid*[tiab] OR ponginae[tiab] OR pongo[tiab] OR potto[tiab] OR pottos[tiab] OR presbytini[tiab] OR presbytis[tiab] OR primate[tiab] OR primates[tiab] OR procolobus[tiab] OR prolemur[tiab] OR propithecus[tiab] OR prosimian*[tiab] OR prosimii[tiab] OR pseudopotto[tiab] OR pygathrix[tiab] OR rhinopithecus[tiab] OR rungwecebus[tiab] OR saguinus[tiab] OR saimiri[tiab] OR saimiriinae[tiab] OR sapajus[tiab] OR sciurocheirus[tiab] OR semnopithecus[tiab] OR siamang[tiab] OR siamangs[tiab] OR sifaka[tiab] OR sifakas[tiab] OR simians[tiab] OR simias[tiab] OR simiiform*[tiab] OR strepsir*[tiab] OR surili*[tiab] OR symphalangus[tiab] OR talapoin*[tiab] OR tamarin[tiab] OR tamarins[tiab] OR tamarinus[tiab] OR tarsier[tiab] OR tarsiers[tiab] OR tarsiid*[tiab] OR tarsiiform*[tiab] OR tarsius[tiab] OR theropithecus[tiab] OR trachypithecus[tiab] OR uacari*[tiab] OR uakari[tiab] OR uakaris[tiab] OR varecia[tiab] OR vervet*[tiab]) |  |
| 8 | #5 AND #7 |  |
| 9 | #8 NOT (editorial[ptyp] OR comment[ptyp] OR news[ptyp] OR letter[ptyp] OR review[ptyp] OR systematic review[ptyp] OR systematic review[ti] OR meta-analysis[ptyp] OR meta-analysis[ti] OR meta-analyses[ti] OR retracted publication[ptyp] OR retraction of publication[ptyp] OR retraction of publication[tiab] OR retraction notice[ti])  Filters: Publication date from 2000/01/01; English | 8 |

**Database #3: Cochrane Central Register of Controlled Trials (CENTRAL)**

- Provider: Ovid
- Date(s) Searched: July 12, 2023
- Date range searched: From January 1, 2000
- Search Terms:

| EBM Reviews - Cochrane Central Register of Controlled Trials <June 2023> | | |
| --- | --- | --- |
| 1 | Brachyura/ or Perciformes/ or Decapodiformes/ or Flounder/ or Fish Flour/ or Mya/ or Bass/ or Gadiformes/ or Fish Proteins/ or Fish Products/ or Shellfish Proteins/ or Salmo salar/ or Tilapia/ or Tuna/ or Shellfish/ or Methylmercury Compounds/ or Cetacea/ or Gadus morhua/ or Bivalvia/ or Seafood/ or Catfishes/ or Pectinidae/ or Sharks/ or Salmonidae/ or Perches/ or Ostreidae/ or Astacoidea/ or Fishes/ or Fish Proteins, Dietary/ or Mercury Poisoning/ | 595 |
| 2 | (seafood or sea foods or sea food or sea-food or sea-foods or fish consumption or fishes or fish protein or fish proteins or fish product or fish products or fish meal or fish flour or fatty fish or shellfish or shellfish proteins or mercurialism or mercury poisoning or methylmercury or sharks or swordfish or tuna or salmonine or salmon or sardine* or sardines or gadiformes or pollock or flounder or cod or tilapia or shrimp or oyster or oysters or clams or scallops or crab or crabs or perciformes or mackerel or catfishes or trout or lobster or decapodiformes or squid or halibut or mahi mahi or crayfish or crawfish or anchovy or herring or rockfish or marine product or marine products or marlin or orange roughy or tile fish or whales or perch or walleye fish or lake trout or salmonid or catfish or sushi or cerviche or sashimi or gravlax or tuna tartare or seafood crudo or fluke crudo or fluke or carpaccio or e-la-ota or poke or hinava or gohu ikan or esqueixada or kelaguen or namero or kilawin or stroganina or yusheng or koi pla or kokoda or kuai or lakerda or larb pla or ota ika or tiradito or xato or umai or salmo salar or atlantic salmon or salmonids).ti,ab,kw. | 2611 |
| 3 | or/1-2 | 2842 |
| 4 | Phytic Acid/ or Food Analysis/ or Diet, Mediterranean/ or Taurine/ or Folic Acid/ or Eicosapentaenoic Acid/ or Zinc/ or Vitamin D/ or Selenium/ or Nutritional Status/ or Iron/ or Iodine/ or Food/ or Drinking/ or Diet/ or Choline/ or Calcium/ or Fatty Acids, Omega-3/ or Docosahexaenoic Acids/ or Dietary Supplements/ or Meals/ or Fatty Acids/ or Fish Proteins/ or Proteins/ or Nutritive Value/ or Recommended Dietary Allowances/ or Polyphenols/ or Diet, Healthy/ or Diet Records/ or Infant Nutritional Physiological Phenomena/ or Eating/ or Magnesium/ | 51955 |
| 5 | (diet or dietary supplement or dietary supplements or food or nutritional status or iron or folic acid or calcium or Zinc or phytate or phytic acid or polyphenol or polyphenols or fish species or fish tissue or fish intake or fish-rich diet or fatty acids or fatty acid or fish protein or mediterranean diet or omega-3 fatty acids or omega 3 fatty acids or omega-3 fatty acid or omega 3 fatty acid or fish consumption or eicosapentaenoic acid or docosahexaenoic acid or docosapentaenoic acid or meals or food intake or dietary intake or magnesium).ti,ab,kw. | 169979 |
| 6 | or/4-5 | 186058 |
| 7 | Hexachlorobenzene/ or Mercury/ or Heptachlor/ or Endrin/ or Dieldrin/ or DDT/ or Arsenic/ or Aldrin/ or Mercury Compounds/ or Mercury Poisoning/ or Cyclohexane Monoterpenes/ or Fluorocarbons/ or Nanofibers/ or Polychlorinated Biphenyls/ or Chlordan/ or Arsenic Poisoning/ or Microplastics/ or Dioxins/ or Methylmercury Compounds/ or Cadmium/ or lead/ or chlorpyrifos/ or Harmful Algal Bloom/ or Cyanobacteria/ or Ciguatera Poisoning/ or Hepatitis A virus/ or Salmonella/ or Escherichia coli/ | 1674 |
| 8 | (polyfluoroalkyl substance* or pfas or methylmercury compounds or methylmercury-toxicity or organomercury compound or organic mercury compound or mercury exposure* or organomercury or methylmercury-induced cytotoxicity or organomercurials or mercurial* or polychlorinated biphenyl or polychlorinated biphenyls or polychlorobiphenyl compounds or methylmercury or methyl mercury or methyl-mercury or mehg or monomethylmercury or methylquecksilber or dioxin or dioxins or inorganic mercury or pfos or pfoa or campheclor or toxaphene or dichlorodiphenyltrichlorethane or chlordane or chlordan or dieldrin or hexachlorobenzene or cyclohexane or brominated flame retardant* or diphenyl ether or diphenyl ethers or polybrominated diphenyl ether or polybrominated diphenyl ethers or aquatic toxicology or aquatic toxicity or aldrin or arsenic or dibenzofuran* or endrin or fluorocarbon* or furan* or heptachlor or microfiber* or microplastic* or nanofiber* or nanoplastic* or organic-fluorine* or polyfluoroalkyl substance* or total organic fluorine or cadmium or Chlorpyrifos or lorsban or dursban or Algal Bloom Harmful or Algal Blooms Harmful or Bloom Harmful Algal or Blooms Harmful Algal or Harmful Algal Blooms or Red Tide or Red Tides or Tide Red or Tides Red or Cyanobacteria or Bacteria Blue Green or Bacteria Blue-Green or Blue Green Bacteria or Algae Blue-Green or Algae Blue Green or Blue Green Algae or Blue-Green Algae or Cyanophyceae or Blue-Green Bacteria or Ciguatera or Ciguatera Poisonings or Poisoning Ciguatera or Poisonings Ciguatera or Ciguatera Fish Poisoning or Ciguatera Fish Poisonings or Poisoning Ciguatera Fish or Poisonings Ciguatera Fish or Ciguater or Scombroid or Domoic acid or Hepatitis A viruses or hepatitis A virus or Salmonella).ti,ab,kw. | 1961 |
| 9 | or/7-8 | 3331 |
| 10 | Blindness/ or Neoplasms/ or Mental Disorders/ or Thyroid Diseases/ or Hypersensitivity/ or Mortality/ or Psychomotor Performance/ or Muscle Weakness/ or Alzheimer Disease/ or Language Development Disorders/ or Ataxia/ or Developmental Disabilities/ or Attention Deficit Disorder with Hyperactivity/ or Cell Proliferation/ or Metacognition/ or Motor Skills/ or Cognition/ or Hypotension/ or Hypertension/ or Growth Disorders/ or Executive Function/ or Asperger Syndrome/ or Risk Assessment/ or Oxidative Stress/ or Apoptosis/ or Outcome Assessment, Health Care/ or Autoimmunity/ or Tremor/ or Thyroxine/ or Thyroid Gland/ or Shyness/ or Sexual Maturation/ or Reading/ or Puberty/ or Problem Solving/ or Paresthesia/ or Morbidity/ or Mental Health/ or Memory/ or Inflammation/ or Infant, Low Birth Weight/ or Immunity/ or Growth/ or Goiter/ or Depression/ or Dementia/ or Coronary Artery Disease/ or Chronic Disease/ or Child Development/ or Central Nervous System/ or Cell Differentiation/ or Brain/ or Blood Pressure/ or Attention/ or Asthma/ or Anxiety/ or Acrodynia/ or Central Nervous System Sensitization/ or Child Behavior Disorders/ or Fractures, Bone/ or Risk Factors/ or Depressive Disorder/ or Autistic Disorder/ or Vision Disorders/ or Learning Disabilities/ or Diabetes Mellitus, Type 2/ or Child Nutritional Physiological Phenomena/ or Frailty/ or Hearing Loss/ or Biomarkers/ or Sleep Wake Disorders/ or Kidney Diseases/ or Cardiovascular Diseases/ or Autoimmune Diseases/ or Accidental Falls/ or Academic Success/ or Immune System Phenomena/ or Vision, Low/ or Irritable Mood/ or Congenital Abnormalities/ or Motor Skills Disorders/ or Central Nervous System Infections/ or Brain Diseases/ or Growth Charts/ or Failure to Thrive/ or malnutrition/ or protein deficiency/ or Dyslipidemias/ or Cholesterol/ or "Cholesterol, LDL"/ or Triglycerides/ or "Lipoproteins, HDL"/ | 294130 |
| 11 | (mental disorder or mental disorders or cognitive or neurocognitive or neurodevelop* or neurological or Alzheimer* or senility or senile or presenile or motor skills or attention-deficit-disorder or attention-deficit-disorders or ADHD or Autism-Spectrum-Disorder or Asperger or academic performance or academic failure or academic success or mental process* or congenital abnormality or congenital abnormalities or birth defects or birth defect or chronic condition* or changes-in-vision or vision problem or changes-in-hearing or deafness or gastrointestinal or respiratory or obstruction-of-visual-field or pink disease or renal disease* or tubular injury or cancer or learning disabilit* or communication* or social-interactive-skills or functional disabilities or functional disability or sensorineural defect or sensorineural defects or vision loss or hearing loss or psychological disorder* or behavioral disorder* or Asperge* or coronary disease* or cardiac death or Growth-and-development or diabetes-mellitus-Type-2 or Metabolic outcome* or accidental fall* or thyroid* or triiodothyronine or mental-growth-retardation or immune-system-phenomena or hazardous substances or hazardous substance or risk factors or risk factor or risk-benefit or thyroid dysfunction or thyroid defect or thyroid defects or pre-term or neurodevelopmental disorder* or cardiovascular disease* or toenail biomarker or neurotoxicity syndrome* or academic achievement* or acrodynia or allergy or anxiety or apoptosis or areflexia or asthma or ataxia or atopy or attention or autism or autoimmune disease* or autoimmunity or blood pressure or cell differentiation or cell proliferation or central nervous system or child behavior disorder* or child behaviour disorder* or child development or child nutritional physiological phenomena or child nutritional physiological phenomena or chronic disease* or cognition or dementia or depression or developmental delay* or developmental disorder* or environmental disease or disorders-of-environmental-origin or executive function or fine motor or bone-fracture* or fractures-bone or goiter or gross motor or health hazard or hypersensitivity or hypertension or hypotension or immune function or immune system phenomena or immunity or inflammation or irritability or kidney disease or language delay* or language processing or low birth weight or memory or mental growth retardation or mental health or metacognition or morbidity or mortality or motor coordination or muscle weakness or neoplasms or neuropathy or outcome assessment or oxidative stress or paresthesia or prematurity or premature birth or problem solving or psychomotor performance or puberty or reading or risk assessment* or school readiness or sensitization or sexual maturation or sexual maturity or shyness or sleep disorder* or social interactive skill* or tremor or tubular dysfunction or tubular injury or verbal memory or vision loss or vision problem or weakness or Failure to thrive or malnutrition or protein deficiency or Dyslipidemias or cholesterol or Low Density Lipoprotein Cholesterol or beta-Lipoprotein Cholesterol or Cholesterol beta-Lipoprotein or beta Lipoprotein Cholesterol or LDL Cholesterol or Cholesteryl Linoleate LDL or LDL Cholesteryl Linoleate or triglycerides or Triacylglycerols or Triacylglycerol or Triglyceride or HDL Lipoproteins or High-Density Lipoprotein or Lipoprotein High-Density or High-Density Lipoproteins or High Density Lipoproteins or Lipoproteins High-Density or alpha-Lipoproteins or alpha Lipoproteins or Heavy Lipoproteins or Lipoproteins Heavy or High Density Lipoprotein or Density Lipoprotein High or Lipoprotein High Density or alpha-Lipoprotein or alpha Lipoprotein or alpha-1 Lipoprotein).ti,ab,kw. | 942555 |
| 12 | or/10-11 | 1018770 |
| 13 | 3 and 6 and 9 and 12 | 32 |
| 14 | limit 13 to (yr="2000 -Current" and english language) | 30 |
| 15 | catarrhini/ or cercopithecidae/ or gorilla gorilla/ or haplorhini/ or hominidae/ or hylobatidae/ or pan paniscus/ or pan troglodytes/ or platyrrhini/ or pongo/ or primates/ or strepsirhini/ or tarsii/ or Muridae/ or Murinae/ or Macaca/ | 137 |
| 16 | (gelada or geladas or gibbon or gibbons or gorilla or gorillas or grivet or grivets or guenon* or guereza* or hapalemur or haplorhine* or haplorhini or haplorrhine* or haplorrhini or hominid* or hominin or homininae or hominine or hominines or hominini or hominins or hominoidea or hoolock or howler* or hylobates or hylobatidae or indri or indridae or indriid* or indris or kipunji* or lagothrix or langur or langurs or lemur or lemurid* or lemuriform or lemuriformes or lemuriforms or lemurinae or lemuroidea or lemurs or leontideus or leontocebus or leontopithecus or lepilemur or lepilemurid* or lesula* or lophocebus or loriform or loriformes or lorinae or loris or lorises or lorisid* or lorisiform* or lorisinae or lorisoid* or lutung or lutungs or macaca or macaque's or macaque or macaques or malbrouck* or mandrill or mandrills or mandrillus or mangabey* or marmoset or marmosets or mico argentatus or mico chrysoleucos or mico emiliae or mico humilis or mico marcai or mico melanurus or mico rondoni or microcebus or miopithecus or mirza coquereli or mirza zaza or monkey or monkeys or muriqui* or nasalis larvatus or nomascus or nycticebus or oedipomidas or orang utan* or orang-utan* or orangutan* or oreonax or otolemur or pan paniscus or pan troglodytes or panin or panina or panins or papio or papionini or paragalago or perodicticinae or perodicticus or phaner or piliocolobus or pithecia or pithecidae or pitheciid* or pitheciinae or pithecinae or platyrhine* or platyrhini or platyrrhina or platyrrhine* or platyrrhini or plecturocebus or pongid* or ponginae or pongo or potto or pottos or presbytini or presbytis or primate or primates or procolobus or prolemur or propithecus or prosimian* or prosimii or pseudopotto or pygathrix or rhinopithecus or rungwecebus or saguinus or saimiri or saimiriinae or sapajus or sciurocheirus or semnopithecus or siamang or siamangs or sifaka or sifakas or simians or simias or simiiform* or strepsir* or surili* or symphalangus or talapoin* or tamarin or tamarins or tamarinus or tarsier or tarsiers or tarsiid* or tarsiiform* or tarsius or theropithecus or trachypithecus or uacari* or uakari or uakaris or varecia or vervet*).ti,ab,kw. | 1184 |
| 17 | or/15-16 | 1244 |
| 18 | 14 and 17 | 0 |

**Supplementary Table 2. Reasons for excluding articles during full text screening**

| **Author** | **Title** | **Reason for Exclusion** |
| --- | --- | --- |
| Abdallah (2013) | Bioaccumulation of heavy metals in mollusca species and assessment of potential risks to human health | Population |
| Abdel-Kader et al (2023) | Estimation of Cadmium in Muscles of Five Freshwater Fish Species from Manzalah Lake, and Possible Human Risk Assessment of Fish Consumption (Egypt) | Study design |
| Abdelouahab et al (2008) | Ecosystem matters: Fish consumption, mercury intake and exposure among fluvial lake fish-eaters | Intervention/exposure |
| Ahmad et al (2022) | Exposure Assessment of methyl mercury from consumption of fish and seafood in Peninsular Malaysia | Population |
| Ahmed et al (2013) | Seafood a potential source of some zoonotic bacteria in Zagazig, Egypt, with the molecular detection of Listeria monocytogenes virulence genes | Population |
| Airas et al (2004) | Copper, zinc, arsenic, cadmium, mercury, and lead in blue mussels (Mytilus edulis) in the Bergen harbor area, Western Norway | Population |
| Almerud et al (2021) | Cadmium, total mercury, and lead in blood and associations with diet, sociodemographic factors, and smoking in Swedish adolescents | Study design |
| Al-Saleh et al (2015) | The extent of mercury (Hg) exposure among Saudi mothers and their respective infants | Study design |
| Alves et al (2006) | Fish consumption, mercury exposure and serum antinuclear antibody in Amazonians | Study design |
| Alves et al (2017) | Mercury levels in parturient and newborns from Aveiro region, Portugal | Study design |
| Anderson et al (2008) | Polybrominated diphenyl ethers (PBDE) in serum: Findings from a US cohort of consumers of sport-caught fish | Population |
| Anh et al (2014) | Maternal risk factors associated with increased dioxin concentrations in breast milk in a hot spot of dioxin contamination in Vietnam | Population |
| Antignac et al (2013) | Occurrence of perfluorinated alkylated substances in breast milk of French women and relation with socio-demographical and clinical parameters: Results of the ELFE pilot study | Study design |
| Arakawa et al (2006) | Fish consumption and time to pregnancy in Japanese women | Population |
| Arisawa et al (2011) | Dietary patterns and blood levels of PCDDs, PCDFs, and dioxin-like PCBs in 1656 Japanese individuals | Population |
| Augustsson et al (2020) | Consumption of freshwater fish: a variable but significant risk factor for PFOS exposure | Population |
| Axmon (2006) | Menarche in women with high exposure to persistent organochlorine pollutants in utero and during childhood | Intervention/exposure |
| Axmon et al (2006) | Association between biomarkers of exposure to persistent organochlorine compounds (POCs) | Intervention/exposure |
| Axtell et al (2000) | Association between methylmercury exposure from fish consumption and child development at five and a half years of age in the Seychelles Child Development Study: an evaluation of nonlinear relationships | Study design |
| Baars et al (2004) | Dioxins, dioxin-like PCBs and non-dioxin-like PCBs in foodstuffs: Occurrence and dietary intake in the Netherlands | Population |
| Bakhshalizadeh et al (2023) | Health risk assessment of heavy metal concentration in muscle of Chelon auratus and Chelon saliens from the southern Caspian Sea | Study design |
| Barghi et al (2016) | Hexabromocyclododecane (HBCD) in the Korean food basket and estimation of dietary exposure | Study design |
| Barone et al (2021) | Polychlorinated dioxins, furans (PCDD/Fs) and dioxin-like polychlorinated biphenyls (dl-PCBs) in food from Italy: Estimates of dietaryintake and assessment | Population |
| Bassil et al (2018) | Lead, cadmium and arsenic in human milk and their socio-demographic and lifestyle determinants in Lebanon | Study design |
| Behrooz et al (2009) | Organochlorine pesticide and polychlorinated biphenyl residues in human milk from the Southern Coast of Caspian Sea, Iran | Study design |
| Bellanger et al (2000) | Blood mercury levels and fish consumption in Louisiana | Study design |
| Bilau et al (2008) | Dietary exposure to dioxin-like compounds in three age groups: Results from the Flemish environment and health study | Population |
| Bjerregaard et al (2001) | Exposure of inuit in greenland to organochlorines through the marine diet | Population |
| Bjornberg et al (2003) | Methyl mercury and inorganic mercury in Swedish pregnant women and in cord blood: Influence of fish consumption | Intervention/exposure |
| Bocca et al (2020) | Children exposure to inorganic and organic arsenic metabolites: A cohort study in Northeast Italy | Population |
| Bocio et al (2003) | Polybrominated diphenyl ethers (PBDEs) in foodstuffs: Human exposure through the diet | Population |
| Booth et al (2005) | Mercury, food webs, and marine mammals: Implications of diet and climate change for human health | Population |
| Boucher et al (2010) | Prenatal exposure to methylmercury and PCBs affects distinct stages of information processing: An event-related potential study with Inuit children | Population |
| Boucher et al (2012) | Response inhibition and error monitoring during a visual Go/No-Go task in Inuit children exposed to lead, polychlorinated biphenyls, and methylmercury | Study design |
| Boucher et al (2012) | Prenatal methylmercury, postnatal lead exposure, and evidence of attention deficit/hyperactivity disorder among Inuit children in Arctic Quebec | Study design |
| Boucher et al (2014) | Domain-specific effects of prenatal exposure to PCBs, mercury, and lead on infant cognition: Results from the environmental contaminants and child development study in nunavik | Population |
| Boucher et al (2016) | Altered fine motor function at school age in Inuit children exposed to PCBs, methylmercury, and lead | Intervention/exposure |
| Bourguignon et al (2011) | Multi-System Endocrine Disruption | Population |
| Brambilla et al (2013) | Mercury occurrence in Italian seafood from the Mediterranean Sea and possible intake scenarios of the Italian coastal population | Intervention/exposure |
| Brantsaeter et al (2010) | Exploration of biomarkers for total fish intake in pregnant Norwegian women | Intervention/exposure |
| Brustad et al (2023) | Diet-associated vertically transferred metabolites and risk of asthma, allergy, eczema, and infections in early childhood | Study design |
| Buchanan et al (2015) | Fish consumption and hair mercury among Asians in Chicago | Study design |
| Buck et al (2003) | Maternal fish consumption and infant birth size and gestation: New York State Angler Cohort Study | Study design |
| Butler et al (2017) | Childhood and adolescent fish consumption and adult neuropsychological performance: An analysis from the Cape Cod Health Study | Intervention/exposure |
| Buttriss (2004) | Oily fish: Still on the menu! | Study design |
| Cai et al (2015) | Assessment of exposure to heavy metals and health risks among residents near Tonglushan mine in Hubei, China | Study design |
| Callejo et al (2012) | A community-based approach to disseminate health information on the hazards of prenatal mercury exposure in Brooklyn, NY | Intervention/exposure |
| Carbonell-Barrachina et al (2012) | Essential and toxic elements in infant foods from Spain, UK, China and USA | Intervention/exposure |
| Cardoso et al (2010) | Methylmercury risks and EPA + DHA benefits associated with seafood consumption in Europe | Intervention/exposure |
| Cardoso et al (2018) | Portuguese preschool children: Benefit (EPA+DHA and Se) and risk (MeHg) assessment through the consumption of selected fish species | Population |
| Carroll (2013) | Fish and krill oil supplements: The good oil, a fishy tale or just the one that got away? | Study design |
| Cartier et al (2014) | Prenatal and 5-year p,p'-DDE exposures are associated with altered sensory processing in school-aged children in Nunavik: A visual evoked potential study | Intervention/exposure |
| Carvalho et al (2008) | High-fish consumption and risk prevention: Assessment of exposure to methylmercury in Portugal | Study design |
| Carvalho et al (2019) | Oxidative stress levels induced by mercury exposure in amazon juvenile populations in Brazil | Population |
| Carvalho et al (2022) | Quantitative risk-benefit assessment of Portuguese fish and other seafood species consumption scenarios | Population |
| Carwile et al (2016) | Childhood fish consumption and learning and behavioral disorders | Language |
| Castano et al (2015) | Fish consumption patterns and hair mercury levels in children and their mothers in 17 EU countries | Language |
| Ceccatto et al (2016) | Mercury distribution in organs of fish species and the associated risk in traditional subsistence villagers of the Pantanal wetland | Outcome |
| Cechova et al (2017) | Developmental neurotoxicants in human milk: Comparison of levels and intakes in three European countries | Intervention/exposure |
| Cediel Ulloa et al (2021) | Prenatal methylmercury exposure and DNA methylation in seven-year-old children in the Seychelles Child Development Study | Study design |
| Cerna et al (2020) | Biomonitoring of PFOA, PFOS and PFNA in human milk from Czech Republic, time trends and estimation of infant's daily intake | Study design |
| Chai et al (2021) | Bioaccessibility-corrected health risk of heavy metal exposure via shellfish consumption in coastal region of China | Intervention/exposure |
| Chan et al (2011) | Development and evaluation of a dynamic model that projects population biomarkers of methylmercury exposure from local fish consumption | Study design |
| Chang et al (2015) | Mercury exposure in healthy Korean weaning-age infants: Association with growth, feeding and fish intake | Population |
| Chang et al (2021) | Dietary exposure assessment of methylmercury and polyunsaturated fatty acids in saltwater fish and processed foods among Taiwanese women of child-bearing age and children: A novel core food-matching approach | Study design |
| Channa et al (2013) | Differences in prenatal exposure to mercury in South African communities residing along the Indian Ocean | Study design |
| Chao et al (2014) | Arsenic, cadmium, lead, and aluminium concentrations in human milk at early stages of lactation | Population |
| Chen et al (2020) | Risks of consuming cadmium-contaminated shellfish under seawater acidification scenario: Estimates of PBPK and benchmark dose | Population |
| Child Health Report (2007) | Seafood in pregnancy: good or bad for the offspring? | Population |
| Choi et al (2014) | Negative confounding by essential fatty acids in methylmercury neurotoxicity associations | Study design |
| Choi et al (2017) | Low-level toxic metal exposure in healthy weaning-age infants: Association with growth, dietary intake, and iron deficiency | Study design |
| Coccini et al (2010) | Assessing health effects of environmental contaminants by molecular markers. Studies on methylmercury and polychlorinated biphenyls as examples of translational research in environmental toxicology | Study design |
| Cohen et al (2005) | A quantitative analysis of prenatal methyl mercury exposure and cognitive development | Study design |
| Colles et al (2020) | Perfluorinated substances in the Flemish population (Belgium): Levels and determinants of variability in exposure | Study design |
| Collins (2012) | Synthetic Biology: Bits and pieces come to life | Population |
| Consumer Reports (2011) | Mercury in canned tuna still a concern: new tests reinforce a need for some people to limit consumption | Population |
| Copat et al (2013) | Heavy metals concentrations in fish and shellfish from eastern Mediterranean Sea: Consumption advisories | Population |
| Crepet et al (2005) | Management options to reduce exposure to methyl mercury through the consumption of fish and fishery products by the French population | Population |
| Croes et al (2014) | Health effects in the Flemish population in relation to low levels of mercury exposure: From organ to transcriptome level | Study design |
| Crump et al (2000) | Benchmark concentrations for methylmercury obtained from the Seychelles Child Development Study | Study design |
| Custodio et al (2020) | Total mercury in commercial fishes and estimation of Brazilian dietary exposure to methylmercury | Population |
| Dabeka et al (2003) | Survey of total mercury in total diet food composites and an estimation of the dietary intake of mercury by adults and children from two Canadian cities, 1998-2000 | Population |
| Dahmardeh Behrooz et al (2012) | Mercury concentration in the breast milk of Iranian women | Study design |
| Dai et al (2011) | Health risk assessment of organochlorine contaminants in fish from a major lake (Baiyangdian Lake) in North China | Study design |
| Darvill et al (2000) | Prenatal exposure to PCBs and infant performance on the fagan test of infant intelligence | Population |
| Davidson et al (2000) | Neurodevelopmental outcomes of Seychellois children from the pilot cohort at 108 months following prenatal exposure to methylmercury from a maternal fish diet | Study design |
| Davidson et al (2001) | Methylmercury and neurodevelopment: reanalysis of the Seychelles Child Development Study outcomes at 66 months of age | Study design |
| Davidson et al (2006) | Methylmercury and neurodevelopment: Longitudinal analysis of the Seychelles child development cohort | Study design |
| Davidson et al (2008) | Association between prenatal exposure to methylmercury and visuospatial ability at 10.7 years in the seychelles child development study | Study design |
| Davidson et al (2010) | Fish consumption, mercury exposure, and their associations with scholastic achievement in the Seychelles Child Development Study | Study design |
| Davidson et al (2011) | Fish consumption and prenatal methylmercury exposure: Cognitive and behavioral outcomes in the main cohort at 17 years from the Seychelles child development study | Population |
| De Burbure et al (2003) | Biomarkers of renal effects in children and adults with low environmental exposure to heavy metals | Study design |
| De Loma et al (2019) | Elevated arsenic exposure and efficient arsenic metabolism in indigenous women around Lake Poopo Bolivia | Study design |
| De Oliveira Santos et al (2002) | Exposure to mercury in the urban population of Rio Branco City, State of Acre, Brazil | Population |
| de Sousa Parreira et al (2022) | Mercury in the brain (tumor tissues) and in markers (hair and blood) of exposure in Western Amazonia patients | Population |
| de Souza et al (2011) | Shellfish from Todos os Santos Bay, Bahia, Brazil: Treat or threat? | Study design |
| DeCastro et al (2014) | Dietary sources of methylated arsenic species in urine of the United States population, NHANES 2003-2010 | Population |
| Dellinger et al (2020) | Risk-Benefit Modeling to Guide Health Research in Collaboration with Great Lakes Fish Consuming Native American Communities | Study design |
| Di Leo et al (2010) | Mercury and methylmercury contamination in Mytilus galloprovincialis from Taranto Gulf (Ionian Sea, Southern Italy): Risk evaluation for consumers | Study design |
| Diez et al (2009) | Prenatal and early childhood exposure to mercury and methylmercury in spain, a high-fish-consumer country | Study design |
| Dikme et al (2013) | The relation between blood lead and mercury levels and chronic neurological diseases in children | Study design |
| Diletti et al (2018) | Intake estimates of dioxins and dioxin-like polychlorobiphenyls in the Italian general population from the 2013-2016 results of official monitoring plans in food | Intervention/exposure |
| Domingo et al (2006) | Exposure to PBDEs and PCDEs associated with the consumption of edible marine species | Population |
| Donat-Vargas et al (2020) | Cardiovascular and cancer mortality in relation to dietary polychlorinated biphenyls and marine polyunsaturated fatty acids: a nutritional-toxicological aspect of fish consumption | Population |
| Dooley (2012) | The beat | Language |
| Dorea (2009) | Risks of mercury exposure related to gestational fish consumption: Beyond the sea | Language |
| Dorea et al (2005) | Hair mercury (signature of fish consumption) and cardiovascular risk in Munduruku and Kayabi Indians of Amazonia | Study design |
| Dorea et al (2011) | Speciation of methyl- and ethyl-mercury in hair of breastfed infants acutely exposed to thimerosal-containing vaccines | Study design |
| Dorea et al (2012) | Neurodevelopment of Amazonian infants: Antenatal and postnatal exposure to methyl- and ethylmercury | Outcome |
| Dorea et al (2014) | Milestone achievement and neurodevelopment of rural amazonian toddlers (12 to 24 months) with different methylmercury and ethylmercury exposure | Study design |
| Dougherty et al (2000) | Dietary exposures to food contaminants across the United States | Study design |
| Dovydaitis (2008) | Fish Consumption During Pregnancy: An Overview of the Risks and Benefits | Study design |
| Doyle (2012) | The doctor and the warlord: A fictional tale of moral ambiguity | Study design |
| Du et al (2021) | Monthly variations in mercury exposure of school children and adults in an industrial area of southwestern China | Study design |
| El-Said et al (2020) | Human Health Implication of Major and Trace Elements Present in Commercial Crustaceans of a Traditional Seafood Marketing Region, Egypt | Population |
| Emmett et al (2015) | Pregnancy diet and associated outcomes in the Avon Longitudinal Study of Parents and Children | Population |
| Endo et al (2010) | High mercury levels in hair samples from residents of Taiji, a Japanese whaling town | Study design |
| Falco et al (2006) | Daily intake of arsenic, cadmium, mercury, and lead by consumption of edible marine species | Population |
| Feinberg et al (2011) | Assessment of seasonality in exposure to dioxins, furans and dioxin-like PCBs by using long-term food-consumption data | Study design |
| Feingold et al (2020) | Population-based dietary exposure to mercury through fish consumption in the Southern Peruvian Amazon | Study design |
| Feng et al (2020) | Impact of low-level mercury exposure on intelligence quotient in children via rice consumption | Study design |
| Ferrante et al (2007) | Polychlorinated biphenyls and organochlorine pesticides in seafood from the gulf of Naples (Italy) | Study design |
| Ferrante et al (2022) | Microplastics in fillets of Mediterranean seafood. A risk assessment study | Population |
| Fillion et al (2006) | A preliminary study of mercury exposure and blood pressure in the Brazilian Amazon | Study design |
| Fitzgerald et al (2001) | The association between local fish consumption and DDE, mirex, and HCB concentrations in the breast milk of Mohawk women at Akwesasne | Study design |
| Fok et al (2007) | Fetal methylmercury exposure as measured by cord blood mercury concentrations in a mother-infant cohort in Hong Kong | Study design |
| Fonseca et al (2008) | Poor psychometric scores of children living in isolated riverine and agrarian communities and fish-methylmercury exposure | Population |
| Fonseca et al (2014) | Iron status as a covariate in methylmercury-associated neurotoxicity risk | Population |
| Freeman (2015) | Beware of methylmercury during pregnancy! | Population |
| Freire et al (2010) | Hair mercury levels, fish consumption, and cognitive development in preschool children from Granada, Spain | Study design |
| Friesema et al (2012) | Outbreak of salmonella Thompson in The Netherlands since July 2012 | Study design |
| Fromberg et al (2011) | Estimation of dietary intake of PCB and organochlorine pesticides for children and adults | Population |
| Fuentes-Gandara et al (2018) | Assessment of human health risk associated with methylmercury in the imported fish marketed in the Caribbean | Study design |
| Fujii et al (2021) | Estimation of dietary intake and sources of organohalogenated contaminants among infants: 24-h duplicate diet survey in Fukuoka, Japan | Study design |
| Gallo et al (2015) | Changes in persistent organic pollutant levels from adolescence to young adulthood | Study design |
| Gao et al (2014) | Risk and benefit assessment of potential neurodevelopmental effect resulting from consumption of marine fish from a coastal archipelago in China | Study design |
| Garcia-Hernandez et al (2018) | Mercury concentrations in seafood and the associated risk in women with high fish consumption from coastal villages of Sonora, Mexico | Study design |
| Gari et al (2013) | Influence of socio-demographic and diet determinants on the levels of mercury in preschool children from a Mediterranean island | Population |
| Gaxiola-Robles et al (2014) | Interaction between mercury (Hg), arsenic (As) and selenium (Se) affects the activity of glutathione S-transferase in breast milk; possible relationship with fish and sellfish intake | Study design |
| Gerner-Smidt et al (2009) | Sources of outbreaks of foodborne infections in different regions of the world | Population |
| Gilbert-Diamond et al (2013) | A population-based case-control study of urinary arsenic species and squamous cell carcinoma in New Hampshire, USA | Population |
| Gilman et al (2015) | Umbilical cord blood and placental mercury, selenium and selenoprotein expression in relation to maternal fish consumption | Population |
| Giordano et al (2010) | Maternal exposures to endocrine disrupting chemicals and hypospadias in offspring | Population |
| Goldblum et al (2006) | The Fort Totten mercury pollution risk assessment: A case history | Population |
| Gonzalez et al (2019) | Dietary intake of arsenic, cadmium, mercury and lead by the population of Catalonia, Spain: Analysis of the temporal trend | Population |
| Gonzalez et al (2021) | Dietary exposure to potentially toxic elements through sushi consumption in Catalonia, Spain | Study design |
| Graber et al (2013) | Ciguatera fish poisoning - New York city, 2010-2011 | Study design |
| Grandjean et al (2003) | Arachidonic acid status during pregnancy is associated with polychlorinated biphenyl exposure | Population |
| Grandjean et al (2003) | Neurotoxic risk caused by stable and variable exposure to methylmercury from seafood | Study design |
| Grandjean et al (2004) | Cardiac autonomic activity in methylmercury neurotoxicity: 14-Year follow-up of a Faroese birth cohort | Population |
| Green et al (2004) | Effects of long-term exposure of the red swamp crawfish Procambarus clarkii to a mixture of two herbicides, 2,4-dichlorophenoxyacetic acid and monosodium methanearsonate, and associated human health risks | Study design |
| Grzunov Letinic et al (2016) | Use of human milk in the assessment of toxic metal exposure and essential element status in breastfeeding women and their infants in coastal Croatia | Intervention/exposure |
| Gundacker et al (2012) | Fish consumption during pregnancy: Risk or benefit? | Intervention/exposure |
| Gundacker et al (2012) | The role of the placenta in fetal exposure to heavy metals | Population |
| Guo et al (2010) | Dietary intake and potential health risk of DDTs and PBDEs via seafood consumption in South China | Study design |
| Hackethal et al (2023) | Chronic dietary exposure to total arsenic, inorganic arsenic and water-soluble organic arsenic species based on results of the first German total diet study | Study design |
| Hamid et al (2019) | Effect of fish frequency consumption on serum mercury levels in pregnant mothers and their newborns | Population |
| Hanning et al (2003) | Impact on blood Pb levels of maternal and early infant feeding practices of First Nation Cree in the Mushkegowuk Territory of northern Ontario, Canada | Population |
| Hansen et al (2014) | Maternal concentrations of persistent organochlorine pollutants and the risk of asthma in offspring: Results from a prospective cohort with 20 years of follow-up | Population |
| Hashemi et al (2023) | Dietary exposure and risk assessment of polybrominated diphenyl ethers in the Republic of Korea: A nationwide study | Population |
| Helmfrid et al (2012) | Health effects and exposure to polychlorinated biphenyls (PCBs) and metals in a contaminated community | Study design |
| Herrera et al (2021) | The burden of disease of three food-associated heavy metals in clusters in the Danish population - Towards targeted public health strategies | Study design |
| Holloman et al (2010) | A community-based assessment of seafood consumption along the lower James River, Virginia, USA: Potential sources of dietary mercury exposure | Study design |
| Holtcamp (2012) | Shark fin consumption may expose people to Neurotoxic BMAA | Population |
| Hong et al (2016) | Low-level methylmercury exposure through rice ingestion in a cohort of pregnant mothers in rural China | Study design |
| Hruba et al (2012) | Blood cadmium, mercury, and lead in children: An international comparison of cities in six European countries, and China, Ecuador, and Morocco | Study design |
| Hsi et al (2014) | The neurological effects of prenatal and postnatal mercury/methylmercury exposure on three-year-old children in taiwan | Population |
| Hsu et al (2014) | DDE and PCB serum concentration in maternal blood and their adult female offspring | Study design |
| Huang et al (2005) | Exploring nonlinear association between prenatal methylmercury exposure from fish consumption and child development: Evaluation of the Seychelles Child Development Study nine-year data using semiparametric additive models | Study design |
| Huang et al (2007) | Associations of diet with body burden of dibenzo-p-dioxins and dibenzofurans (PCDD/Fs) and dioxin-like polychlorinated biphenyls (PCBs): Observations on pregnant women from central Taiwan | Study design |
| Huang et al (2017) | Influence of seafood and vitamin supplementation on maternal and umbilical cord blood mercury concentration | Population |
| Huang et al (2018) | Analysis of Nonlinear Associations between Prenatal Methylmercury Exposure from Fish Consumption and Neurodevelopmental Outcomes in the Seychelles Main Cohort at 17 Years | Study design |
| Hui et al (2016) | Prenatal dioxin exposure and neurocognitive development in Hong Kong 11-year-old children | Population |
| Hulin et al (2020) | Health risk assessment to dioxins, furans and PCBs in young children: The first French evaluation | Study design |
| Imm et al (2007) | Maternal recall of children's consumption of commercial and sport-caught fish: Findings from a multi-state study | Study design |
| Innis et al (2006) | Increased levels of mercury associated with high fish intakes among children from Vancouver, Canada | Population |
| Iszatt et al (2015) | Prenatal and postnatal exposure to persistent organic pollutants and infant growth: A pooled analysis of seven European birth cohorts | Study design |
| Jacobson et al (2015) | Relation of prenatal methylmercury exposure from environmental sources to childhood IQ | Study design |
| Jagodic et al (2020) | Selected elements and fatty acid composition in human milk as indicators of seafood dietary habits | Intervention/exposure |
| Jan et al (2008) | Dental caries in Faroese children exposed to polychlorinated biphenyls | Population |
| Jensen et al (2005) | Effects of breast feeding on neuropsychological development in a community with methylmercury exposure from seafood | Population |
| Jeong et al (2014) | Occurrence and exposure assessment of polychlorinated biphenyls and organochlorine pesticides from homemade baby food in Korea | Study design |
| Jiang et al (2014) | Fetal exposure to environmental neurotoxins in Taiwan | Study design |
| Jiang et al (2015) | Levels of arsenic pollution in daily foodstuffs and soils and its associated human health risk in a town in Jiangsu Province, China | Study design |
| Junque et al (2017) | Integrated assessment of infant exposure to persistent organic pollutants and mercury via dietary intake in a central western Mediterranean site (Menorca Island) | Study design |
| Junque et al (2022) | Environmental and dietary determinants of metal exposure in four-year-old children from a cohort located in an industrial area (Asturias, Northern Spain) | Study design |
| Kampouri et al (2023) | Associations of gestational and early-life exposure to toxic metals and fluoride with a diagnosis of food allergy or atopic eczema at 1 year of age | Population |
| Kang et al (2018) | Perfluoroalkyl acids in serum of Korean children: Occurrences, related sources, and associated health outcomes | Population |
| Kao et al (2022) | Associations of maternal food safety-related risk perceptions and protective behaviors with daily mercury intake and internal doses of Taiwanese women and their preschool children | Intervention/exposure |
| Karatela et al (2019) | Mercury exposure in mother-children pairs in a seafood eating population: Body burden and related factors | Population |
| Karjalainen et al (2013) | Estimated intake levels for Finnish children of methylmercury from fish | Study design |
| Karmaus et al (2004) | Maternal concentration of polychlorinated biphenyls and dichlorodiphenyl dichlorethylene and birth weight in Michigan fish eaters: A cohort study | Study design |
| Karp et al (2020) | Multidrug-Resistant Salmonella Serotype Anatum in Travelers and Seafood from Asia, United States | Study design |
| Kasai (2015) | Food safety measures in Japan | Population |
| Kim et al (2008) | Hair mercury concentrations of children and mothers in Korea: Implication for exposure and evaluation | Population |
| Kim et al (2011) | Total and methyl mercury in maternal and cord blood of pregnant women in Korea | Population |
| Kim et al (2011) | Mercury exposure monitoring for Korean schoolchildren: I. Influence of socioeconomic and demographic variables | Study design |
| Kim et al (2013) | Microbial diversity and prevalence of foodborne pathogens in cheap and junk foods consumed by primary schoolchildren | Population |
| Kim et al (2014) | Estimated long-term dietary exposure to lead, cadmium, and mercury in young Korean children | Study design |
| Kim et al (2015) | Low-level Mercury Exposure and Risk of Asthma in School-age Children | Population |
| Kim et al (2018) | Urinary trace metals individually and in mixtures in association with preterm birth | Population |
| Kim et al (2023) | Time-course trend and influencing factors for per- and polyfluoroalkyl substances in the breast milk of Korean mothers | Population |
| Kippler et al (2021) | Total mercury in hair as biomarker for methylmercury exposure among women in central Sweden- a 23 year long temporal trend study | Intervention/exposure |
| Kirincic et al (2019) | Lead and cadmium in foods/drinking water from Slovenian market/taps: Estimation of overall chronic dietary exposure and health risks | Population |
| Kishi et al (2011) | Cohort profile: The hokkaido study on environment and Children's Health in Japan | Population |
| Knobeloch et al (2005) | Fish consumption, advisory awareness, and hair mercury levels among women of childbearing age | Study design |
| Knobeloch et al (2006) | Methylmercury exposure in Wisconsin: A case study series | Study design |
| Kobal et al (2017) | Exposure to mercury in susceptible population groups living in the former mercury mining town of Idrija, Slovenia | Study design |
| Koenig et al (2013) | New insights into mercury bioaccumulation in deep-sea organisms from the NW Mediterranean and their human health implications | Study design |
| Kormos (2013) | On call. I keep reading about health benefits of eating fish, but I've also heard that fish contain high levels of mercury. Should I be concerned? | Study design |
| Kounis et al (2015) | Histamine induced coronary artery spasm, fish consumption and Kounis syndrome | Population |
| Kuehn et al (2011) | Clinical monosensitivity to salmonid fish linked to specific IgE-epitopes on salmon and trout beta-parvalbumins | Study design |
| Kulman (2001) | Pregnant women get no bologna--or shark or brie | Study design |
| Kunisue et al (2006) | Contamination status of persistent organochlorines in human breast milk from Japan: Recent levels and temporal trend | Population |
| Labunska et al (2015) | Human dietary intake of organohalogen contaminants at e-waste recycling sites in Eastern China | Study design |
| Lackner et al (2018) | Disease burden of methylmercury in the German birth cohort 2014 | Study design |
| Laird et al (2017) | Exposure and risk characterization for dietary methylmercury from seafood consumption in Kuwait | Study design |
| Lam et al (2013) | Long term neurocognitive impact of low dose prenatal methylmercury exposure in Hong Kong | Intervention/exposure |
| Lambertino et al (2011) | Uterine leiomyomata in a cohort of Great Lakes sport fish consumers | Intervention/exposure |
| Langeland et al (2017) | Mercury levels in human hair and farmed fish near artisanal and small-scale gold mining communities in the madre de dios River Basin, Peru | Intervention/exposure |
| Larranaga et al (2002) | Mercury intake associated with fish consumption in a cohort of Gipuzkoa, Basque Country, Spain | Intervention/exposure |
| Lee et al (2013) | Contamination of polychlorinated biphenyls and organochlorine pesticides in breast milk in Korea: Time-course variation, influencing factors, and exposure assessment | Study design |
| Lee et al (2018) | Dietary patterns related to exposure to persistent organic pollutants based on the Ewha Birth and Growth Cohort | Study design |
| Lee et al (2021) | Fish consumption is an indicator of exposure to non-dioxin like polychlorinated biphenyls in cumulative risk assessments based on a probabilistic and sensitive approach | Study design |
| Lehner et al (2020) | Fish consumption is associated with school performance in children in a non-linear way | Study design |
| Leino et al (2013) | Effects of docosahexaenoic acid and methylmercury on child's brain development due to consumption of fish by Finnish mother during pregnancy: a probabilistic modeling approach | Intervention/exposure |
| Lemire et al (2006) | Elevated blood selenium levels in the Brazilian Amazon | Intervention/exposure |
| Lemire et al (2011) | Selenium from dietary sources and motor functions in the Brazilian Amazon | Population |
| Li et al (2020) | Health Risk Assessment of Metals (Cu, Pb, Zn, Cr, Cd, As, Hg, Se) in Angling Fish with Different Lengths Collected from Liuzhou, China | Study design |
| Li et al (2023) | Trace elements in red swamp crayfish (Procambarus clarkii) in China: Spatiotemporal variation and human health implications | Intervention/exposure |
| Liang et al (2013) | Plasma mercury levels in Hong Kong residents: In relation to fish consumption | Study design |
| Liem et al (2000) | Exposure of populations to dioxins and related compounds | Population |
| Lignell et al (2011) | Large variation in breast milk levels of organohalogenated compounds is dependent on mother's age, changes in body composition and exposures early in life | Study design |
| Lignell et al (2013) | Prenatal exposure to polychlorinated biphenyls (PCBs) and polybrominated diphenyl ethers (PBDEs) may influence birth weight among infants in a Swedish cohort with background exposure: A cross-sectional study | Study design |
| Liu et al (2014) | Mercury contamination in fish and human hair from Hainan Island, South China Sea: Implication for human exposure | Study design |
| Liu et al (2020) | Significant elevation of human methylmercury exposure induced by the food trade in Beijing, a developing megacity | Intervention/exposure |
| Llobet et al (2007) | Human exposure to polychlorinated naphthalenes through the consumption of edible marine species | Population |
| Llorente Ballesteros et al (2020) | Evaluation of blood mercury and serum selenium levels in the pregnant population of the Community of Madrid, Spain | Study design |
| Loghmani et al (2022) | Risk assessment of trace element accumulation in two species of edible commercial fish Scomberoides commersonnianus and Cynoglossus arel from the northern waters of the Oman Sea | Study design |
| Lorán et al (2010) | Risk assessment of PCDD/PCDFs and indicator PCBs contamination in Spanish commercial baby food | Study design |
| Love et al (2017) | Finding vulnerable subpopulations in the Seychelles Child Development Study: Effect modification with latent groups | Study design |
| Love et al (2022) | Contribution of child ABC-transporter genetics to prenatal MeHg exposure and neurodevelopment | Population |
| Lu et al (2015) | Levels of polychlorinated dibenzo-p-dioxins/furans (PCDD/Fs) and dioxin-like polychlorinated biphenyls (DL-PCBs) in breast milk in Shanghai, China: A temporal upward trend | Population |
| Lu et al (2021) | Continental-scale spatial distribution, sources, and health risks of heavy metals in seafood: challenge for the water-food-energy nexus sustainability in coastal regions? | Population |
| Lynch et al (2011) | Varying coefficient function models to explore interactions between maternal nutritional status and prenatal methylmercury toxicity in the Seychelles Child Development Nutrition Study | Study design |
| Mahaffey (2005) | Mercury exposure: medical and public health issues | Study design |
| Mahfouz et al (2023) | Maternal Serum, Cord and Human Milk Levels of Per- and Polyfluoroalkyl Substances (PFAS), Association with Predictors and Effect on Newborn Anthropometry | Study design |
| Maitre et al (2018) | Urine Metabolic Signatures of Multiple Environmental Pollutants in Pregnant Women: An Exposome Approach | Study design |
| Mallongi et al (2015) | Mercury distribution and its potential environmental and health risks in aquatic habitat at artisanal buladu gold mine in Gorontalo Province, Indonesia | Study design |
| Man et al (2014) | DDTs in mothers' milk, placenta and hair, and health risk assessment for infants at two coastal and inland cities in China | Intervention/exposure |
| Mansilla-Rivera et al (2011) | Metal levels in fish captured in Puerto Rico and estimation of risk from fish consumption | Intervention/exposure |
| Manzano-Salgado et al (2016) | Variability of perfluoroalkyl substance concentrations in pregnant women by socio-demographic and dietary factors in a Spanish birth cohort | Population |
| Marin et al (2017) | Dietary exposure to trace elements and health risk assessment in the region of Valencia, Spain: a total diet study | Study design |
| Marinho et al (2014) | Mercury speciation in hair of children in three communities of the Amazon, Brazil | Study design |
| Marques et al (2007) | Maternal mercury exposure and neuro-motor development in breastfed infants from Porto Velho (Amazon), Brazil | Population |
| Marques et al (2012) | Role of methylmercury exposure (from fish consumption) on growth and neurodevelopment of children under 5 years of age living in a transitioning (tin-mining) area of the western Amazon, Brazil | Intervention/exposure |
| Marques et al (2013) | Fish consumption during pregnancy, mercury transfer, and birth weight along the Madeira river basin in Amazonia | Population |
| Marques et al (2014) | Perinatal multiple exposure to neurotoxic (lead, methylmercury, ethylmercury, and aluminum) substances and neurodevelopment at six and 24 months of age | Study design |
| Marques et al (2015) | Neurodevelopment outcomes in children exposed to organic mercury from multiple sources in a tin-ore mine environment in Brazil | Intervention/exposure |
| Marques et al (2016) | Neurodevelopment of Amazonian children exposed to ethylmercury (from Thimerosal in vaccines) and methylmercury (from fish) | Population |
| Marques et al (2019) | Data relating to maternal fish consumption, methylmercury exposure, and early child neurodevelopment in the traditional living of Western Amazonians | Population |
| Marrugo-Negrete et al (2013) | Relationship between mercury levels in hair and fish consumption in a population living near a hydroelectric tropical dam | Intervention/exposure |
| Marrugo-Negrete et al (2020) | Human health risk of methylmercury from fish consumption at the largest floodplain in Colombia | Intervention/exposure |
| Marti-Cid et al (2007) | Intake of chemical contaminants through fish and seafood consumption by children of Catalonia, Spain: Health risks | Outcome |
| Matilla-Santander et al (2017) | Exposure to Perfluoroalkyl Substances and Metabolic Outcomes in Pregnant Women: Evidence from the Spanish INMA Birth Cohorts | Outcome |
| Maurice-Bourgoin et al (2000) | Mercury distribution in waters and fishes of the upper Madeira rivers and mercury exposure in riparian Amazonian populations | Intervention/exposure |
| Maycock et al (2007) | Risk assessment of dietary exposure to methylmercury in fish in the UK | Population |
| McDowell et al (2004) | Hair mercury levels in U.S. children and women of childbearing age: Reference range data from NHANES 1999-2000 | Study design |
| McKean et al (2015) | Prenatal mercury exposure, autism, and developmental delay, using pharmacokinetic combination of newborn blood concentrations and questionnaire data: A case control study | Study design |
| McSorley et al (2018) | Associations of maternal immune response with MeHg exposure at 28 weeks' gestation in the Seychelles Child Development Study | Intervention/exposure |
| Mead (2008) | Contaminants in human milk: Weighing the risks against the benefits of breastfeeding | Population |
| Mendola et al (2005) | Birth defects risk associated with maternal sport fish consumption: Potential effect modification by sex of offspring | Population |
| Mener et al (2015) | Lead exposure and increased food allergic sensitization in U.S. children and adults | Study design |
| Mhungu et al (2023) | Estimation of the cumulative risks from dietary exposure to cadmium, arsenic, nickel, lead and chromium in Guangzhou, China | Study design |
| Miklavcic et al (2011) | Biomarkers of low-level mercury exposure through fish consumption in pregnant and lactating Slovenian women | Population |
| Minoia et al (2011) | Influence of selenium and mercury on age-related cataracts in the Brazilian Amazon | Intervention/exposure |
| Mol et al (2018) | Potential health risks due to heavy metal uptake via consumption of Thunnus thynnus from the northern Levantine Sea | Population |
| Monastero et al (2016) | Demographic Profiles, Mercury, Selenium, and Omega-3 Fatty Acids in Avid Seafood Consumers on Long Island, NY | Population |
| Monji et al (2022) | A comparison of the metals and metalloid levels in wild and cultured Capoeta damascina fish and assessment of its potential health risks to humans in Iran | Population |
| Montuori et al (2006) | Mercury speciation in the hair of pre-school children living near a chlor-alkali plant | Population |
| Moon et al (2010) | Intake and potential health risk of polycyclic aromatic hydrocarbons associated with seafood consumption in Korea from 2005 to 2007 | Population |
| Moon et al (2011) | Exposure assessment for methyl and total mercury from seafood consumption in Korea, 2005 to 2008 | Study design |
| Mørck et al (2015) | The Danish contribution to the European DEMOCOPHES project: A description of cadmium, cotinine and mercury levels in Danish mother-child pairs and the perspectives of supplementary sampling and measurements | Population |
| Moriarity et al (2020) | Subsistence fishing in the Eeyou Istchee (James Bay, Quebec, Canada): A regional investigation of fish consumption as a route of exposure to methylmercury | Population |
| Moriarity et al (2020) | Using a geographic information system to assess local scale methylmercury exposure from fish in nine communities of the eeyou istchee territory (james bay, quebec, canada) | Study design |
| Morisset et al (2013) | Probabilistic mercury multimedia exposure assessment in small children and risk assessment | Intervention/exposure |
| Muckle et al (2001) | Prenatal exposure of the northern Quebec Inuit infants to environmental contaminants | Study design |
| Murata et al (2004) | Effects of methylmercury on neurodevelopment in Japanese children in relation to the Madeiran study | Population |
| Murata et al (2006) | Subclinical effects of prenatal methylmercury exposure on cardiac autonomic function in Japanese children | Intervention/exposure |
| Murcia et al (2016) | Prenatal mercury exposure and birth outcomes | Study design |
| Myers et al (2000) | Secondary analysis from the Seychelles Child Development Study: the child behavior checklist | Population |
| Myers et al (2003) | Prenatal methylmercury exposure from ocean fish consumption in the Seychelles child development study | Population |
| Myers et al (2007) | Maternal fish consumption benefits children's development | Population |
| Myers et al (2009) | Postnatal exposure to methyl mercury from fish consumption: A review and new data from the Seychelles Child Development Study | Population |
| Naess et al (2020) | Effects of seafood consumption on mercury exposure in Norwegian pregnant women: A randomized controlled trial | Study design |
| Naji et al (2016) | Potential human health risk assessment of trace metals via the consumption of marine fish in Persian Gulf | Study design |
| Nakai et al (2004) | The Tohoku Study of Child Development: A cohort study of effects of perinatal exposures to methylmercury and environmentally persistent organic pollutants on neurobehavioral development in Japanese children | Population |
| Nakatani et al (2005) | Polychlorinated dibenzo-p-dioxins, polychlorinated dibenzofurans, and coplanar polychlorinated biphenyls in human milk in Osaka City, Japan | Study design |
| Nakayama et al (2019) | Blood mercury, lead, cadmium, manganese and selenium levels in pregnant women and their determinants: the Japan Environment and Children's Study (JECS) | Study design |
| Navas-Acien et al (2011) | Seafood intake and urine concentrations of total arsenic, dimethylarsinate and arsenobetaine in the US population | Study design |
| Neithercott (2011) | Fish tales: how to choose what goes on your plate | Population |
| Newland et al (2008) | Methylmercury and nutrition: Adult effects of fetal exposure in experimental models | Study design |
| Newman et al (2014) | PCBs and ADHD in Mohawk adolescents | Population |
| Ng et al (2013) | Mercury, APOE, and children's neurodevelopment | Population |
| Ng et al (2015) | Mercury, APOE, and child behavior | Intervention/exposure |
| Nicole (2012) | Lessons of the Elwha river: Managing health hazards during dam removal | Population |
| Nicole (2013) | Meeting the needs o f the people fish consumption rates in the Pacific northwest | Intervention/exposure |
| Nieboer et al (2017) | Body burdens, sources and interrelations of selected toxic and essential elements among the nine Cree First Nations of: Eeyou Istchee, James Bay region of northern Quebec, Canada | Intervention/exposure |
| Ninomiya et al (2005) | Reappraisal of somatosensory disorders in methylmercury poisoning | Intervention/exposure |
| Nunes et al (2014) | Children's health risk and benefits of fish consumption: Risk indices based on a diet diary follow-up of two weeks | Study design |
| Nystrom et al (2022) | Healthy eating index and diet diversity score as determinants of serum perfluoroalkyl acid (PFAA) concentrations in a national survey of Swedish adolescents | Population |
| Okati et al (2012) | Hair mercury concentrations of lactating mothers and breastfed infants in Iran (fish consumption and mercury exposure) | Study design |
| Okati et al (2018) | Hair mercury and risk assessment for consumption of contaminated seafood in residents from the coast of the Persian Gulf, Iran | Intervention/exposure |
| Olszowski et al (2016) | Cadmium Concentration in Mother's Blood, Milk, and Newborn's Blood and Its Correlation with Fatty Acids, Anthropometric Characteristics, and Mother's Smoking Status | Population |
| Oosthuizen et al (2001) | The impact of pollution from a mercury processing plant in KwaZulu-Natal, South Africa, on the health of fish-eating communities in the area: An environmental health risk assessment | Population |
| Orenstein et al (2014) | Prenatal organochlorine and methylmercury exposure and memory and learning in school-age children in communities near the new bedford harbor superfund site, Massachusetts | Population |
| Orlando et al (2014) | Associations between prenatal and recent postnatal methylmercury exposure and auditory function at age 19years in the Seychelles child development study | Intervention/exposure |
| Orlando et al (2023) | The association of auditory function measures with low-level methylmercury from oceanic fish consumption and mercury vapor from amalgam: The Seychelles Child Development Study Nutrition 1 Cohort | Population |
| Ortega-Garcia et al (2009) | Estimated intake levels of methylmercury in children, childbearing age and pregnant women in a Mediterranean region, Murcia, Spain | Intervention/exposure |
| Orun et al (2012) | Mercury exposure via breast-milk in infants from a suburban area of Ankara, Turkey | Intervention/exposure |
| Paliwoda et al (2016) | Benefits and risks associated with consumption of Great Lakes fish containing omega-3 fatty acids and polychlorinated biphenyls (PCBs) | Study design |
| Palumbo et al (2000) | Association between prenatal exposure to methylmercury and cognitive functioning in Seychellois children: a reanalysis of the McCarthy Scales of Children's Ability from the main cohort study | Study design |
| Pandion et al (2022) | Potential health risk caused by heavy metal associated with seafood consumption around coastal area | Intervention/exposure |
| Papadopoulou et al (2013) | Maternal dietary intake of dioxins and polychlorinated biphenyls and birth size in the Norwegian Mother and Child Cohort Study (MoBa) | Intervention/exposure |
| Papadopoulou et al (2013) | Maternal diet, prenatal exposure to dioxins and other persistent organic pollutants and anogenital distance in children | Study design |
| Papadopoulou et al (2014) | Maternal diet, prenatal exposure to dioxin-like compounds and birth outcomes in a European prospective mother-child study (NewGeneris) | Intervention/exposure |
| Pei et al (2019) | The Bioaccumulation and Tissue Distribution of Arsenic Species in Tilapia | Population |
| Perelló et al (2012) | Assessment of the temporal trend of the dietary exposure to PCDD/Fs and PCBs in Catalonia, over Spain: health risks | Study design |
| Perez et al (2019) | Biomonitoring of mercury in hair of children living in the Valencian Region (Spain). Exposure and risk assessment | Study design |
| Periard et al (2015) | Associations of baroreflex sensitivity, heart rate variability, and initial orthostatic hypotension with prenatal and recent postnatal methylmercury exposure in the seychelles child development study at age 19 years | Study design |
| Persson et al (2015) | Dietary supplements: Health from the ocean? | Study design |
| Pesch et al (2002) | Mercury concentrations in urine, scalp hair, and saliva in children from Germany | Study design |
| Pinheiro et al (2007) | Mercury pollution and childhood in Amazon riverside villages | Study design |
| Pino et al (2017) | Human biomonitoring data analysis for metals in an Italian adolescents cohort: An exposome approach | Population |
| Pinzon-Bedoya et al (2020) | Assessment of potential health risks associated with the intake of heavy metals in fish harvested from the largest estuary in Colombia | Study design |
| Pirkle et al (2015) | Examining the impact of a public health message on fish consumption in Bermuda | Intervention/exposure |
| Ponce et al (2000) | Use of quality-adjusted life year weights with dose-response models for public health decisions: A case study of the risks and benefits of fish consumption | Population |
| Qu et al (2022) | Bioaccumulation of mercury along continuous fauna trophic levels in the Yellow River Estuary and adjacent sea indicated by nitrogen stable isotopes | Study design |
| Rahayu et al (2016) | Concentration of Mercury in Cockles (Anadara granosa and A. antiquata) Harvested from Estuaries of Western Lombok, Indonesia, and Potential Risks to Human Health | Population |
| Rahbar et al (2020) | Interaction between a mixture of heavy metals (lead, mercury, arsenic, cadmium, manganese, aluminum) and GSTP1, GSTT1, and GSTM1 in relation to autism spectrum disorder | Study design |
| Reid et al (2013) | Maternal exposure to organochlorine pesticides in Western Australia | Study design |
| Renieri et al (2019) | Cadmium, lead and mercury in muscle tissue of gilthead seabream and seabass: Risk evaluation for consumers | Study design |
| Ricketts et al (2020) | Risk-Benefit Assessment for Total Mercury, Arsenic, Selenium, and Omega-3 Fatty Acids Exposure from Fish Consumption in Jamaica | Study design |
| Riviere et al (2019) | Dietary exposure to perfluoroalkyl acids, brominated flame retardants and health risk assessment in the French infant total diet study | Study design |
| Robson et al (2020) | Development and implementation of a method to assess food and nutrient intakes in the Seychelles Child Development Nutrition Study | Population |
| Roosens et al (2010) | Exposure of the Flemish population to brominated flame retardants: Model and risk assessment | Study design |
| Rudge et al (2008) | Serum dioxin levels in Sydney Harbour commercial fishers and family members | Population |
| Ruelas-Inzunza et al (2011) | Health risk associated to dietary intake of mercury in selected coastal areas of Mexico | Study design |
| Rylander et al (2000) | Medical and psychometric examinations of conscripts born to mothers with a high intake of fish contaminated with persistent organochlorines | Study design |
| Rylander et al (2000) | Lowered birth weight among infants born to women with a high intake of fish contaminated with persistent organochlorine compounds | Study design |
| Rylander et al (2007) | Weight and height at 4 and 7 years of age in children born to mothers with a high intake of fish contaminated with persistent organochlorine pollutants | Study design |
| Saint-Amour et al (2006) | Alterations of visual evoked potentials in preschool Inuit children exposed to methylmercury and polychlorinated biphenyls from a marine diet | Population |
| Saito et al (2020) | Prenatal and postnatal methyl mercury exposure in Niigata, Japan: adult outcomes | Intervention/exposure |
| Sakamoto et al (2002) | Declining risk of methylmercury exposure to infants during lactation | Population |
| Sakamoto et al (2004) | Maternal and fetal mercury and n-3 polyunsaturated fatty acids as a risk and benefit of fish consumption to fetus | Intervention/exposure |
| Sakamoto et al (2007) | Correlations between mercury concentrations in umbilical cord tissue and other biomarkers of fetal exposure to methylmercury in the Japanese population | Intervention/exposure |
| Sakamoto et al (2008) | Changes in mercury concentrations of segmental maternal hair during gestation and their correlations with other biomarkers of fetal exposure to methylmercury in the Japanese population | Population |
| Santos-Lima et al (2020) | Neuropsychological Effects of Mercury Exposure in Children and Adolescents of the Amazon Region, Brazil | Study design |
| Saoudi et al (2018) | Prenatal exposure to lead in France: Cord-blood levels and associated factors: Results from the perinatal component of the French Longitudinal Study since Childhood (Elfe) | Intervention/exposure |
| Schaefer et al (2019) | Mercury exposure, fish consumption, and perceived risk among pregnant women in coastal Florida | Study design |
| Schecter et al (2006) | Polybrominated diphenyl ether (PBDE) levels in an expanded market basket survey of U.S. food and estimated PBDE dietary intake by age and sex | Population |
| Schell Jr et al (2001) | PCBs and neurodevelopmental effects in Michigan children: An evaluation of exposure and dose characterization | Population |
| Sekovanić et al (2020) | Mercury Exposure Assessment in Mother-Infant Pairs from Continental and Coastal Croatia | Intervention/exposure |
| Seo et al (2020) | The relationship between mercury exposure indices and dietary intake of fish and shellfish in women of childbearing age | Intervention/exposure |
| Ser et al (2017) | Differences in the responses of three plasma selenium-containing proteins in relation to methylmercury-exposure through consumption of fish/whales | Population |
| Shahmohamadloo et al (2023) | Lake Erie fish safe to eat yet afflicted by algal hepatotoxins | Study design |
| Sheehan et al (2012) | Association of markers of chronic viral hepatitis and blood mercury levels in US reproductive-age women from NHANES 2001-2008: A cross-sectional study | Study design |
| Shen et al (2012) | Polychlorinated dibenzo-p-dioxins/furans (PCDD/Fs), polychlorinated biphenyls (PCBs), and polybrominated diphenyl ethers (PBDEs) in breast milk from Zhejiang, China | Intervention/exposure |
| Shin et al (2015) | Progressive risk assessment of polychlorinated biphenyls through a Total Diet Study in the Korean population | Population |
| Sirot et al (2012) | Dietary exposure to polychlorinated dibenzo-p-dioxins, polychlorinated dibenzofurans and polychlorinated biphenyls of the French population: Results of the second French Total Diet Study | Study design |
| Sirot et al (2018) | French infant total diet study: Exposure to selected trace elements and associated health risks | Population |
| Soon et al (2014) | Seafood consumption and umbilical cord blood mercury concentrations in a multiethnic maternal and child health cohort | Study design |
| Soto-Jimenez et al (2010) | Nonessential metals in striped marlin and indo-pacific sailfish in the Southeast Gulf of California, Mexico: Concentration and assessment of human health risk | Population |
| Sprong et al (2023) | A case study of neurodevelopmental risks from combined exposures to lead, methyl-mercury, inorganic arsenic, polychlorinated biphenyls, polybrominated diphenyl ethers and fluoride | Population |
| Sprowles et al (2022) | Associations of concurrent PCB and PBDE serum concentrations with executive functioning in adolescents | Study design |
| Spulber et al (2010) | Effects of maternal smoking and exposure to methylmercury on brain-derived neurotrophic factor concentrations in umbilical cord serum | Intervention/exposure |
| Stern et al (2003) | An assessment of the cord blood: Maternal blood methylmercury ratio: Implications for risk assessment | Intervention/exposure |
| Steuerwald et al (2000) | Maternal seafood diet, methylmercury exposure, and neonatal neurologic function | Study design |
| Stewart et al (2008) | The relationship between prenatal PCB exposure and intelligence (IQ) in 9-year-old children | Study design |
| Strain et al (2012) | Maternal PUFA status but not prenatal methylmercury exposure is associated with children's language functions at age five years in the Seychelles | Study design |
| Strain et al (2015) | Prenatal exposure to methyl mercury from fish consumption and polyunsaturated fatty acids: Associations with child development at 20 mo of age in an observational study in the Republic of Seychelles | Study design |
| Strain et al (2021) | Associations of prenatal methylmercury exposure and maternal polyunsaturated fatty acid status with neurodevelopmental outcomes at 7 years of age: results from the Seychelles Child Development Study Nutrition Cohort 2 | Intervention/exposure |
| Stravik et al (2023) | Biomarkers of seafood intake during pregnancy - Pollutants versus fatty acids and micronutrients | Study design |
| Strom et al (2011) | Nutritional and toxicological aspects of seafood consumption-An integrated exposure and risk assessment of methylmercury and polyunsaturated fatty acids | Outcome |
| Ström et al (2011) | Nutritional and toxicological aspects of seafood consumption--an integrated exposure and risk assessment of methylmercury and polyunsaturated fatty acids | Intervention/exposure |
| Sun et al (2006) | Dioxin concentration in human milk in Hebei province in China and Tokyo, Japan: Potential dietary risk factors and determination of possible sources | Population |
| Sun et al (2022) | Accumulation characteristics of polychlorinated dibenzo-p-dioxins and dibenzofurans and polychlorinated biphenyls in human breast milk from a seaside city of North China | Intervention/exposure |
| Suzuki et al (2010) | Neurobehavioral effects of prenatal exposure to methylmercury and PCBs, and seafood intake: Neonatal behavioral assessment scale results of Tohoku study of child development | Population |
| Taioli et al (2005) | Human exposure to dioxins through diet in Italy | Intervention/exposure |
| Takekuma et al (2004) | Levels of PCDDs, PCDFs and Co-PCBs in human milk in Saitama, Japan, and epidemiological research | Intervention/exposure |
| Tang et al (2009) | Dietary exposure of Hong Kong secondary school students to total mercury and methylmercury from fish intake | Intervention/exposure |
| Tatsuta et al (2018) | Methylmercury Exposure and Developmental Outcomes in Tohoku Study of Child Development at 18 Months of Age | Population |
| Tatsuta et al (2022) | Association between whole blood metallic elements concentrations and gestational diabetes mellitus in Japanese women: The Japan environment and Children's study | Study design |
| Theobald (2003) | Oily fish and pregnancy | Population |
| Thomas et al (2017) | Demographic and dietary risk factors in relation to urinary metabolites of organophosphate flame retardants in toddlers | Intervention/exposure |
| Thompson et al (2013) | Multiple environmental chemical exposures to lead, mercury and polychlorinated biphenyls among childbearing-aged women (NHANES 1999-2004): Body burden and risk factors | Population |
| Thurston et al (2007) | Does prenatal methylmercury exposure from fish consumption affect blood pressure in childhood? | Population |
| Thurston et al (2022) | Associations between time-weighted postnatal methylmercury exposure from fish consumption and neurodevelopmental outcomes through 24 years of age in the Seychelles Child Development Study Main Cohort | Study design |
| Tian et al (2011) | Mercury hair concentrations and dietary exposure among Inuit preschool children in Nunavut, Canada | Population |
| Tran et al (2004) | Combining Food Frequency and Survey Data to Quantify Long-Term Dietary Exposure: A Methyl Mercury Case Study | Study design |
| Traynor et al (2013) | Fish consumption patterns and mercury exposure levels among women of childbearing age in Duval County, Florida | Population |
| Tsang et al (2011) | Body burden of POPs of Hong Kong residents, based on human milk, maternal and cord serum | Intervention/exposure |
| Uemura et al (2008) | PCDDs/PCDFs and dioxin-like PCBs: Recent body burden levels and their determinants among general inhabitants in Japan | Intervention/exposure |
| Valent et al (2013) | Neurodevelopmental effects of low-level prenatal mercury exposure from maternal fish consumption in a Mediterranean cohort: study rationale and design | Intervention/exposure |
| Valera et al (2012) | Cardiac autonomic activity and blood pressure among Inuit children exposed to mercury | Intervention/exposure |
| van Wijngaarden et al (2006) | Benchmark concentrations for methyl mercury obtained from the 9-year follow-up of the Seychelles Child Development Study | Intervention/exposure |
| Van Wijngaarden et al (2014) | Prenatal exposure to methylmercury and LCPUFA in relation to birth weight | Intervention/exposure |
| van Wijngaarden et al (2017) | Methyl mercury exposure and neurodevelopmental outcomes in the Seychelles Child Development Study Main cohort at age 22 and 24 years | Population |
| Vecchi Brumatti et al (2021) | Impact of Methylmercury and Other Heavy Metals Exposure on Neurocognitive Function in Children Aged 7 Years: Study Protocol of the Follow-up | Intervention/exposure |
| Verger et al (2008) | Balancing the risk of dioxins and polychlorinated biphenyls (PCBs) and the benefit of long-chain polyunsaturated fatty acids of the n-3 variety for French fish consumers in western coastal areas | Study design |
| Vieira et al (2013) | Total and methyl-mercury in hair and milk of mothers living in the city of Porto Velho and in villages along the Rio Madeira, Amazon, Brazil | Population |
| Vieira et al (2013) | Mercury in scalp hair near the mid-atlantic ridge (MAR) in relation to high fish consumption | Study design |
| Vieira Rocha et al (2014) | Selenium status and hair mercury levels in riverine children from RondOnia, Amazonia | Population |
| Vollset et al (2019) | Concentration of mercury, cadmium, and lead in breast milk from Norwegian mothers: Association with dietary habits, amalgam and other factors | Study design |
| Wahlberg et al (2018) | Maternal polymorphisms in glutathione-related genes are associated with maternal mercury concentrations and early child neurodevelopment in a population with a fish-rich diet | Population |
| Wallin et al (2004) | Exposure to persistent organochlorine compounds through fish consumption and the incidence of osteoporotic fractures | Intervention/exposure |
| Walther et al (2022) | Ecotoxicology of mercury in burbot (Lota lota) from interior Alaska and insights towards human health | Population |
| Wang et al (2012) | Pollution level and human health risk assessment of some pesticides and polychlorinated biphenyls in Nantong of Southeast China | Intervention/exposure |
| Wang et al (2013) | Arsenic concentration in rice, fish, meat and vegetables in Cambodia: A preliminary risk assessment | Intervention/exposure |
| Wang et al (2014) | Postnatal exposure to methyl mercury and neuropsychological development in 7-year-old urban inner-city children exposed to lead in the United States | Intervention/exposure |
| Weinhouse et al (2017) | Hair mercury level is associated with anemia and micronutrient status in children living near artisanal and small-scale gold mining in the Peruvian Amazon | Study design |
| Weisskopf et al (2003) | Decreased sex ratio following maternal exposure to polychlorinated biphenyls from contaminated Great Lakes sport-caught fish: a retrospective cohort study | Study design |
| Weisskopf et al (2005) | Maternal exposure to Great Lakes sport-caught fish and dichlorodiphenyl dichloroethylene, but not polychlorinated biphenyls, is associated with reduced birth weight | Study design |
| Wells et al (2022) | Total Blood Mercury Predicts Methylmercury Exposure in Fish and Shellfish Consumers | Intervention/exposure |
| Whitworth et al (2012) | Perfluorinated compounds in relation to birth weight in the Norwegian Mother and Child Cohort Study | Population |
| Wickliffe et al (2021) | Exposure to total and methylmercury among pregnant women in Suriname: sources and public health implications | Population |
| Willows et al (2002) | Blood lead concentrations and iron deficiency in Canadian aboriginal infants | Population |
| Witczak et al (2021) | Endocrine-disrupting organochlorine pesticides in human breast milk: Changes during lactation | Intervention/exposure |
| Witczak et al (2022) | Changes in Polychlorinated Biphenyl Residues in Milk during Lactation: Levels of Contamination, Influencing Factors, and Infant Risk Assessment | Intervention/exposure |
| Wolff et al (2005) | Predictors of organochlorines in New York City pregnant women, 1998-2001 | Intervention/exposure |
| Wu et al (2007) | Human exposure to PBDEs: Associations of PBDE body burdens with food consumption and house dust concentrations | Intervention/exposure |
| Wu et al (2013) | The residual levels and health risks of hexachlorocyclohexanes (HCHs) and dichloro-diphenyl-trichloroethanes (DDTs) in the fish from Lake Baiyangdian, North China | Population |
| Wu et al (2013) | Umbilical cord blood mercury levels in China | Study design |
| Wu et al (2014) | Effect of low-level prenatal mercury exposure on neonate neurobehavioral development in China | Population |
| Wu et al (2018) | Levels, congener profiles, and dietary intake assessment of polychlorinated dibenzo-p-dioxins/dibenzofurans and dioxin-like polychlorinated biphenyls in beef, freshwater fish, and pork marketed in Guangdong Province, China | Study design |
| Wyatt et al (2017) | Spatial, temporal, and dietary variables associated with elevated mercury exposure in peruvian riverine communities upstream and downstream of artisanal and small-scale gold mining | Study design |
| Xiong et al (2020) | Transport of arsenolipids to the milk of a nursing mother after consuming salmon fish | Study design |
| Yaez et al (2002) | Levels of dichlorodiphenyltrichloroethane and deltamethrin in humans and environmental samples in malarious areas of Mexico | Population |
| Yaginuma-Sakurai et al (2009) | Assessment of exposure to methylmercury in pregnant Japanese women by FFQ | Population |
| Ye et al (2021) | Pollution evaluation and children's multimedia exposure of atmospheric arsenic deposition in the Pearl River Delta, China | Study design |
| Yeter et al (2016) | Ethnic Kawasaki disease risk associated with blood mercury and cadmium in U.S. children | Study design |
| Yimthiang et al (2019) | Screening for elevated blood lead levels and related risk factors among thai children residing in a fishing community | Population |
| Yoong (2006) | Heavy-metal meals of mercury | Intervention/exposure |
| Yorifuji et al (2013) | Visual evoked potentials in children prenatally exposed to methylmercury | Intervention/exposure |
| Yorifuji et al (2015) | Intrauterine Exposure to Methylmercury and Neurocognitive Functions: Minamata Disease | Study design |
| Yoshida et al (2000) | Assessment of human health risk of dioxins in Japan | Population |
| Young et al (2020) | Association between prenatal dietary methyl mercury exposure and developmental outcomes on acquisition of articulatory-phonologic skills in children in the Republic of Seychelles | Intervention/exposure |
| Yusa et al (2017) | Biomonitoring of mercury in hair of breastfeeding mothers living in the Valencian Region (Spain). Levels and predictors of exposure | Study design |
| Zamora-Arellano et al (2018) | Mercury Levels and Risk Implications Through Fish Consumption on the Sinaloa Coasts (Gulf of California, Northwest Mexico) | Population |
| Zand et al (2012) | Elemental content of commercial 'ready to-feed' poultry and fish based infant foods in the UK | Population |
| Zeilmaker et al (2013) | Fish consumption during child bearing age: A quantitative risk-benefit analysis on neurodevelopment | Study design |
| Zhang et al (2012) | Levels of polychlorinated biphenyls and organochlorine pesticides in edible shellfish from Xiamen (China) and estimation of human dietary intake | Intervention/exposure |
| Zhuang et al (2013) | Health risk assessment for consumption of fish originating from ponds near Dabaoshan mine, South China | Intervention/exposure |
| Zilversmit et al (2017) | Correlations of Biomarkers and Self-Reported Seafood Consumption among Pregnant and Non-Pregnant Women in Southeastern Louisiana after the Gulf Oil Spill: The GROWH Study | Intervention/exposure |
| Zmudzinska et al (2022) | Health Safety Assessment of Ready-to-Eat Products Consumed by Children Aged 0.5-3 Years on the Polish Market | Population |
